# Rapid identification of bovine MHCI haplotypes in genetically divergent cattle populations using next-generation sequencing

**DOI:** 10.1007/s00251-016-0945-7

**Published:** 2016-08-11

**Authors:** Deepali Vasoya, Andy Law, Paolo Motta, Mingyan Yu, Adrian Muwonge, Elizabeth Cook, Xiaoying Li, Karen Bryson, Amanda MacCallam, Tatjana Sitt, Philip Toye, Barend Bronsvoort, Mick Watson, W. Ivan Morrison, Timothy Connelley

**Affiliations:** 1 Division of Genetics and Genomics, The Roslin Institute, The University of Edinburgh, Easter Bush, Midlothian, EH25 9RG Scotland UK; 2 International Livestock Research Institute, P.O. Box 30709, Nairobi, 00100 Kenya; 3 Division of Infection and Immunity, The Roslin Institute, The University of Edinburgh, Easter Bush, Midlothian, EH25 9RG Scotland UK; 4 Trinity College Dublin, College Green, Dublin, 2 Ireland; 5 Department of Animal and Veterinary Sciences, The University of Vermont, 311 Terrill, 570 Main, Burlington, VT 05405-0148 USA

**Keywords:** Cattle, MHCI, Next-generation sequencing

## Abstract

The major histocompatibility complex (MHC) region contains many genes that are key regulators of both innate and adaptive immunity including the polymorphic MHCI and MHCII genes. Consequently, the characterisation of the repertoire of MHC genes is critical to understanding the variation that determines the nature of immune responses. Our current knowledge of the bovine MHCI repertoire is limited with only the Holstein-Friesian breed having been studied in any depth. Traditional methods of MHCI genotyping are of low resolution and laborious and this has been a major impediment to a more comprehensive analysis of the MHCI repertoire of other cattle breeds. Next-generation sequencing (NGS) technologies have been used to enable high throughput and much higher resolution MHCI typing in a number of species. In this study we have developed a MiSeq platform approach and requisite bioinformatics pipeline to facilitate typing of bovine MHCI repertoires. The method was validated initially on a cohort of Holstein-Friesian animals and then demonstrated to enable characterisation of MHCI repertoires in African cattle breeds, for which there was limited or no available data. During the course of these studies we identified >140 novel classical MHCI genes and defined 62 novel MHCI haplotypes, dramatically expanding the known bovine MHCI repertoire.

**Electronic supplementary material:**

The online version of this article (doi:10.1007/s00251-016-0945-7) contains supplementary material, which is available to authorized users.

## Introduction

The major histocompatibility complex (MHC) locus contains a large number of genes associated with antigen presentation, including the MHCI, MHCII and non-classical MHCI genes. Classical MHCI gene products bind short peptide fragments (9–11 amino acids) in a ‘peptide-binding groove’ formed by the extra-cellular α1 and α2 domains. The combined peptide-MHCI structure forms the ligand recognised by αβ T cell receptors (TRs) expressed by antigen-specific CD8+ T cells and also by killer cell immunoglobulin-like (and other) receptors expressed by NK cells (Guethlein et al. 2015). Consequently, MHCI genes play key roles in regulating both innate and adaptive immune responses. A critical feature of MHCI genes is their high level of polymorphism; for example in humans, >9000 alleles of three classical MHCI (HLA-A, HLA-B, HLA-C) have been described (Robinson et al. 2013). Most of the MHCI polymorphism is focused within the exons encoding the α1 and α2 domains that form the peptide-binding groove. This polymorphism ensures that at the population level, MHCI genes have the capacity to effectively present a diverse range of peptides from any potential pathogen. As MHCI genes have a critical role in determining the pool of peptides presented to C8+ T cells and thus those that have the potential to serve as epitopes, knowledge of these genes is fundamental to understanding CD8+ T cell immunity and related areas of applied research such as vaccinology.

Currently the publicly available IPD-MHC database contains 97 classical MHCI and 18 non-classical bovine MHCI alleles (http://www.ebi.ac.uk/ipd/mhc). Based on knowledge of the complement of expressed genes in MHC-homozygous animals and phylogenetic analyses of the relatively conserved region (exons 4 to 8) of available MHCI cDNA sequences, the bovine MHC region on chromosome 23 has been postulated to contain six distinct classical MHCI loci (Hammond et al. 2012; Holmes et al. 2003). MHCI alleles allocated to each of these putative loci have been demonstrated to encode functional proteins (Gaddum et al. 2003; Graham et al. 2008; Guzman et al. 2010; Guzman et al. 2008). In contrast to humans, the number of expressed bovine MHCI genes varies between haplotypes, ranging from 1 to 4, with varying permutations of the six loci represented in any given haplotype (Codner et al. 2012). Currently, 26 haplotypes (5 of which have variants, containing near-identical alleles) incorporating in total 53 of the classical MHCI alleles have been defined. Most of the IPD-MHC sequences are derived from European *Bos taurus* cattle with almost half (43 classical MHCI alleles) from Holstein-Friesian (Babiuk et al. 2007; Birch et al. 2006; Brown et al. 1989; Davies et al. 2006; Ellis et al. 1999; Ellis et al. 2005; Ellis et al. 1996; Ellis et al. 1998; Holmes et al. 2003), the predominant dairy breed in Europe and North America. In contrast, MHCI allele sequences from the other major lineages of domestic cattle–*Bos indicus* and African *B. taurus*–which are extensively reared in tropical regions are only represented by six sequences from the Boran breed and a single sequence derived from the N’Dama breed, respectively. *B. indicus* and *B. taurus* cattle are derived from separate domestication events and diverged somewhere between 210,000 and 850,000 years ago (Bradley et al. 1996; Loftus et al. 1994; MacHugh et al. 1997). Although European and African taurine lineages are derived from the same domestication event, substantial admixture of the ancestors of African *B. taurus* with wild African Aurochs has led to significant genetic divergence (Decker et al. 2014). Due to the complex origins of the global cattle population and its highly outbred nature, comparison of the MHCI allele repertoires of these three domestic cattle lineages is of evolutionary as well as immunological interest.

Bovine MHCI typing has traditionally been achieved through serological testing and/or use of MHCI-allele-specific PCR or a combination of these techniques (Ellis et al. 2005; Ellis et al. 1998). However, incomplete knowledge of the repertoire of bovine MHCI genes means that it is not possible to verify the absolute specificity of MHCI allele/haplotype-specific antibodies and PCR profiles and that for animals expressing uncharacterised MHCI genes, the currently available reagents are of limited use. Consequently, haplotype identification has ultimately depended on MHCI gene sequence analysis using conventional Sanger sequencing, which is costly and laborious when conducted on a large scale. This is especially true for poorly characterised cattle populations, for which sequencing of multiple sub-clones of PCR products obtained from each animal using pan-MHCI primers is required.

The application of next-generation-sequencing (NGS) technologies in recent years has revolutionised HLA research and diagnostic typing as reviewed in several recent articles (Cereb et al. 2015; De Santis et al. 2013; Erlich 2012; Gabriel et al. 2014; Hosomichi et al. 2015). Exon sequencing is the most common application of NGS in HLA typing (Gabriel et al. 2014), which for MHCI molecules is usually focused on exon 2 and exon 3, which encode the α1 and α2 domains forming the peptide-binding groove (Cereb et al. 2015; Lange et al. 2014). Outside of the human field NGS has also been applied to the characterisation of MHC repertoires in a number of mammalian and bird species (e.g. Dudley et al. 2014; Heimbruch et al. 2015; Oomen et al. 2013; Promerova et al. 2012; Sepil et al. 2012).

In this study we sought to develop a high-throughput NGS bovine MHCI genotyping protocol to facilitate a rapid and cost-effective way to characterise the MHCI repertoires of different cattle populations. The variable allele content of bovine MHCI and the relative paucity of sequence data presented several challenges to the application of NGS. In this study we designed sets of novel ‘pan-MHCI’ primers that would allow amplification and sequencing of all known bovine MHCI alleles using the Illumina MiSeq platform and established a bioinformatic pipeline that could comprehensively analyse the resulting data. Following validation on a cohort of Holstein-Friesian animals, we applied the protocol to examine the MHCI alleles expressed by cohorts of Boran (*B. indicus*) cattle from Kenya and a predominantly Fulani cohort (African *B. taurus* × *B. indicus*) cattle from Cameroon. The data presented demonstrates the system provides an effective, rapid and relatively cheap method to perform large-scale typing of bovine MHCI alleles from genetically diverse populations.

## Materials and methods

### Sampling

The samples used in this study came from (i) Holstein-Friesian heifer calves born on the University Of Edinburgh’s Langhill farm in 2012/2013, (ii) Fulani, Goudali and cross-breed cattle from multiple herds in different locations of the North West Region of Cameron and (iii) Boran cattle from the International Livestock Research Institute ranch at Kapiti, Kenya. The work was approved by The Roslin Institute Animal Welfare and Ethical Review Body and conducted under license and in accordance with the UK government Animal (Scientific Procedures) Act 1986, UK. Blood samples were collected by jugular venipuncture into EDTA vacutainers (BD Biosciences, Oxford, UK) and erythrocytes lysed by incubation in 5× volume of erythrocyte lysis buffer (0.144 M ammonium chloride/0.0175 M Tris pH 7.4) for 5 min at room temperature. The white blood cell (WBC) pellet was washed three times in PBS, total RNA was extracted using Tri-reagent (Sigma, Gillingham, UK) and cDNA generated using a Reverse Transcription Kit (Promega, Madison, WI, USA), both according to the manufacturers’ instructions.

### PCR amplification and sample preparation

An alignment of sequences of known bovine MHCI gene cDNAs, as presented in the IPD-MHC database (May 2014), was used to design a series of 3′ (for) and 5′ (rev) pan-MHCI primers. After preliminary validation of different permutations of 3′ and 5′ primers pairs (data not shown) sets of TCMHCfor1/TCMHCrev2 (For1/Rev2), TCMHCfor3/TCMHCrev1 (For3/Rev1) primers incorporating Illumina adaptors and multiplex identifier tags (MID) were obtained from IDT (Leuven, Belgium–see Supplementary Data 1). cDNA from individual animals was subject to PCR amplification in two separate reactions using either the For1/Rev2 or the For3/Rev1 primer pairs. For each sample primers using a unique combination of MID tags were used to allow subsequent de-multiplexing of the sequence data. PCRs were conducted using the Phusion High-Fidelity PCR kit (New England BioLabs, Hitchin, UK) with 50 μl reactions composed of Phusion HF amplification buffer, 3 % DMSO, 0.2 mM dNTPs, 25 pmol of 3′ and 5′ primers, 1 U Phusion Hot Start DNA polymerase and 1 μl cDNA. Cycling conditions were 98 °C for 30 s, 30 cycles of 98 °C for 10 s, at 57 °C for 20 s, 72 °C for 30 s, and a final extension period of 72 °C for 10 min. Following amplification, 5 μl of PCR products from each sample were pooled, purified using Agencourt AMPure XP Beads (Beckman Coulter, High Wycombe, UK) at a *v*/*v* ratio of 1:1 beads to PCR product and run on a 1.5 % agarose gel. Bands of the appropriate size (∼548 and ∼488 bp for For1/Rev2 and For3/Rev1 respectively) were extracted and purified using the Qiagen Gel extraction kit (Qiagen, Manchester, UK) and quantified using 260/280 nm absorption readings obtained from a Nanodrop spectrophotometer (Wilmington, DE, USA). As part of the validation process, cDNA from the Holstein-Friesian cohort samples were subject to PCR using BoLA-3*00201 and 2*01801-specific primers using methods described previously (Ellis et al. 1998) to verify using an established technique which animals expressed the BoLA-A10 and BoLA-A11 haplotypes respectively.

### Sequencing and bioinformatic analysis

**Fig. 1:**
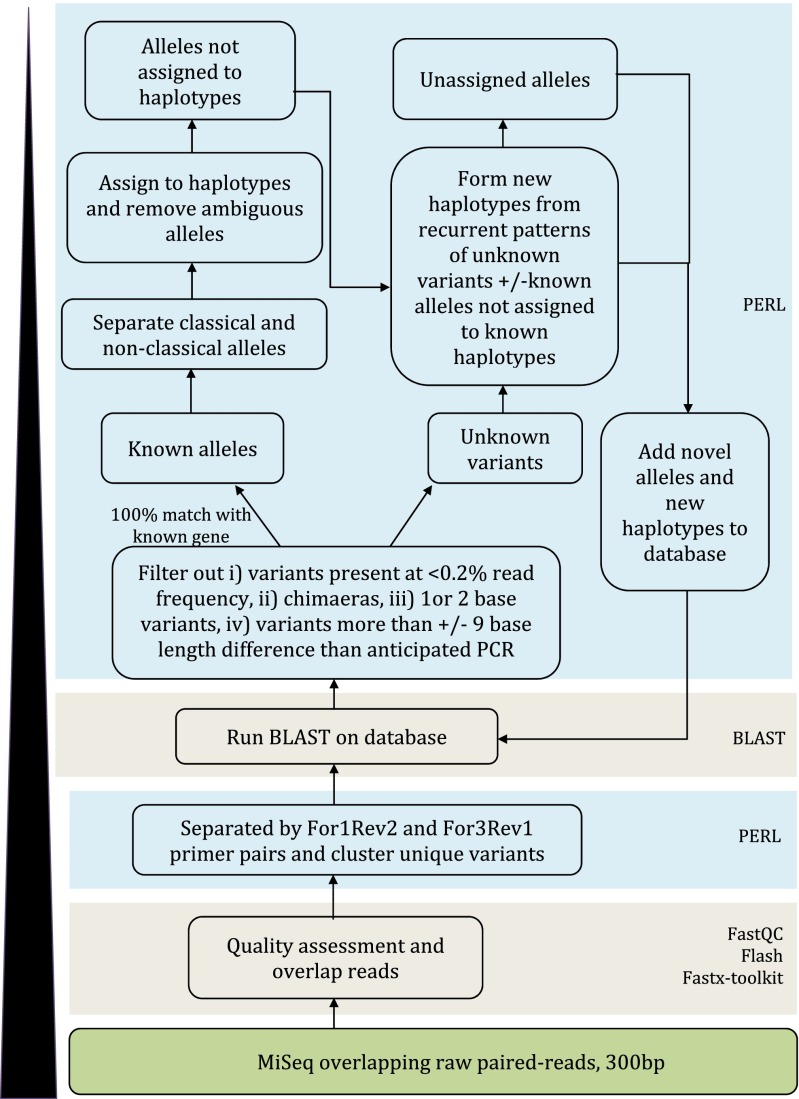
Schematic representation of the bioinformatics workflow developed to analyse the bovine MHCI data generated from the MiSeq sequencing of PCR amplicons. Some of the bioinformatic tools/scripting language used to perform the analyses are indicated

Libraries were submitted to Edinburgh Genomics where after standard quality control procedures they underwent 300 base paired-end sequencing on an Illumina MiSeq v3. All raw read sequence data have been submitted to the European nucleotide Archive (project number PRJEB14552). The sequence reads were segregated based on MID combinations into 96 datasets, the raw sequence data assessed for quality (threshold score of >Q_28_), and paired-end reads were overlapped using FLASH (Magoc and Salzberg 2011). Data were then processed using a bioinformatic analysis pipeline developed as part of this study, a simplified overview of which is provided in Fig. 1. In brief, data were separated into reads generated from For1Rev2 and For3Rev1 primer pairs, generating separate datasets for up to 192 samples (2 PCR × 96 MID). Within each sample, reads were clustered into unique variants which were subsequently compared using BLAST against a database of the previously identified bovine MHCI sequences. Variants were then discarded if (i) they represented <0.2 % of the reads in a sample; (ii) if they could be formed as a chimaera of two other, more frequent, variants in the same sample; (iii) if they were 1 or 2 base different from a variant present in the same sample at ≥30-fold or ≥50-fold read frequency, respectively; or (iv) if the length was > ±9 bp different from the anticipated length of the PCR product. Remaining variants with 100 % identity to a sequence in the database were described as ‘known alleles’, defined as classical or non-classical MHCI and, where appropriate, assigned to a previously defined haplotype. Remaining variants not matching a sequence in the database were described as ‘unknown variants’ were compared to the NCBI nucleotide database using BLAST to confirm if the nearest sequence was a bovini (i.e. domestic cattle or water buffalo) MHCI sequence, and given a unique identifier. Identification of novel haplotypes based on recurrent patterns of co-expressed sequences was conducted manually. Following each iteration of the analysis the MHCI sequence database and allele content of haplotypes was updated. The script developed for bioinformatic analysis has been submitted to the public repository Github (https://github.com/deepalivasoya/MHCIhaploCaller)

## Results

### Design of pan-bovine MHCI primer sets

**Fig. 2:**
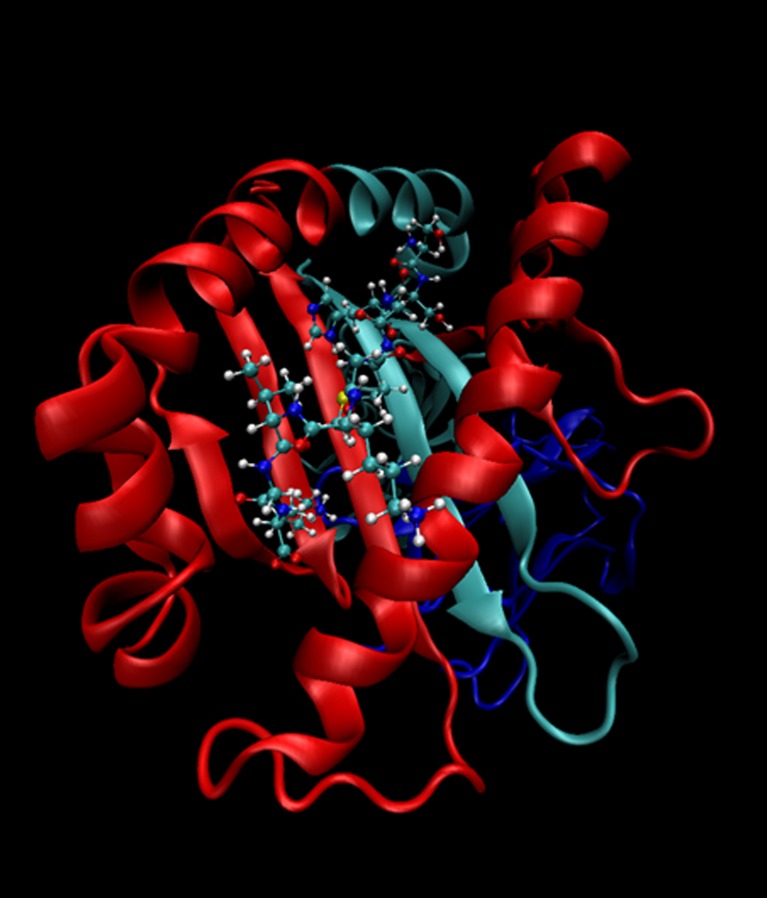
The 410 bp region amplified by the two PCR reactions combined encodes the majority of the peptide-binding groove. The peptide-MHCI structure of Tp1_214-224_ from *Theileria parva* Muguga bound to 6*0130i as determined by MacDonald et al. (2010) is shown as a ribbon representation. The section of the MHCI heavy chain encoded by the sequence amplified by the combined For1/Rev2 and For3/Rev1 primer pairs (shown in *red*) encompasses most of the peptide-binding groove, with only part of the beta-sheets at the N terminal end of the α1 and part of the alpha-helices at the C terminal end of the α2 domain excluded. The remainder of the heavy chain is shown in *light blue*, the β2-microgloulin as *dark blue* and the Tp1_214-224_ epitope is shown in a *stick and ball* representation. Figure prepared using VMD software

To enable analysis of bovine MHCI alleles using MiSeq, we sought to develop a PCR with the following characteristics: (i) allow amplification of all known classical bovine MHCI alleles, (ii) generate amplicons that following sequencing would permit unambiguous discrimination of the different MHCI alleles and (iii) generate amplicons no greater than 500 bp so that pair-end sequencing on the MiSeq platform would generate overlapping sequence data. Upon examination of the aligned sequences of the bovine MHCI genes in the IPD-MHC database, it was evident that design of a single primer pair satisfying all of these conditions was not feasible. Instead we designed two sets of 3′ and 5′ degenerate primers (For1/Rev2 and For3/Rev1) spanning most of the hypervariable exons 2 and 3 that encode the α1 and α2 domains. The length of the amplicons generated by these primer sets was anticipated to be 348 and 318 bp respectively and together cover 410 bp of exons 2 and 3, including most of the sequence encoding the peptide-binding groove (Fig. 2). In silico analysis demonstrated that (i) all of the known bovine MHCI sequences, with the exception of 1*06701, showed an absolute match to at least one of the primer sets and (ii) sequencing of the amplicons would allow alleles belonging to different ‘allele groups’ (i.e. groups of alleles which contain a maximum of four amino acid changes within the alpha 1 and 2 domains, plus up to four amino acid changes in any other parts of the coding sequence) to be discriminated with the exception 3*00402/3*05301 and within allele groups of all but four pairs of alleles (see Supplementary Data 2). Preliminary validation assays demonstrated that optimised PCRs using these primer pairs generated single bands of the correct sizes from cDNA generated from a panel of MHCI-genotyped animals and that upon sequencing the bands contained the anticipated MHCI alleles (data not shown).

### Validation of the NGS approach to MHCI typing and development of a bioinformatics pipeline

To validate the typing system, we first analysed a group of Holstein-Friesian heifers, the majority of which were expected to carry haplotypes for which sequence data were already available in the IPD-MHC database. Sequencing was performed on a cohort of 96 animals, which had been previously screened for expression of the 3*00201 and 2*01801 alleles (associated with the A10 and A11 haplotypes respectively) using allele-specific PCRs. For each animal cDNA derived from WBC was amplified using the For1/Rev2 and For3/Rev1 primer pairs, the products pooled, purified and submitted for sequencing on a single 300 base paired-end MiSeq run.

**Fig. 3:**
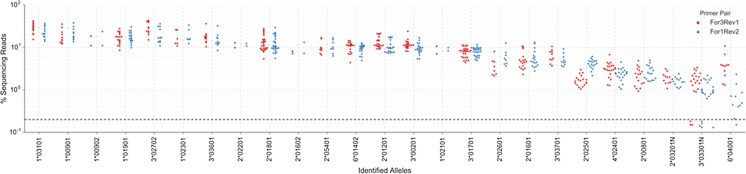
Read frequency of the known bovine MHCI alleles identified in the samples analysed in the first MiSeq run. A swarm plot is shown for the read frequency of each known bovine MHCI allele in each sample for both the For1/Rev2 and For3/Rev1 PCR reactions. Percentage (%) sequencing reads is shown on a logarithmic scale. The 0.2 % cut-off threshold is shown as a *bold horizontal dotted line*

Following quality control, a total of 1.1 × 10^7^ high-quality (>Q_28_) paired-end reads were obtained, the number of reads per sample (the product of a single PCR conducted on cDNA from one animal) varying from 7 × 10^3^ to 1.5 × 10^5^; this degree of coverage variation was anticipated as to enable a rapid high-throughput and cost-effective workflow, samples were not quantity normalised prior to pooling (Gabriel et al. 2014; Lange et al. 2014); however, read depth was sufficient for all samples to permit analysis. A summary of the output is provided in Table 1. A bioinformatic pipeline (Fig. 1) developed as part of this study corrected for various artefacts introduced during amplification/sequencing procedures, thus permitting analysis of the data. This pipeline included (i) a cut-off threshold to exclude variants (unique sequences) that constituted <0.2 % of reads in the sample that passed quality control filtering. This threshold was selected on the basis that, with the exception of 3*03301 N and 6*04001, all of the known MHCI alleles identified in the samples were present at >0.2 % frequency (Fig. 3) and reduction of the threshold to 0.1 % increased the number of artefacts (e.g. sequences present in only one sample that are likely to be erroneous) detected. All known classical MHCI alleles identified (with the exception of 3*03301 N and 6*04001) were consistently represented at a frequency of >0.9 % (Fig. 3), and thus the 0.2 % threshold was considered the optimal cut-off to allow sufficient margin for detection of genuine alleles expressed at low levels but exclusion of artefacts; (ii) an algorithm to detect and remove variants resulting from chimaera formation between more frequent variants within the same sample; (iii) removal of variants that had 1 or 2 bp differences from a variant that was present in the same sample but were present at <30 or <50-fold its frequency respectively, on the basis that these likely represented PCR/sequencing error; and (iv) removal of amplicons that were > ± 9 bp the anticipated product size (as these likely represented splice variants). Introduction of these parameters dramatically reduced the number of putative variants identified in each sample as summarised in Table 2.

**Table 1:** Summary of sequencing output from first MiSeq run

Parameter	Total for run	Read number per well^a^			
		Mean	SD	Max	Min
Number of reads	13,230,931	137,822	45,459	233,419	54,588
Number of reads passing QC and overlapping	11,538,900	120,197	40,410	208,711	48,757
Number of overlapping reads passing QC from For1/Rev2 PCR	4,424,638	50,277	31,116	136,476	7356
Number of overlapping reads passing QC from For3/Rev1 PCR	5,916,519	62,277	24,368	152,810	19,756

^a^Well refers to the two products for the For1/Rev2 and For3/Rev1 primers that shared the same MID combination

*SD* Standard deviation, *Max* maximum, *Min* minimum

**Table 2:** Effect of correction algorithms in bioinformatics pipeline on the numbers of reads and variants considered for MHCI analysis

		For1Rev2				For3Rev1			
		Mean	SD	Min	Max	Mean	SD	Min	Max
Passing QC and overlapping		*20,018* (50,277)	*12,813* (31,116)	*2405* (7356)	*59,304* (136,476)	*15,880* (62,277)	*5785* (24,368)	*6387* (19,756)	*36,414* (152,810)
Number after application of 0.2 % cut-off		*12* (22,378)	*3* (12,664)	*7* (4513)	*19* (51,137)	*22* (35,390)	*5* (15,670)	*10* (8934)	*34* (98,516)
Number removed afteranalysis for	Chimaeras	*2* (627)	*3* (965)	*0* (0)	*11* (3643)	*14* (3610)	*6* (2201)	*1* (149)	*29* (10,922)
	1 or 2 bp variants (PCR/sequencing error)	*1* (153)	*2* (248)	*0* (0)	*7* (1588)	*1* (117)	*1* (192)	*0* (0)	*5* (885)
	Sequences >9 bp different from anticipated length (splice variants)	*1* (227)	*1* (302)	*0* (0)	*4* (1619)	*1* (246)	*1* (294)	*0* (0)	*2* (1321)
Final number		*8* (21,371)	*1* (11,794)	*3* (4404)	*11* (47,173)	*8* (31,416)	*1* (14,632)	*3* (7965)	*12* (87,594)

Numbers of variants are shown in *italic script* with the number of reads shown in *parentheses* underneath

*SD* Standard deviation, *Max* maximum, *Min* minimum

The output from the bioinformatics pipeline consists of an excel workbook containing four separate worksheets and a set of fasta files for the relevant MHCI sequences identified. It is described in detail in Supplementary Data 3a and b. The pipeline automatically identifies previously defined bovine MHCI haplotypes when all of the composite alleles have been identified within a sample. However, manual annotation is required to construct novel MHCI haplotypes that are formed from either novel alleles or new combinations of known and/or novel alleles. To be considered a novel haplotype the combination of alleles was required to be present in at least two individuals. MHCI alleles considered to form part of a novel haplotype were given a number prefixed by ‘Roslin’ (e.g. Roslin 1.1 identifies the first novel allele identified in the first MiSeq run), whilst alleles which could not be assigned to a MHCI haplotype were given a number prefixed by ‘Unassigned’ (e.g. Unassigned 1.2 would relate to the second unassigned gene identified in MiSeq run1). Additional components of prefixes were used to describe alleles that (i) were only identified in one of the PCRs–(For1/Rev2) or (For3/Rev1) as appropriate (e.g. Roslin (For1Rev2).1.1 was only identified following amplification with the For1/Rev2 primers), (ii) differed from the anticipated read lengths by −SZ (e.g. RoslinSZ.1.1) and (iii) were considered from BLAST analysis to be non-classical MHCI alleles–NC (e.g. NCRoslin1.1). Different combinations of these prefixes were employed as necessary.

The results from the bioinformatics pipeline (Supplementary Data 4a) showed that 72 of the animals expressed two previously defined MHCI haplotypes, eight expressed one previously defined haplotype and no additional alleles (suggesting MHCI homozygosity), and six expressed three previously defined haplotypes (these were subsequently confirmed from breeding records as twins and so foetal chimaerism was considered to account for the expression of more than two MHCI haplotypes). The remaining ten animals all expressed one previously defined MHCI haplotype and one of three novel haplotypes (given temporary names haplotype (HP) 1.1, 1.2 and 1.3) which were identified in five, three and two animals respectively. All animals previously characterised as carrying the BoLA-A10 and BoLA-A11 haplotypes using the relevant allele-specific PCR were identified as such (data not shown). The allele composition of the three novel haplotypes is shown in Table 3. In addition, our analysis identified new alleles associated with four of the previously defined haplotypes–A10, A13, BF1 and H5 (Table 3). Thus, the analysis identified a total of 15 MHCI haplotypes (12 previously defined and 3 novel) which included 37 classical MHCI alleles (25 known and 12 novel). Notably, Roslin 1.1 (A10), RoslinSZ1.1 (A10), Roslin1.2 (A13) and 6*04001 (HP1.2) were not consistently present above the cut-off threshold (manual checking verified the alleles were present at sub-threshold levels), representing a form of ‘partial allele dropout’ (see Table 3 and Supplementary Data 5 Table 1a). Although the non-classical MHCI genes were not considered in the design of the PCR primers, the non-classical 1 gene (NC1) was amplified by both For1/Rev2 and For3/Rev1 reactions in many animals and three new variants were identified (NCRoslin1.1, 1.2 and 1.3) as well as two putative non-functional NC2 genes−NCRoslinSZ (For1Rev2).1.1 and NCRoslinSZ (For1Rev2).1.2 (Supplementary Data 4a).

**Table 3:** Allele content of bovine MHCI haplotypes sequenced in this study. The table describes the allele content of each of the bovine MHCI haplotypes identified in the course of this study and the number of times each of the haplotypes was identified in the three cohorts of animals examined. # Haplotypes in which at least one allele was only identified following either For1/Rev2 or For3/Rev1 PCR amplification (i.e. there was complete allele dropout in one of the primer sets–the relevant alleles can be identified from the nomenclature). † Haplotypes in which at least one allele was not consistently above the 0.2 % read frequency cut-off threshold (partial allele dropout); the relevant alleles are also marked with †

						Cohort		
						Holstein-Friesian	Boran	Cameroonian
Haplotype	Alleles					MiSeq Run1	MiSeq Run2	MiSeq Run3
A10†	3*00201	2*01201	Roslin1.1†	RoslinSZ.1.1†		27		
A11†	3*01701	3*03301N†	2*01801			38		
A12 (w12B)	1*01901	2*00801				19	4	2
A13†	1*03101	2*03201N	Roslin1.2†			15		
A14	1*02301	4*02401	2*02501	6*04001		9		
A15	1*00901	4*02401	2*02501			14		
A15v	1*00902	4*02401	2*02501	6*04001		3	1	6
A19	6*01402	2*01601				21		
A20 (v2)	3*02702	2*02601				8		
A31	1*02101	2*02201				2		
BF1	2*05401	Roslin1.3	Roslin1.4			10		
H5 (New5)	3*03601	3*03701	Roslin1.5			12		
HP1.1	Roslin1.6	4*02401				5		
HP1.2†	Roslin1.7	Roslin1.8	3*02702	6*04001†		3		
HP1.3#	Roslin1.9	Roslin1.10	2*01602	Roslin (For1Rev2).1.1		2		
HP2.1#	Roslin2.1	Roslin2.2	3*00402	Roslin (For3Rev1).2.1			36	
HP2.2	Roslin2.3	Roslin2.4					34	15
HP2.3	Roslin2.5	Roslin2.6	Roslin2.7				7	
HP2.4	Roslin2.8	Roslin2.9	Roslin2.10				8	3
HP2.5	Roslin2.11	Roslin2.12	3*00402				5	
HP2.6#	Roslin2.13	Roslin (For3Rev1).2.2					7	5
HP2.7	Roslin2.14	Roslin2.15	Roslin2.16				4	
HP2.8†	Roslin2.17	Roslin2.18	Roslin2.19†				12	3
HP2.9†	Roslin2.20	3*00102	RoslinSZ.2.1†				10	
HP2.10	Roslin2.21	Roslin2.22					4	
HP2.11†	Roslin2.23	Roslin2.24	Roslin2.19†				8	
HP2.12†	Roslin2.25	Roslin2.26	Roslin2.27†				5	
HP2.13†	Roslin2.28	4*02401	2*02501	6*04001†			9	
HP2.14	Roslin2.29	Roslin2.30	2*06001				2	3
HP2.15†	Roslin2.31	3*00102	RoslinSZ.2.1†				2	4
HP2.16	Roslin2.32	3*05901					4	4
HP2.17	Roslin2.33	Roslin2.12	3*00402				2	
HP2.18	Roslin2.34	Roslin2.36	Roslin2.35				4	
HP2.19#	Roslin2.47	Roslin2.48	Roslin2.49	Roslin (For3Rev1).2.4			2	
HP2.20	Roslin2.37	Roslin2.38					2	
HP2.21	Roslin2.39	Roslin2.40	Roslin2.41				2	
HP2.22†	Roslin2.42	RoslinSZ.3.2†					2	4
HP2.23	1*02801	4*02401					8	
HP2.24	Roslin2.39	Roslin2.40	Roslin2.43				3	
HP2.25	Roslin2.39	Roslin2.44	Roslin2.43				2	
HP2.26#	Roslin2.45	Roslin (For3Rev1).2.2					1	6
HP2.27#	Roslin2.46	Roslin (For3Rev1).2.3					2	7
HP3.1	Roslin3.1	Roslin3.2	Roslin3.3					5
HP3.2	Roslin3.5	Roslin3.4	3*03701					3
HP3.3	Roslin3.6	Roslin2.39	Roslin2.40					3
HP3.4	Roslin3.7	2*04701	2*03201N					7
HP3.5	Roslin3.8	Roslin3.9						2
HP3.6†	Roslin3.10	Roslin3.11	Roslin1.1†					2
HP3.7	Roslin3.12	2*01601						2
HP3.8	Roslin3.13	Roslin3.2						7
HP3.9	Roslin3.14	Roslin3.1						2
HP3.10	Roslin3.15	Roslin3.16						2
HP3.11	Roslin3.17							2
HP3.12	Roslin1.4	Roslin3.18	4*06301					5
HP3.13	Roslin3.19							2
HP3.14##	Roslin3.20	Roslin (For3Rev1).3.2	Roslin (For3Rev1).2.4	RoslinSZ (For1Rev2).3.1				2
HP3.15	Roslin3.5	Roslin3.21	3*03701					3
HP3.16	Roslin2.34	Roslin3.22	Roslin2.35					4
HP3.17	Roslin3.24	Roslin2.39	Roslin2.40					2
HP3.18#	Roslin3.25	Roslin3.26	Roslin (For3Rev1).2.3					3
HP3.19	Roslin3.29	Roslin3.27	Roslin3.28					4
HP3.20†	Roslin2.35	Roslin3.30	Roslin3.32	Roslin3.31	Roslin1.1†			7
HP3.21	Roslin3.33	1*02101						8
HP3.22#	Roslin3.34	2*04701	RoslinSZ.3.1	Roslin (For3Rev1).3.1				2
HP3.23#	Roslin3.35	Roslin (For1Rev2).3.2						2
HP3.24#	Roslin3.36	Roslin3.37	2*01601	Roslin2.27#				2
HP3.25	Roslin3.38	Roslin2.29	2*06001					3
HP3.26†	Roslin3.39	Roslin3.23	Roslin3.40	Roslin3.41†				5
HP3.27#†	Roslin3.42	Roslin3.43†	Roslin (For1Rev2).3.1†					3
HP3.28#	Roslin3.44	Roslin (For3Rev1).3.3						2
HP3.29	Roslin3.45	3*03301N	Roslin3.46					2
HP3.30	Roslin3.5	Roslin3.48	3*03701					8
HP3.31#	Roslin2.1	Roslin3.47	3*00402	Roslin (For3Rev1).3.1				9
HP3.32	Roslin2.5	Roslin2.38	Roslin2.7					4

### Analysis of the MHCI allele repertoire in a *B. indicus* population from Kenya

Having validated the approach, we applied it to analyse the MHCI alleles expressed by individuals from breeds for which there was little or no publically available MHCI sequence data. In a second MiSeq run, cDNA from 100 Boran cattle (a *B. indicus* breed) from Kenya were analysed (Supplementary Data 4b; to accommodate this number of animals, a subset of cDNAs were only amplified with either For1/Rev2 or For3/Rev1 primer sets). These animals included a large half-sib family (70 animals), which based on breeding records were the progeny of a single bull. A total of 29 MHCI haplotypes were identified (Table 3), which apart from BoLA-A12 and BoLA-A15v, were all novel. Together these novel haplotypes included 54 novel alleles and sequences matching 8 known alleles (1*02801, 2*02501, 2*06001, 3*05901, 3*00402, 3*00102, 4*02401, 6*04001). The latter included only two out of the six alleles that had been previously identified from Boran cattle (1*2801 and 3*00102). For 92 animals two haplotypes were defined, for a single animal only one haplotype and no additional alleles were identified (suggesting homozygosity), whilst for each of the remaining 7 animals one haplotype plus a unique combination of alleles were identified (suggesting heterozygosity with an as yet undefined haplotype). These seven undefined haplotypes included 11 putative novel alleles.

### Analysis of the MHCI allele repertoire in a mixed breed *B. indicus/*African *B. tauros* population from Cameroon

In a third MiSeq run, cDNA from 96 cattle from Cameroon was analysed (Supplementary Data 4c)—this included 16 White Fulani, 18 Red Fulani (both Sanga breeds i.e. crosses of *B. indicus* and African *B. tauros* lineages), 6 Goudali (a west African *B. indicus* breed) and 56 animals that were identified as cross-bred (predominantly Red x White Fulani crosses). A total of 44 MHCI haplotypes were identified (Table 3); this included the BoLA-A12 and BoLA-A15v haplotypes, 10 of the haplotypes identified in the Boran animals examined in the 2nd MiSeq run and 32 novel haplotypes. Together, the novel haplotypes included 56 novel alleles and sequences matching 9 known alleles (3*00402|3*05301, 4*06301, 3*03301 N, 3*03701, 2*04701, 3*03201 N, 2*01601, 1*2101 and 2*06001) all of which have previously been defined from European *B. tauros* breeds. Two haplotypes were defined in 85 animals, one haplotype and no additional alleles were identified in 2 animals (suggesting homozygosity), whilst for the remaining 9 animals, one haplotype and a unique combination of alleles were identified (suggesting heterozygosity with an as yet undefined haplotype). These nine putative undefined haplotypes included 16 novel alleles.

Most of the novel allele sequences identified were amplified by both For1/Rev2 and For3/Rev1 primer pairs. However, as described for the 1st MiSeq run above, some low-read frequency alleles were not consistently detected at levels above the cut-off threshold (see Table 3 and Supplementary Data 5–Table 1a). In addition, 4 alleles were amplified by only the For1/Rev2 primer pair and 12 alleles were amplified by only the For3/Rev1 primer pair–indicating that both primers exhibited ‘complete drop out’ of some alleles (see Table 3 and Supplementary Data 5–Table 1b). It was also noted that the sequences of a number of products generated by either For1/Rev2 or For3/Rev1 amplification were ambiguous (i.e. could be derived from multiple alleles; see Supplementary Data 5–Table 2). To accommodate these, modifications to the bioinformatics pipeline with regards to the definition of allele content of the relevant haplotypes and amendments to ambiguous allele designation for the individual PCR reactions were made.

In summary MHCI sequences and at least 1 haplotype could be defined for all 292 animals examined and in total 62 novel haplotypes incorporating 122 novel alleles (as well as 27 putative unassigned classical MHCI alleles and 9 novel non-classical alleles) have been described (Supplementary Data 6). There were a limited number of animals (3 and 5 in the 2nd and 3rd MiSeq runs respectively) where one or two artefacts (i.e. sequences that could not be resolved into the MHCI haplotypes described for that animal) were present (Supplementary Data 4b and c–anomalous variants); however, we consider these rare artefacts to be acceptable given the high throughput and level of resolution of the technique (i.e. acceptance of all sequences <0.2 % of read frequency). Therefore, the data demonstrates that the MiSeq NGS platform developed can be used to rapidly and in detail examine the MHCI allele repertoire of cattle breeds for which there are no currently available sequence data.

### The structure of transcribed bovine MHCI haplotypes

**Fig. 4:**
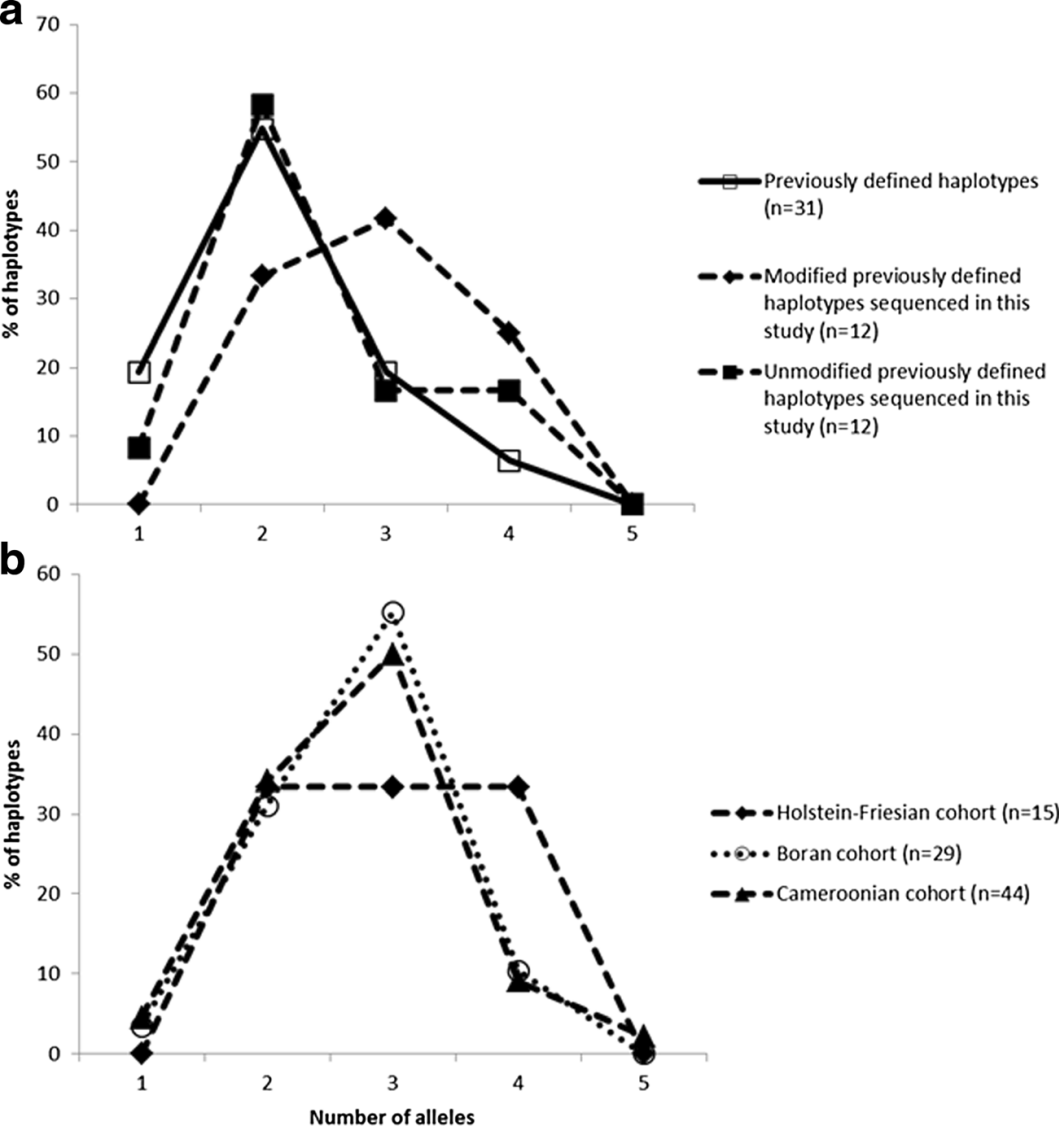
The proportion of bovine MHCI haplotypes expressing different numbers of alleles. **a** The proportion of all previously defined haplotypes (*n* = 31), and the 12 haplotypes identified in this study before (‘unmodified’) and after (‘modified’) inclusion of the novel alleles described in this study, expressing 1, 2, 3 or 4 classical MHCI alleles is shown. **b** The proportion of haplotypes identified in the Holstein-Friesian, Boran and Cameroonian cohorts expressing 1, 2, 3, 4 or 5 classical MHCI alleles is shown

A feature of bovine MHCI is the variation between haplotypes in the number of genes transcribed. Based on previous data most of the 31 defined haplotypes expressed two alleles (Fig. 4a). However, identification of novel alleles in 4 of the 12 previously defined haplotypes sequenced in this study leads to a ‘right-shift’ resulting in a near ‘normal’ distribution centred on three expressed genes. The haplotypes identified in the Boran and Cameroonian cohorts share very similar profiles that also have a normal distribution centred on three expressed genes, whereas the haplotypes in the Holstein-Friesian cohort exhibit a more ‘plateau-like’ profile due to a greater frequency of haplotypes expressing four alleles and a reduction in the frequency of haplotypes expressing three alleles (Fig. 4b).

**Fig. 5:**
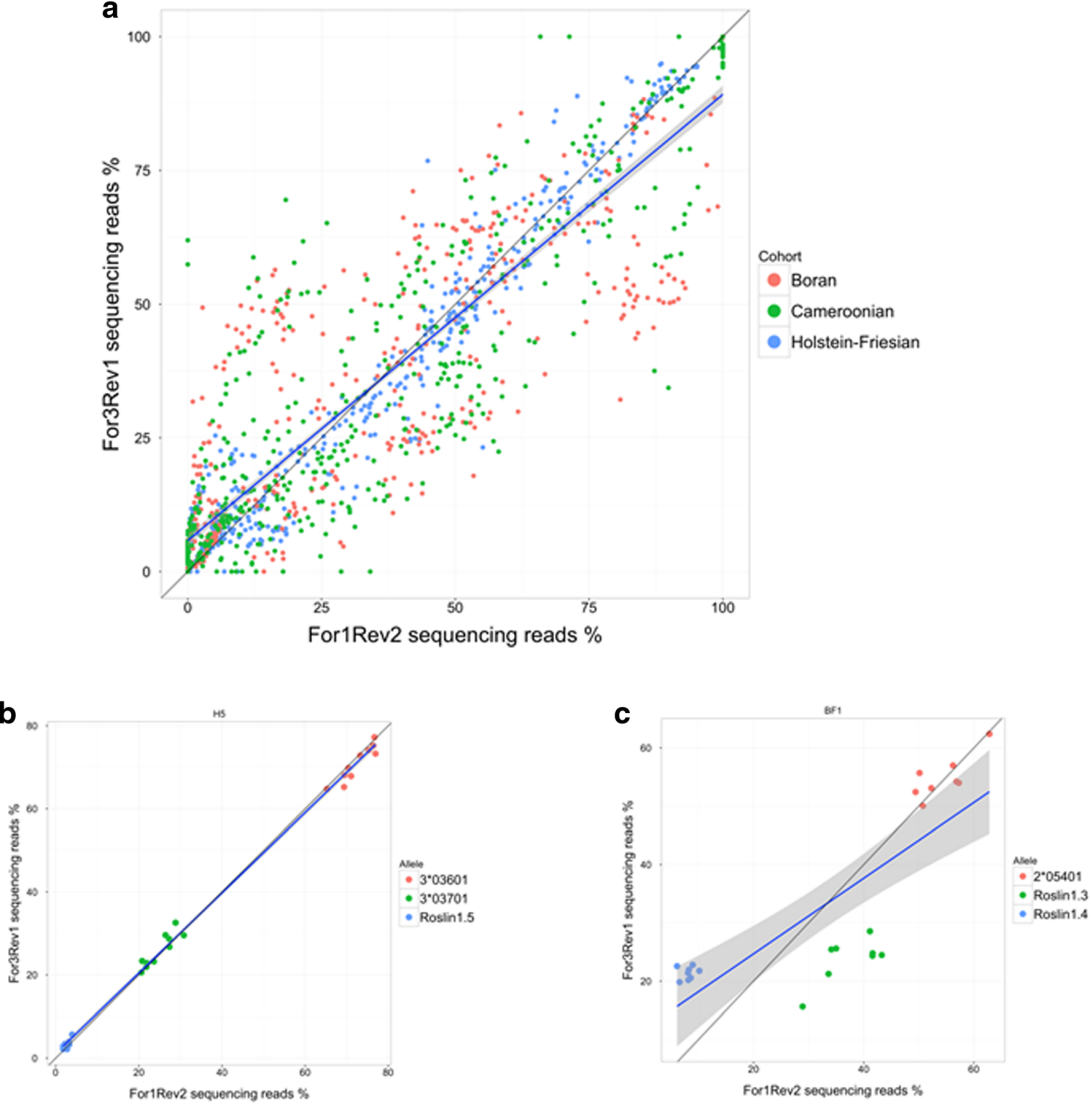
Scatterplot analysis of the read frequency observed with the For1/Rev2 and For3/Rev1 PCR reactions. **a** For each allele in all samples. The line of best fit for the whole dataset is described by the equation *y* = 5.8 + 0.83*x* and the correlation coefficient (*r*) is 0.896. The correlation coefficient values for the Holstein-Friesian, Boran and Cameroonian cohorts separately are 0.98, 0.82 and 0.89 respectively. **b** For the H5 haplotype. Line of best fit is described by the equation *y* = 1.1 + 0.97*x* and *r* = 0.99 and **c** for the BF1 haplotype. Line of best fit is described by the equation *y* = 12 + 0.65*x* and *r* = 0.8.152

**Fig. 6:**
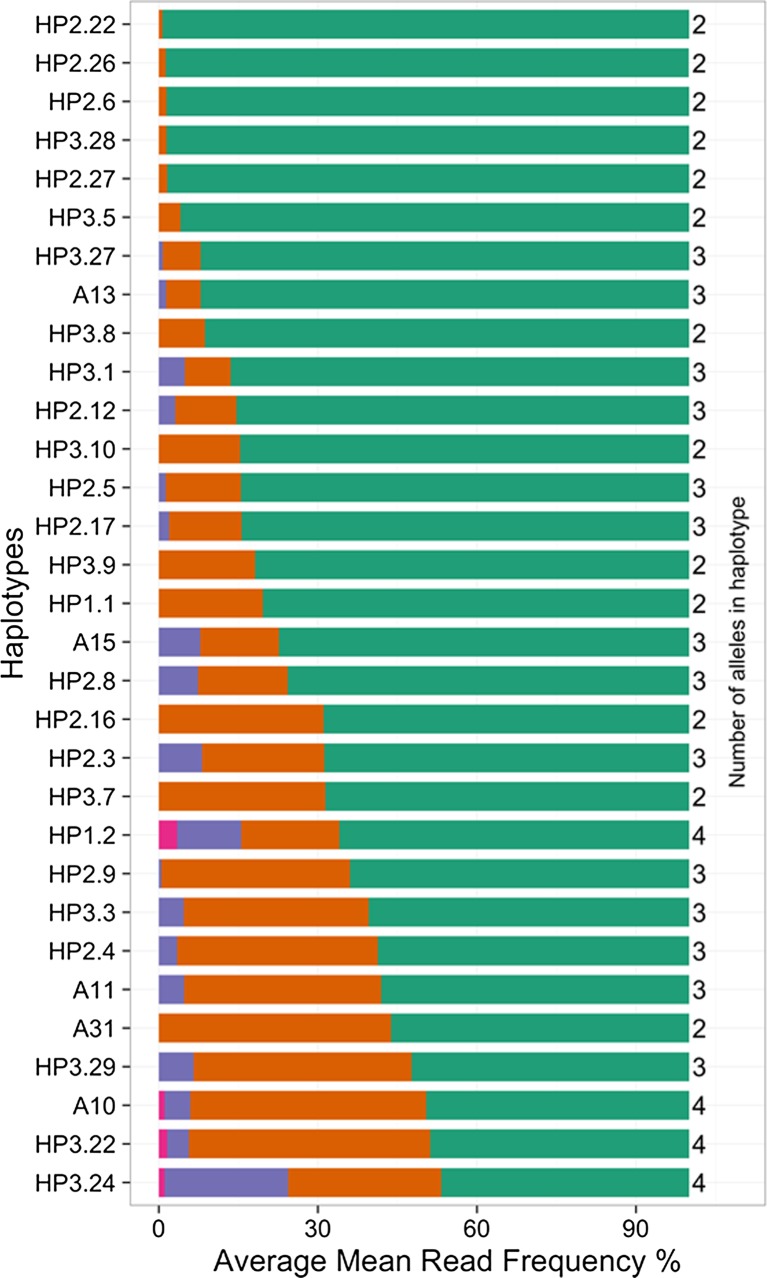
Relative read frequency of alleles in 33 haplotypes. Read frequency data obtained from For1/Rev2 and For3/Rev1 PCR reactions were analysed for all haplotypes individually. It was assumed that if read frequency was not biased by differential PCR amplification efficiency that (i) the relationship between the read frequency obtained by For1Rev2 and For3Rev1 PCR reactions would be described by a simple linear regression model with a slope that would approximate to 1 (0.8–1.3) and (ii) if all alleles were amplified at the same efficiency the correlation coefficient would approximate to 1 (*r* ≥ 0.95) as the data points for all alleles would cluster close to the line of best fit of the linear regression model. Only the data from 33 haplotypes satisfied these criteria. The average read frequency for the alleles (combined For1Rev2 and For3Rev1) in these haplotypes is shown as a stacked bar chart; the first, second, third and fourth highest expressed alleles are represented in *green*, *orange*, *purple* and *red* respectively. The number of each allele in the haplotypes is shown on the *right*

A striking feature of our data was the large variation in read frequency between different alleles in the same haplotype. As read frequency is a product of both starting cDNA copy number and PCR efficiency, we evaluated our data to determine the effect biased PCR amplification may have had. High correlation (*r* = 0.896) of read frequency observed between the total For1/Rev2 and For3/Rev1 primer pair datasets suggested that for much of the data biased PCR efficiency was not a principal determinant of read frequency (Fig. 5a). Equivalent analysis of individual haplotypes (Supplementary Data 7) showed that for some haplotypes, bias due to differential PCR amplification efficiency had a negligible effect on read frequency (e.g. H5 Fig. 5b) whilst for others biased PCR amplification had a demonstrable effect (e.g. BF1–where a bias for Roslin1.4 in For3Rev1 and for Roslin1.3 in For1Rev1 reactions is evident–Fig. 5c). Exclusion of haplotypes where PCR amplification bias was evident left a subset of 33 haplotypes for which read frequency could be inferred to be representative of starting cDNA copy number (Fig. 6). In these haplotypes the read frequency of the most frequent allele ranged over a spectrum from 99.3 to 46.7 %, with the frequency of the 2nd most frequent allele ranging from 45.6 to 0.7 %. Only in HP3.24 was the 3rd allele present at a read frequency of >20 %. Together, these data suggest that the transcribed structure of a bovine MHCI haplotype is determined by a combination of the number of expressed alleles and also the differential transcription levels of those alleles and that both of these parameters can vary markedly between haplotypes.

### Sharing of MHCI alleles and haplotypes between cohorts

**Fig. 7:**
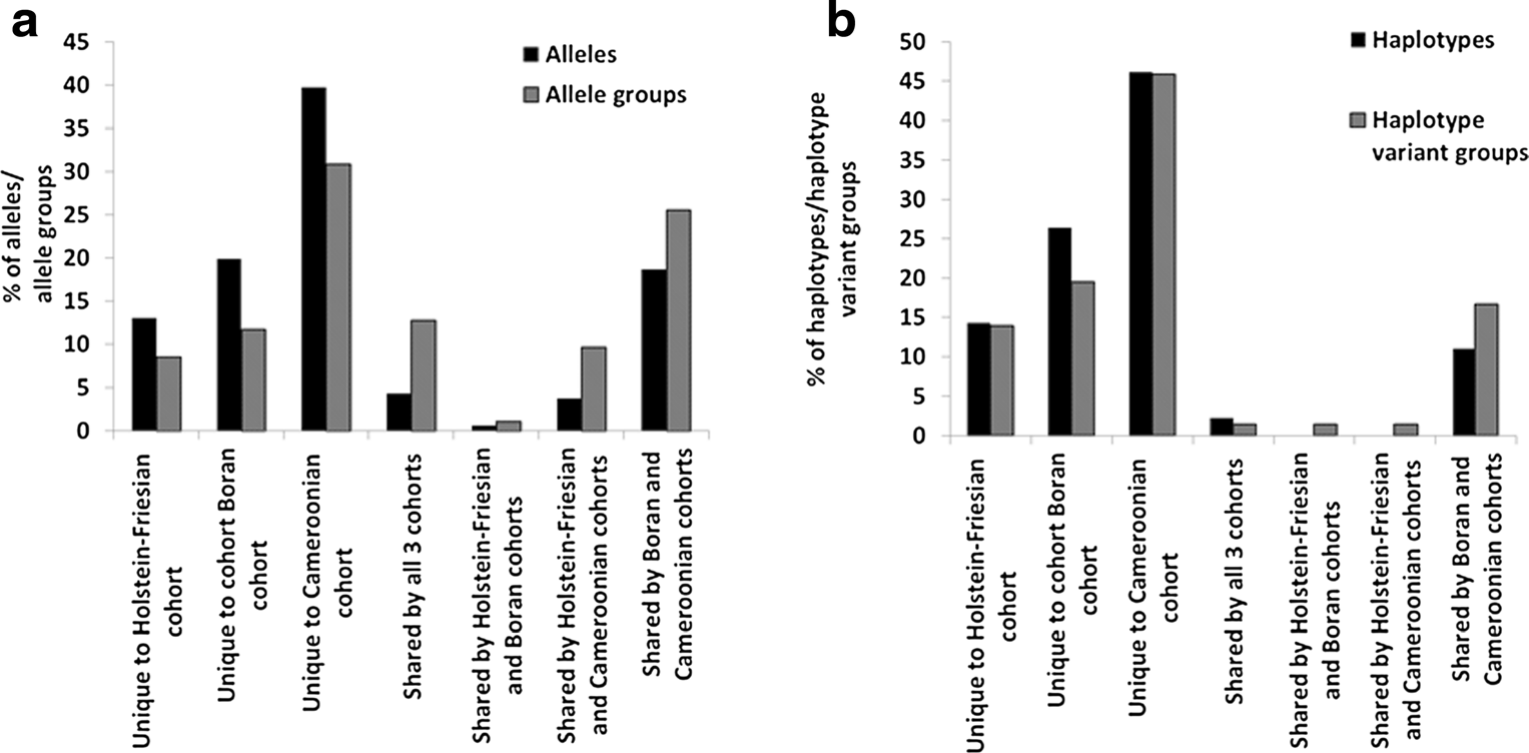
Sharing between cohorts of **a** alleles and allele groups and **b** haplotypes and haplotype variant groups. The percentage for each of these parameters that are unique to single cohorts or are shared between different combinations of them is shown

**Fig. 8:**
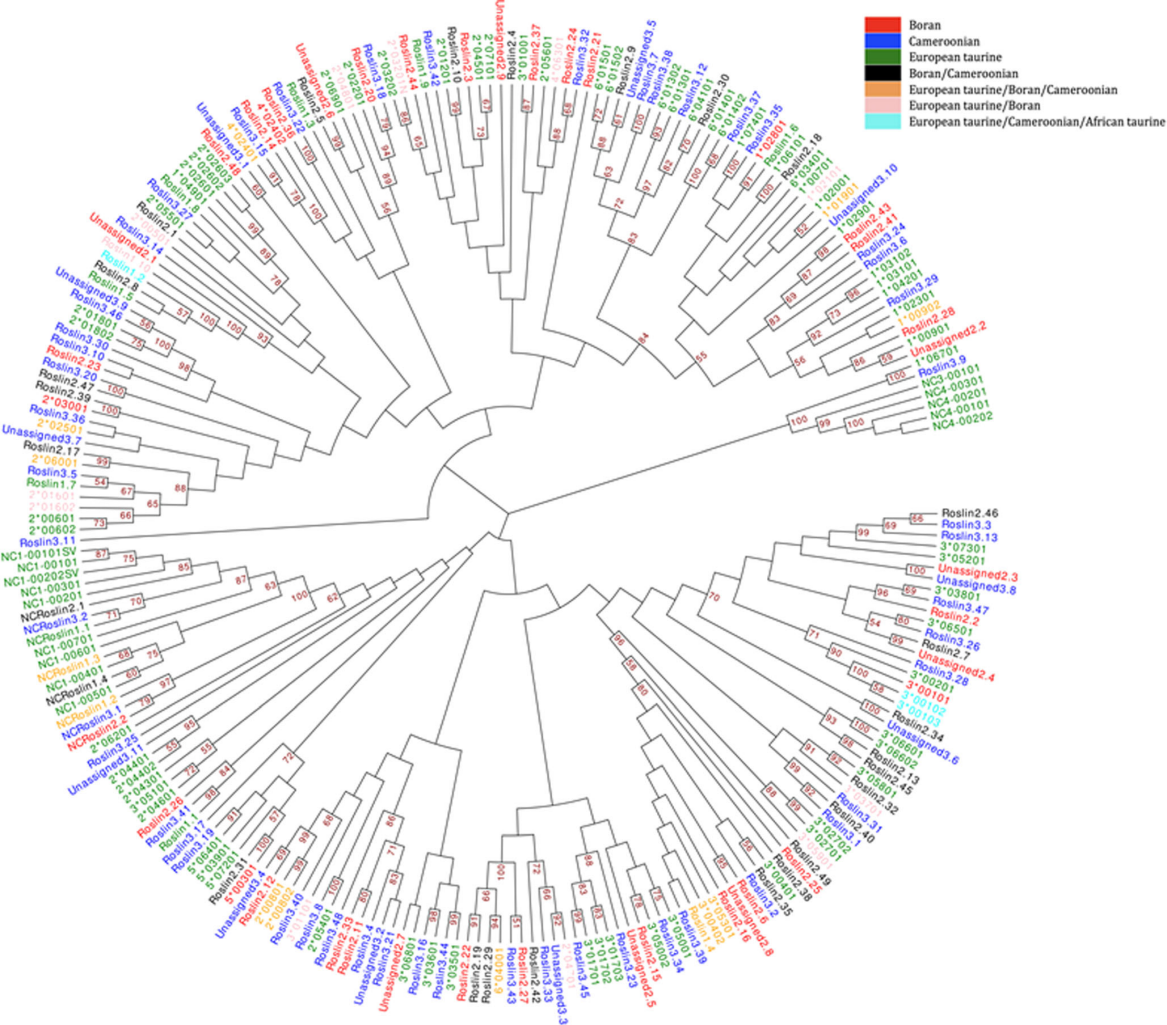
Phylogenetic analysis of bovine MHCI sequences. Neighbour-joining analysis of the amplified 410 bp nucleotide sequence for all novel alleles (excluding those only amplified by one primer pair) and the equivalent sequences from alleles in the IPD-MHC database. Analysis was performed with the MEGA5 software package (Tamura et al. 2011) using the uncorrected nucleotide differences (p-distance) and pairwise deletion to remove gaps in the alignment. The figure was prepared using the Evolview (http://omictools.com/evolview-tool). Sequence labels are coloured as described in the legend to represent in which cohorts/lineages each sequence has been identified

There was limited sharing of alleles and haplotypes between the three cohorts of animals studied–72.7 % of the alleles and 86.8 % of the haplotypes (including the unassigned alleles and haplotypes) identified were unique to single cohorts (Fig. 7). Despite this, phylogenetic analysis of the nucleotide sequences generated in this study and available from the IPD-MHC database shows that sequences from European taurine breeds (including Holstein-Friesians), a *B. indicus* breed (Boran) and the Cameroonian cohort (predominantly Sanga–i.e. African *B. tauros* x *B. indicus* crosses) are extensively intercalated identifying a high level of phylogenetic relatedness (Fig. 8). To investigate this further we examined the pairwise amino acid identity of the 136 residue product encoded by the 410 bp amplified segment (Supplementary Data 8). Amino acid identity ranged from 64 to 100 %, with many clusters of alleles exhibiting >97 % identity (signified by the dark red squares running along the diagonal). These clusters approximate to allele groups (as at >97 % identity these alleles differ by ≤4 amino acid residues across the majority of the α1 and α2 domains), and 42 such putative allele groups included 87 of the novel classical MHCI alleles identified in this study in combination with 50 previously defined MHCI alleles (data not shown). Consideration of allele groups substantially altered the apparent degree of sharing between the cohorts with only 51.1 % of allele groups unique to single cohorts (Fig. 7). Re-examination of the allele composition of haplotypes suggested that 29 haplotypes identified in this study were variants (i.e. haplotypes which express different members of the same allele group(s) in conjunction with identical alleles) of each other and/or previously defined haplotypes (Table 4). However, in contrast to the alleles, consideration of these haplotype variant groups did not substantially increase the apparent level of sharing between the cohorts, with 79.2 % of haplotype groups remaining unique to a single cohort.

**Table 4:** Haplotype variant groups. Eleven haplotype variant groups were identified. The name of haplotypes in each of these groups, the alleles expressed in each haplotype and the cohorts in which the haplotypes were identified is shown. For haplotypes identified in the IPD-MHC database only, the breed in which it has been previously reported is shown (HF = Holstein-Friesian). Alleles that belong to the same allele group are shown in the same column and are represented in *italic*, *underlined script*. For some haplotypes the relative frequency of transcription of the constituent alleles has been inferred from read frequency (Fig. 6)–these haplotypes are marked with an *asterisk* and the percentage of reads for each allele is shown

Haplotype variant group	Haplotype	Alleles				Holstein-Friesian	Boran	Cameroonian	IPD-MHC database
1	A11*	2*01801 (58.1 %)	3*01701 (37.1 %)	3*03301N (4.8 %)		X			X HF
	HP3.29*	*Roslin3.46 (52.4 %)*	*Roslin3.45 (41.1 %)*	3*03301N (6.5 %)				X	
	Unassigned	*Roslin3.45*	*Unassigned3.9*	3*03301N				X	
2	A15v	1*00902	4*02401	2*02501	6*04001	X	X	X	X HF
	A15*	1*00901 (77.3 %)	4*02401 (14.9 %)	2*02501 (7.8 %)		X			X HF
	HP2.13	*Roslin2.28*	4*02401	2*02501	6*04001		X		
	Unassigned	*Unassigned2.2*	4*02401	2*02501			X		
3	KN12	1*02801	4*02402						X Boran
	HP1.1*	*Roslin1.6 (80.4 %)*	4*02401 (19.6 %)			X			
	HP2.23	1*02801	4*02401				X		
4	HP2.1#	*Roslin2.2*	*Roslin (For3Rev1).2.1*	3*00402	Roslin2.1		X		
	HP3.31#	*Roslin3.47*	*Roslin (For3Rev1).3.1*	3*00402	Roslin2.1			X	
5	HP2.3*	*Roslin2.7 (68.8 %)*	*Roslin2.5 (23.0 %)*	Roslin2.6 (8.2 %)			X		
	HP3.32	*Roslin2.7*	*Roslin2.5*	Roslin2.38				X	
	Unassigned	*Unassigned2.4*	*Unassigned2.6*	Roslin2.6			X		
6	HP2.5*	*Roslin2.11 (84.5 %)*	3*00402 (14.2 %)	Roslin2.12 (1.3 %)			X		
	HP2.17*	*Roslin2.33 (84.4 %)*	3*00402 (13.7 %)	Roslin2.12 (1.9 %)			X		
7	HP2.6#*	*Roslin2.13 (98.5 %)*	Roslin (For3Rev1).2.2 (1.5 %)				X	X	
	HP2.26#*	*Roslin2.45 (98.6 %)*	Roslin (For3Rev1).2.2 (1.3 %)				X	X	
8	KN104	5*00301	3*00102						X Boran
	HP2.15	*Roslin2.31*	3*00102				X	X	
9	HP2.18	*Roslin2.34*	*Roslin2.36*	Roslin2.35			X		
	HP3.16	*Roslin2.34*	*Roslin3.22*	Roslin2.35				X	
10	A25	1*02901	2*03001						X Boran
	HP2.21	*Roslin2.41*	*Roslin2.39*	*Roslin2.40*			X		
	HP2.24	*Roslin2.43*	*Roslin2.39*	*Roslin2.40*			X		
	HP2.25	*Roslin2.43*	*Roslin2.39*	*Roslin2.44*			X		
	HP3.3*	*Roslin3.6 (60.4 %)*	*Roslin2.39 (34.9 %)*	*Roslin2.40 (4.7 %)*				X	
	HP3.17	*Roslin3.24*	*Roslin2.39*	*Roslin2.40*				X	
11	HP3.2	*Roslin3.4*	Roslin3.5	3*03701				X	
	HP3.15	*Roslin3.21*	Roslin3.5	3*03701				X	
	HP3.30	*Roslin3.48*	Roslin3.5	3*03701				X	

## Discussion

In this study we describe the development, validation and application of a novel NGS approach to rapidly characterise the repertoire of expressed classical MHCI genes in cattle. This approach has been successful in permitting sequencing of MHCI alleles not only from animals of a well-characterised European *B. tauros* breed but also from African *B. indicus* and Sanga (i.e. African *B. tauros* × *B. indicus*) breeds. Analyses of sequences from 292 animals identified over 140 novel MHCI alleles and defined 62 novel haplotypes.

Platforms that utilise PCR amplification of MHC genes prior to NGS analysis have various inherent limitations, which include the introduction of sequence artefacts during PCR/sequencing, allele dropout, ambiguity between closely related alleles and the need for correct phasing of sequences. Steps introduced into the study design aimed to, as far as possible, mitigate these limitations. The low number of anomalous sequences detected (a total of 14 in the whole study) suggests that the series of filtering steps in the bioinformatic pipeline to remove chimaeras and other errors were largely sufficient to remove artefacts from the datasets. Minimisation of allele dropout due to mismatches in primer annealing sites was the principal reason we elected to use two independent PCRs for amplification. This is a major issue and can be difficult to resolve for PCR-based approaches (Hosomichi et al. 2015), especially for species such as cattle for which it is not possible to predict how many MHCI sequences an individual will express due to variation in the allele complement between haplotypes. Of the 139 novel putative classical MHCI alleles identified in this study, 16 (11.5 %) were amplified by only one of the primer pairs, demonstrating the utility of such an approach. We believe the strategy of combining two independent PCRs, and a low-threshold cut-off (0.2 %) has been effective in minimising the number of alleles missed; this is supported by the fact that the number of alleles/haplotype identified in this study (mean = 2.79) is higher than that for previously defined bovine MHCI haplotypes (mean = 2.13) and that we identified several new alleles on haplotypes that have been intensively studied over many decades (e.g. A10). Low read frequency for a substantial number of novel alleles, including the majority of those identified in previously defined haplotypes, suggests that these may represent ‘suppressed’ alleles or non-functional alleles that can be exceptionally difficult to detect using lower resolution Sanger sequencing (Lange et al. 2014). However, it is not possible to state with certainty that all of the alleles present in the animals studied have been successfully identified.

To minimise generating ambiguous sequences that could match multiple alleles our PCR, amplicons were designed to incorporate most of the hypervariable exon 2/3 region. Only five pairs of previously defined alleles in the IPD-MHC database failed to be discriminated by the combined For1/Rev2 and For3/Rev1 amplicon sequences. As was to be expected, as novel alleles were identified the number of ambiguous sequences identified increased (Erlich 2012); 12 more incidences of primer-pair-specific ambiguity were recognised by comparing For1Rev2 and For3Rev1 sequences amplified from the same cDNA. By definition it was not possible to detect and therefore quantify how many sequences were ambiguous for both For1Rev2 and For3/Rev1 amplicons although amplification of Roslin 2.27 by both For1Rev2 and For3Rev1 primer pairs in HP2.12, but only by For3/Rev1 primer pair in HP3.24 suggests that this sequence may be ambiguous and represent multiple alleles. Improved resolution to remove ambiguity of the coding sequence of bovine MHCI alleles (equivalent of the 4- or 6-digit resolution in HLA) will require full-length sequencing of the coding domains, which was beyond the scope of the present study.

The use of cDNA as the starting template allowed the amplification of (most of) exons 2 and 3 together as a single PCR product, avoiding the need for phasing that would have resulted from amplifying exon 2 and exon 3 individually from genomic DNA. The next order of phasing–combining alleles together to form haplotypes–was facilitated in this study by either selecting (i) a breed in which most of the common haplotypes had already been defined (Holstein-Friesian cohort), (ii) a group in which most of the animals were sired by a single bull (Boran cohort) or (iii) selecting related animals (Cameroonian cohort)–so guaranteeing that a proportion of the haplotypes would be present multiple times in the cohorts and so recurrent patterns of alleles could be observed. This strategy was largely successful with only 16 haplotypes that could not be confirmed as they were only identified once.

We used the data generated to examine two aspects of MHCI biology. The first of these was the relative level of expression of different alleles within haplotypes. Our data demonstrates that due to biased PCR amplification efficiency read frequency data from single PCR reactions cannot be reliably used to define initial cDNA copy number for alleles (e.g. Fig. 6c) but that comparison of data from two independent PCR reactions can enable PCR bias to be identified and removed. This allowed us to identify a subset of 33 haplotypes from which relative allele expression level could be inferred. In many of these haplotypes (18/33), irrespective of the expression of either two or three alleles, there was a single highly dominant allele that represented >75 % of the reads. For the other haplotypes the dominance of the most frequent allele was less pronounced and in a small number of haplotypes (e.g. A31 and HP3.29) the two most frequent alleles were co-dominant (or nearly so). Although 17/33 of the haplotypes examined expressed either three or four alleles, in all but two (HP3.24 and HP1.2) the frequency of the third most common allele represented <10 % of the reads. Consequently for most haplotypes >90 % of read frequency was accounted for by only the one or two of the most frequent alleles. The data suggest there is marked variation between bovine MHCI haplotypes in their ‘transcribed structure’ and that differential transcription levels, as well as the number of alleles expressed, is a key determinant of this. In humans HLA-A and HLA-B alleles are expressed at approximately equivalent levels and at about ∼5 to 15× higher levels that HLA-C (Apps et al. 2015; Snary et al. 1977), and there is evidence that variation in levels of expression of alleles from these loci can have significant clinical implications–for example, higher HLA-C expression is associated with improved protection from HIV (Apps et al. 2013). Unfortunately as our sequences did not include the 3′ end of the MHCI sequences used to assign alleles to distinct loci (Hammond et al. 2012), we had insufficient data to examine if the expression level was correlated to putative loci defined in the bovine MHCI system. Furthermore, we were unable to confirm if any of the alleles detected at low frequency carried ‘indels’ or SNPs that would be predicted to render them non-functional. Regardless of this, the fundamental questions as to why is there is such disparity between the expression profile of different bovine MHCI haplotypes, and what the possible biological consequences of this are, remain. Recent work in humans and chickens has demonstrated that the breadth of peptide presentation and level of cell surface expression of MHCI molecules are inversely correlated (Chappell et al. 2015). The authors propose that this inverse relationship is a fundamental feature of MHCI, which would have obvious ramifications for how we consider the potential functional implications of the range of transcribed bovine MHCI haplotype structures has. Future work needs to use alternative methods to confirm the differential levels of allele expression at both the mRNA and protein levels (Apps et al. 2015) and explore the potential functional consequences for CD8+ T cell and NK cell responses.

The second issue we addressed was the sharing of MHCI sequences and loci between the three cohorts of animals which represented European *B. tauros*, African *B. indicus* and Sanga (African *B. tauros* × *B. indicus)* lineages. The data are, to the authors’ knowledge, the first large dataset of MHC sequences generated from non-European *B. tauros* breeds. The extensive phylogenetic intermingling of sequences from the different cohorts with representatives from all three lineages present in most clades suggests that the exon 2/3 sequences in all cattle are derived from a common set of ancestral MHCI alleles. However, most of the identified sequences (72.7 %) were unique to individual cohorts as were the defined haplotypes (86.8 %). This lack of overlap may be explained by the undoubtedly incomplete sampling of the MHCI diversity within the relevant breeds/lineages but also by allele sequence diversification following lineage divergence. Substantially higher levels of sharing among the cohorts of allele groups (only 51.1 % were identified in a single cohort) is consistent with the latter. Notably, in variant haplotypes where allele expression level was inferred (marked with * in Table 4), it was the higher expressed alleles that varied between haplotypes–indicating that such alleles may be subjected to greater selection for sequence divergence. The disparity between the effect on the observed level of sharing between the cohorts when considering allele groups (unique to a single cohort decreased from 72.7 to 51.1 %) and haplotype variant groups (decrease from 86.8 to 79.2 %) suggests that distinct recombination events in different lineages has resulted in the formation of unique haplotypes (for example the expression of Roslin1.4 in BF1 and HP3.12, apparently unrelated haplotypes in the Holstein-Friesian and Cameroonian cohorts respectively). Together, the data implies that sequence divergence and the generation of different permutations of alleles by recombination are both contributing to the evolutionary diversification of bovine MHCI haplotypes. However, several caveats need to be considered in interpreting this preliminary data. Firstly, the genetic lineage of African breeds remains controversial and is complicated by introgression of genes from several *B. indicus* influxes from the Middle East and more recently European *B. tauros* (Bradley et al. 1996; Decker et al. 2014; Troy et al. 2001), and as seen in our Cameroonian cohort, frequent cross-breeding is not uncommon and contributes to genetic admixture. Secondly, there is evidence that bovine MHCI exon 2 and exon 3 may undergo frequent gene conversion events as a consequence of ‘break-points’ in the flanking introns (Schwartz and Hammond 2015) so complicating the use of these exons to examine the relatedness of alleles (as well as suggesting a further mechanism used to diversify the bovine MHCI repertoire). Further studies generating larger datasets incorporating more breeds (including samples from pure Asian *B. indicus* and African *B. tauros* animals), and ideally sequencing the full coding regions of the MHCI alleles, will be needed to more comprehensively evaluate the diversity of MHCI genes in the global cattle population and assess the forces that have shaped its evolution.

The laborious, time-consuming and expensive nature of bovine MHC typing using previous techniques has placed large constraints on the capacity of the bovine research community to perform large-scale analysis of this key immunological gene family. The laboratory time and effort required to achieve large-scale typing using the MiSeq platform described herein is comparatively small, and trivial modification could reduce the costs of sequencing an individual to ∼ £2.50. The capacity of the NGS/bioinformatic pipeline developed in this study to accurately, rapidly and cheaply identify the MHCI haplotypes of cattle makes large-scale MHC typing studies feasible. Such studies will find applications in the analysis of the evolutionary biology of the bovine MHC system, associations between bovine MHC and disease resistance/susceptibly and also permit a comprehensive assessment of the diversity of bovine MHCI and the implications this may have of future development of vaccines aimed at inducing CD8+ T cell responses.

## Electronic Supplementary Materials

Below is the link to the electronic supplementary material.

Supplementary Data 1 Primers for universal bovine MHCI allele amplification. Sequence details of the primers used in the study. (DOCX 19 kb)

Supplementary Data 2 Details of in silico evaluation of the universal bovine MHCI allele primers. (A) Notes on the nomenclature of bovine MHCI. Tables summarising (B) MHCI alleles in the IPD-MHC database exhibiting mismatches within primer annealing sites. (C) MHCI alleles in the IPD-MHC database exhibiting mismatches with the primer pairs used for PCR reactions and that cannot be discriminated following sequencing of the generated amplicons. (DOCX 16 kb)

Supplementary Data 3 Output from bioinformatics analysis. (A) A file describing the output from the bioinformatic pipeline. (B) The excel spreadsheet component of the initial output from the pipeline from the first MiSeq run is provided as an example. (DOCX 18 kb) (XLSX 79 kb)

Supplementary Data 4 Summary of results from MiSeq sequencing and bioinformatic analysis. For (A) Holstein-Friesian cohort analysed in the 1st MiSeq run (B) Boran cohort analysed in the 2nd MiSeq run and (C) Cameroonian cohort analysed in the 3rd MiSeq run. (XLSX 68 kb) (XLSX 65 kb) (XLSX 169 kb)

Supplementary Data 5 Tables summarising allele dropout and ambiguity. Table 1a List of haplotypes containing alleles not consistently present above the 0.2 % read frequency cut-off threshold–partial allele dropout. Table 1b List of haplotypes containing alleles which were only detected following amplification with either the For1Rev2 or For3Rev1 primer pairs–complete allele dropout. Table 2 Details of novel MHCI alleles that cannot be discriminated following sequencing of the amplicons generated by the For1/Rev2 and For3/Rev1 primer pairs. (DOCX 21 kb)

Supplementary Data 6 Fasta files for all novel sequences identified in this study. The nucleotide sequences for all novel putative alleles described in this study are provided in fasta format. (FASTA 64 kb)

Supplementary Data 7 Scatterplot analysis of the read frequency observed with the For1/Rev2 and For3/Rev1 PCR reactions for each individual haplotype identified in (A) the Holstein-Friesian cohort, (B) the Boran cohort and (C) the Cameroonian cohort. For each haplotype the read frequency for each allele from each individual for both For1/Rev2 and For3/Rev1 PCR reactions is shown. The line of best is represented and the equation describing this, the coefficient of correlation (r) and the correlation of determination (r2) are shown. (PDF 47 kb) (PDF 70 kb) (PDF 102 kb)

Supplementary Data 8 Heat map showing the percentage pairwise identity between the amino acid sequences encoded by the amplified 410 bp nucleotide sequence. Pairwise amino acid identity was calculated with the EMBOSS Needle programme (http://www.ebi.ac.uk/Tools/psa/emboss_needle). The colour key used to create the heat map with the corresponding percentage identity is shown in the legend in the top left corner. (PDF 497 kb)
