# Supplementary material for: Rapid identification of bovine MHCI haplotypes in genetically divergent cattle populations using next-generation sequencing
Source: Immunogenetics. 2016 Aug 11;68(10):765–81. doi: 10.1007/s00251-016-0945-7 (PMC5056950; doi:10.1007/s00251-016-0945-7)
Supplement: Supplementary file 1 — Primers for universal bovine MHCI allele amplification. Sequence details of the primers used in the study. (DOCX 19 kb) [file 251_2016_945_MOESM1_ESM.docx]

**Supplementary data 1 - Primers for universal bovine MHCI allele amplification**

Sequences of the sets of TCMHCfor1 and TCMHCfor3 primers with the Illumina D501-D508 adaptors and TCMHCrev1 and TCMHCrev2 primers with the Illumina D701-712 used for PCR amplification. The bovine MHCI-specific portion of the primers, the sequences of the MID tags and the Illumina adaptor/ligation sequences are shown in green, red and black script respectively.

| Primer name |  | |
| --- | --- | --- |
| TCMHCfor1-D501 | AAT GAT ACG GCG ACC ACC GAG ATC TAC AC TATAGCCT ACA CTC TTT CCC TAC ACG ACG CTC TTC CGA TCT GTY GGC TAY GTG GAC GAC | |
| TCMHCfor1-D502 | AAT GAT ACG GCG ACC ACC GAG ATC TAC AC ATAGAGGC ACA CTC TTT CCC TAC ACG ACG CTC TTC CGA TCT GTY GGC TAY GTG GAC GAC | |
| TCMHCfor1-D503 | AAT GAT ACG GCG ACC ACC GAG ATC TAC AC CCTATCCT ACA CTC TTT CCC TAC ACG ACG CTC TTC CGA TCT GTY GGC TAY GTG GAC GAC | |
| TCMHCfor1-D504 | AAT GAT ACG GCG ACC ACC GAG ATC TAC AC GGCTCTGA ACA CTC TTT CCC TAC ACG ACG CTC TTC CGA TCT GTY GGC TAY GTG GAC GAC | |
| TCMHCfor1-D505 | AAT GAT ACG GCG ACC ACC GAG ATC TAC AC AGGCGAAG ACA CTC TTT CCC TAC ACG ACG CTC TTC CGA TCT GTY GGC TAY GTG GAC GAC | |
| TCMHCfor1-D506 | AAT GAT ACG GCG ACC ACC GAG ATC TAC AC TAATCTTA ACA CTC TTT CCC TAC ACG ACG CTC TTC CGA TCT GTY GGC TAY GTG GAC GAC | |
| TCMHCfor1-D507 | AAT GAT ACG GCG ACC ACC GAG ATC TAC AC CAGGACGT ACA CTC TTT CCC TAC ACG ACG CTC TTC CGA TCT GTY GGC TAY GTG GAC GAC | |
| TCMHCfor1-D508 | AAT GAT ACG GCG ACC ACC GAG ATC TAC AC GTACTGAC ACA CTC TTT CCC TAC ACG ACG CTC TTC CGA TCT GTY GGC TAY GTG GAC GAC | |
|  | |  |
| TCMHCfor3-D501 | AAT GAT ACG GCG ACC ACC GAG ATC TAC AC TATAGCCT ACA CTC TTT CCC TAC ACG ACG CTC TTC CGA TCT GGG CYV GAG TAT TGG GA | |
| TCMHCfor3-D502 | AAT GAT ACG GCG ACC ACC GAG ATC TAC AC ATAGAGGC ACA CTC TTT CCC TAC ACG ACG CTC TTC CGA TCT GGG CYV GAG TAT TGG GA | |
| TCMHCfor3-D503 | AAT GAT ACG GCG ACC ACC GAG ATC TAC AC CCTATCCT ACA CTC TTT CCC TAC ACG ACG CTC TTC CGA TCT GGG CYV GAG TAT TGG GA | |
| TCMHCfor3-D504 | AAT GAT ACG GCG ACC ACC GAG ATC TAC AC GGCTCTGA ACA CTC TTT CCC TAC ACG ACG CTC TTC CGA TCT GGG CYV GAG TAT TGG GA | |
| TCMHCfor3-D505 | AAT GAT ACG GCG ACC ACC GAG ATC TAC AC AGGCGAAG ACA CTC TTT CCC TAC ACG ACG CTC TTC CGA TCT GGG CYV GAG TAT TGG GA | |
| TCMHCfor3-D506 | AAT GAT ACG GCG ACC ACC GAG ATC TAC AC TAATCTTA ACA CTC TTT CCC TAC ACG ACG CTC TTC CGA TCT GGG CYV GAG TAT TGG GA | |
| TCMHCfor3-D507 | AAT GAT ACG GCG ACC ACC GAG ATC TAC AC CAGGACGT ACA CTC TTT CCC TAC ACG ACG CTC TTC CGA TCT GGG CYV GAG TAT TGG GA | |
| TCMHCfor3-D508 | AAT GAT ACG GCG ACC ACC GAG ATC TAC AC GTACTGAC ACA CTC TTT CCC TAC ACG ACG CTC TTC CGA TCT GGG CYV GAG TAT TGG GA | |
| TCMHCrev1-D701 | CAA GCA GAA GAC GGC ATA CGA GAT CGAGTAAT GTG ACT GGA GTT CAG ACG TGT GCT CTT CCG ATC T CTC CAG GTR TCT GSG GAG C | |
| TCMHCrev1-D702 | CAA GCA GAA GAC GGC ATA CGA GAT TCTCCGGA GTG ACT GGA GTT CAG ACG TGT GCT CTT CCG ATC T CTC CAG GTR TCT GSG GAG C | |
| TCMHCrev1-D703 | CAA GCA GAA GAC GGC ATA CGA GAT AATGAGCG GTG ACT GGA GTT CAG ACG TGT GCT CTT CCG ATC T CTC CAG GTR TCT GSG GAG C | |
| TCMHCrev1-D704 | CAA GCA GAA GAC GGC ATA CGA GAT GGAATCTC GTG ACT GGA GTT CAG ACG TGT GCT CTT CCG ATC T CTC CAG GTR TCT GSG GAG C | |
| TCMHCrev1-D705 | CAA GCA GAA GAC GGC ATA CGA GAT TTCTGAAT GTG ACT GGA GTT CAG ACG TGT GCT CTT CCG ATC T CTC CAG GTR TCT GSG GAG C | |
| TCMHCrev1-D706 | CAA GCA GAA GAC GGC ATA CGA GAT ACGAATTC GTG ACT GGA GTT CAG ACG TGT GCT CTT CCG ATC T CTC CAG GTR TCT GSG GAG C | |
| TCMHCrev1-D707 | CAA GCA GAA GAC GGC ATA CGA GAT AGCTTCAG GTG ACT GGA GTT CAG ACG TGT GCT CTT CCG ATC T CTC CAG GTR TCT GSG GAG C | |
| TCMHCrev1-D708 | CAA GCA GAA GAC GGC ATA CGA GAT GCGCATTA GTG ACT GGA GTT CAG ACG TGT GCT CTT CCG ATC T CTC CAG GTR TCT GSG GAG C | |
| TCMHCrev1-D709 | CAA GCA GAA GAC GGC ATA CGA GAT CATAGCCG GTG ACT GGA GTT CAG ACG TGT GCT CTT CCG ATC T CTC CAG GTR TCT GSG GAG C | |
| TCMHCrev1-D710 | CAA GCA GAA GAC GGC ATA CGA GAT TTCGCGGA GTG ACT GGA GTT CAG ACG TGT GCT CTT CCG ATC T CTC CAG GTR TCT GSG GAG C | |
| TCMHCrev1-D711 | CAA GCA GAA GAC GGC ATA CGA GAT GCGCGAGA GTG ACT GGA GTT CAG ACG TGT GCT CTT CCG ATC T CTC CAG GTR TCT GSG GAG C | |
| TCMHCrev1-D712 | CAA GCA GAA GAC GGC ATA CGA GAT CTATCGCT GTG ACT GGA GTT CAG ACG TGT GCT CTT CCG ATC T CTC CAG GTR TCT GSG GAG C | |
|  |  | |
| TCMHCrev2-D701 | CAA GCA GAA GAC GGC ATA CGA GAT CGAGTAAT GTG ACT GGA GTT CAG ACG TGT GCT CTT CCG ATC T GGC CCT CSA SGT AGT TCC T | |
| TCMHCrev2-D702 | CAA GCA GAA GAC GGC ATA CGA GAT TCTCCGGA GTG ACT GGA GTT CAG ACG TGT GCT CTT CCG ATC T GGC CCT CSA SGT AGT TCC T | |
| TCMHCrev2-D703 | CAA GCA GAA GAC GGC ATA CGA GAT AATGAGCG GTG ACT GGA GTT CAG ACG TGT GCT CTT CCG ATC T GGC CCT CSA SGT AGT TCC T | |
| TCMHCrev2-D704 | CAA GCA GAA GAC GGC ATA CGA GAT GGAATCTC GTG ACT GGA GTT CAG ACG TGT GCT CTT CCG ATC T GGC CCT CSA SGT AGT TCC T | |
| TCMHCrev2-D705 | CAA GCA GAA GAC GGC ATA CGA GAT TTCTGAAT GTG ACT GGA GTT CAG ACG TGT GCT CTT CCG ATC T GGC CCT CSA SGT AGT TCC T | |
| TCMHCrev2-D706 | CAA GCA GAA GAC GGC ATA CGA GAT ACGAATTC GTG ACT GGA GTT CAG ACG TGT GCT CTT CCG ATC T GGC CCT CSA SGT AGT TCC T | |
| TCMHCrev2-D707 | CAA GCA GAA GAC GGC ATA CGA GAT AGCTTCAG GTG ACT GGA GTT CAG ACG TGT GCT CTT CCG ATC T GGC CCT CSA SGT AGT TCC T | |
| TCMHCrev2-D708 | CAA GCA GAA GAC GGC ATA CGA GAT GCGCATTA GTG ACT GGA GTT CAG ACG TGT GCT CTT CCG ATC T GGC CCT CSA SGT AGT TCC T | |
| TCMHCrev2-D709 | CAA GCA GAA GAC GGC ATA CGA GAT CATAGCCG GTG ACT GGA GTT CAG ACG TGT GCT CTT CCG ATC T GGC CCT CSA SGT AGT TCC T | |
| TCMHCrev2-D710 | CAA GCA GAA GAC GGC ATA CGA GAT TTCGCGGA GTG ACT GGA GTT CAG ACG TGT GCT CTT CCG ATC T GGC CCT CSA SGT AGT TCC T | |
| TCMHCrev2-D711 | CAA GCA GAA GAC GGC ATA CGA GAT GCGCGAGA GTG ACT GGA GTT CAG ACG TGT GCT CTT CCG ATC T GGC CCT CSA SGT AGT TCC T | |
| TCMHCrev2-D712 | CAA GCA GAA GAC GGC ATA CGA GAT CTATCGCT GTG ACT GGA GTT CAG ACG TGT GCT CTT CCG ATC T GGC CCT CSA SGT AGT TCC T | |
