# Supplementary material for: Rapid identification of bovine MHCI haplotypes in genetically divergent cattle populations using next-generation sequencing
Source: Immunogenetics. 2016 Aug 11;68(10):765–81. doi: 10.1007/s00251-016-0945-7 (PMC5056950; doi:10.1007/s00251-016-0945-7)
Supplement: Supplementary file 2 — Details of in silico evaluation of the universal bovine MHCI allele primers. (A) Notes on the nomenclature of bovine MHCI. Tables summarising (B) MHCI alleles in the IPD-MHC database exhibiting mismatches within primer annealing sites. (C) MHCI alleles in the IPD-MHC database exhibiting mismatches with the primer pairs used for PCR reactions and that cannot be discriminated following sequencing of the generated amplicons. (DOCX 16 kb) [file 251_2016_945_MOESM2_ESM.docx]

**Supplementary data 2 – Details of *in silico* evaluation of the universal bovine MHCI allele primers**

**A – Notes on nomenclature of bovine MHCI**

Cattle MHCI nomenclature is based on the HLA nomenclature system. Allele names are based on amino acid sequence and consist of a number signifying the putative locus, followed by an asterisk and then 5 to 9 digits. The first 3 digits indicate the allele 'group', the second 2 indicate coding change, the next 2 indicate non-coding change, and the last 2 indicate promoter/intron change - the last 4 digits are therefore rarely used. Allele groups consist of sequences containing a maximum of four amino acid changes within the alpha 1 and 2 domains, plus up to four amino acid changes in any other parts of the coding sequence. (Adapted from the Rules for naming new alleles on the BoLA page of the IPD-MHC database).

**B - MHCI alleles in the IPD-MHC database exhibiting mis-matches within primer annealing sites.**

| Primer Name | Annealing position* | Primer sequence | MHCI alleles with mis-matches to primer  (within the first 18bp) | | | |
| --- | --- | --- | --- | --- | --- | --- |
|  |  |  | MHCI name | Position of mismatch | Nucleotide | |
|  |  |  |  |  | Primer | MHCI allele |
| TCMHCfor1 | 136-153 | GTY GGC TAY GTG GAC GAC | 3*01001 | 3 | G | A |
|  |  |  | 1*06701 | 7 | G | C |
| TCMHCfor3 | 229-245 | GGG CYV GAG TAT TGG GA | 1*06701 | 9 | G | A |
|  |  |  | 1*06701 | 15 | G | C |
| TCMHCrev1 | 564-582 | CTC CAG GTR TCT GSG GAG C |  |  |  |  |
| TCMHCrev2 | 532-550 | GGC CCT CSA SGT AGT TCC T | 3*06801 | 2 | G | C |
|  |  |  | 6*04001 | 6 | C | G |

* positions of primer on CDs sequence of BoLA-3*00401

**C - MHCI alleles in the IPD-MHC database exhibiting mis-matches with the primer pairs used for PCR reactions and that can’t be discriminated following sequencing of the generated amplicons.**

|  | Primer pair | |
| --- | --- | --- |
|  | TCMHCfor1/TCMHCrev2 | TCMHCfor3/TCMHCrev1 |
| Size of product (bp) | 348 | 318 |
| MHCI alleles with mis-match to primers  (within the first 18bp) | 3*01001, 1*6701, 3*06801, 6*04001 | 1*06701 |
| MHCI alleles not discriminated by sequencing of amplicons (ambiguous alleles) | 3*00402/3*05301 | 3*00402/3*05301 |
|  | 3*00102/3*00103 | 3*00102/3*00103 |
|  | 2*00601/2*00602 | 2*00601/2*00602 |
|  | 2*01801/2*01802 | 2*01801/2*01802 |
|  | 2*02601/2*02603 | 2*02601/2*02603 |
|  | 2*00801/2*00802 |  |
|  | 2*01601/2*001602 |  |
|  |  | 2*03201N/2*03202N |
