# Supplementary material for: Rapid identification of bovine MHCI haplotypes in genetically divergent cattle populations using next-generation sequencing
Source: Immunogenetics. 2016 Aug 11;68(10):765–81. doi: 10.1007/s00251-016-0945-7 (PMC5056950; doi:10.1007/s00251-016-0945-7)
Supplement: Supplementary file 3 — Output from bioinformatics analysis. (A) A file describing the output from the bioinformatic pipeline. (B) The excel spreadsheet component of the initial output from the pipeline from the first MiSeq run is provided as an example. (DOCX 18 kb) [file 251_2016_945_MOESM3_ESM.docx]

**Supplementary Data 3 – Output from bioinformatics analysis**

The output of the analysis is given as an excel workbook containing 4 spreadsheets and a set of fasta files.

**Spreadsheet 1 – ‘Stats’**

Provides a comprehensive statistical overview of the sequencing parameters and quantitative details of the MHCI analysis pipeline for each sample.

| Column | Parameter | Notes |
| --- | --- | --- |
| A | Well | The same MID tags are used for For1/Rev2 and For3/Rev1 primers and therefore PCR products bearing the same MID tags are clustered together in the same well. |
| B | Breed | Breed of animal – HF (Holstein-Friesian), Boran, WF (White Fulani), RF (Red Fulani), G (Goudali), M (mixed/cross-breed), X (no sample) |
| C | ID | Animal Identification |
| D | Total pairs | Number of paired reads sequenced for this well (combining For1/Rev2 and For3/Rev1) |
| E | Overlapping pairs | Number of paired reads sequenced for this well (combining For1/Rev2 and For3/Rev1) that overlap to form a contiguous sequence |
| F | For1Rev2 | Number of reads in well that have the For1Rev2 primers |
| G | For3Rev1 | Number of reads in well that have the For3Rev1 primers |
|  |  |  |
| I/Z | Total For1/Rev2 or For3/Rev1 reads |  |
| J/AA | Total For1/Rev2 or For3/Rev1 variants |  |
| K/AB | Threshold | Number of reads required for a variant to pass the 0.2% cut-off threshold |
| L/AC | Variants removed by cut-off | Number of variants that fail to reach threshold |
| M/AD | Reads removed by cut-off | Number of reads removed by application of threshold |
| N/AE | Singletons | Number of variants removed by threshold that were represented by a single read |
| O/AF | Variants above threshold | Number of variants above threshold |
| P/AG | Reads above threshold | Number of reads that form dataset to be entered into MHCI analysis pipeline |
| Q/AH | Chiamaeras | Number of chiamaeras identified |
| R/AI | Length >+/-9bp difference | Number of variants with length that is >+/-9bp different from that anticipated |
| S/AJ | 1/2bp variant | Number of variants that are 1 or 2bp different from variants in the sample that are present at >30x or >50x frequency (variants potentially arising from PCR/sequencing errors) |
| T/AK | Known classical MHCI | Number of variants matching known classical MHCI alleles |
| U/AL | Known non-classical MHCI | Number of variants matching known non-classical MHCI alleles |
| V/AM | Unknown | Novel variants that have passed all parameters in pipeline and have the anticipated length– putative novel MHCI alleles |
| W/AN | Length <9bp difference | Novel variants that have passed all parameters in pipeline but have an unanticipated length - putative novel MHCI alleles. |
| X/AO | Corrected number of variants | Variants above threshold minus numbers removed as chimaeras, having lengths >+/-bp difference or 1/2bp variant. |

**Spreadsheet 2 and 3 – ‘For1Rev2’ and ‘For3Rev1’**

Provide a description of the data obtained for each individual sample for the For1/Rev2 and For3/Rev2 reactions respectively. In these spread sheets the following data for each sample is given:

| Column | Parameter | Notes |
| --- | --- | --- |
| A | Well |  |
| B | Breed | Breed of animal – HF (Holstein-Friesian), Boran, WF (White Fulani), RF (Red Fulani), G (Goudali), M (mixed/cross-breed), X (no sample) |
| C | Identification | Animal Identification |
| D/E/F | Identified haplotypes | Designated when the combination of alleles that form a known haplotype are identified in a sample. |
| G/H/I | Alleles assigned to haplotypes | Lists each known MHCI allele in the identified haplotype and the percentage of the total overlapping reads for that sample which are represented by each of these alleles.* |
| J/K | Alleles unassigned to identified haplotypes | Lists each known MHCI allele that is not part of an identified haplotype in the sample and the percentage of the total overlapping reads for that sample which are represented by each of these alleles. |
| L/M/N/O | Unknown | Lists each variant of the anticipated length for the PCR which is not a known MHCI allele and the percentage of the total overlapping reads for that sample which are represented by each of these variants. In the For1Rev2 and For3Rev1 spreadsheet these variants are designated as ‘Foo’ and ‘Bar’ respectively, with consecutive numerical identifiers give to each variant. |
| P/Q | Unknown > +/- 9bp difference | Lists each variant differing from the anticipated PCR product length by <9bp which is not a known MHCI allele and the percentage of the total overlapping reads for that sample which are represented by each of these variants. In the For1Rev2 and For3Rev1 spreadsheet these variants are designated as ‘FooSZ’ and ‘BarSZ’ respectively, with consecutive numerical identifiers give to each variant. |
| R/S | Known non-classical | Lists each known non-classical MHCI allele and the percentage of the total overlapping reads for that sample which are represented by each of these alleles. |

*when a sequence could match to multiple alleles each potential allele is shown unless the allele can be deduced from the co-expressed alleles.

**Spreadsheet 4 – ‘Foo Bar’**

Provides 2 sets of information regarding the Foo and Bar sequences.

1 – A list of Foo and Bar sequences identified from the For1Rev2 and For3Rev1 data that match and are therefore considered to represent products of the same allele.

2 – A summary of BLAST analysis of each Foo and Bar sequence against the database of known bovine MHCI alleles. For each Foo/Bar sequence the known MHCI allele to which it showed the highest BLAST value is shown as is the % identity, length of sequence matched and the e-value.**

**The current chimaera detection algorithm may not detect chiamaeras if one parent sequence is different from the canonical product size. Consequently any unknown variants identified ***in samples which contain sequences known to generate products of different lengths (e.g. 3*03301N) and at a lower read frequency than such sequences*** have to be manually checked for chimaera. Such unknown variants are identified and highlighted in the accompanying ssample excel spreadsheet.
