# Supplementary material for: Rapid identification of bovine MHCI haplotypes in genetically divergent cattle populations using next-generation sequencing
Source: Immunogenetics. 2016 Aug 11;68(10):765–81. doi: 10.1007/s00251-016-0945-7 (PMC5056950; doi:10.1007/s00251-016-0945-7)
Supplement: Supplementary file 8 — Tables summarising allele dropout and ambiguity. Table 1a List of haplotypes containing alleles not consistently present above the 0.2 % read frequency cut-off threshold–partial allele dropout. Table 1b List of haplotypes containing alleles which were only detected following amplification with either the For1Rev2 or For3Rev1 primer pairs–complete allele dropout. Table 2 Details of novel MHCI alleles that cannot be discriminated following sequencing of the amplicons generated by the For1/Rev2 and For3/Rev1 primer pairs. (DOCX 21 kb) [file 251_2016_945_MOESM8_ESM.docx]

**Supplementary Data 5**

**1 - Allele Dropout**

**Table 1a – List of haplotypes containing alleles not consistently present above the 0.2% read frequency cut-off threshold – ‘partial allele dropout’.**

| Haplotype | Alleles consistently in haplotype | | | | Allele not consistently present above cut-off threshold | Missing from* | |
| --- | --- | --- | --- | --- | --- | --- | --- |
|  |  |  |  |  |  | For1Rev2 | For3Rev1 |
| A10 | 3*00201 | 2*01201 |  |  | Roslin1.1 | 19/24 |  |
| A10 | 3*00201 | 2*01201 |  |  | RoslinSZ.1.1 | 8/24 | 9/27 |
| A11 | 3*01701 | 2*01801 |  |  | 3*03301N | 11/33 | 12/37 |
| A13 | 1*03101 | 2*03201N |  |  | Roslin1.2 | 3/13 |  |
| HP1.2 | Roslin1.7 | Roslin1.8 | 3*02702 |  | 6*04001 | 1/3 |  |
| HP2.8 | Roslin2.17 | Roslin2.18 |  |  | Roslin2.19 | 8/15 |  |
| HP2.9 | Roslin2.20 | 3*00102 |  |  | RoslinSZ.2.1 | 8/8 | 5/7 |
| HP2.11 | Roslin2.23 | Roslin2.24 |  |  | Roslin2.19 | 2/7 |  |
| HP2.12 | Roslin2.25 | Roslin2.26 |  |  | Roslin2.27 | 2/3 |  |
| HP2.13 | Roslin2.28 | 4*02401 | 2*02501 |  | 6*04001 | 6/9 |  |
| HP2.15 | Roslin2.31 | 3*00102 |  |  | RoslinSZ.2.1 | 3/6 | 2/6 |
| HP2.22 | Roslin2.42 |  |  |  | RoslinSZ.3.2 | 5/6 | 3/6 |
| HP3.6 | Roslin3.10 | Roslin3.11 |  |  | Roslin1.1 | 1/2 |  |
| HP3.20 | Roslin2.35 | Roslin3.30 | Roslin3.32 | Roslin3.31 | Roslin1.1 | 7/7 | 4/7 |
| HP3.26 | Roslin3.39 | Roslin3.23 | Roslin3.40 |  | Roslin3.41 | 4/5 |  |
| HP3.27 | Roslin3.42 |  |  |  | Roslin3.43 | 2/3 |  |

* shows the number of samples in which the allele was not present above the 0.2% cut-off threshold.

**Table 1b - List of haplotypes containing alleles which were only detected following amplification with either the For1Rev2 or For3Rev1 primer pairs – ‘complete allele dropout’**

| Haplotype | Alleles consistently present in haplotype | | | Alleles only detected in For1Rev2 or For3Rev1 | |  |
| --- | --- | --- | --- | --- | --- | --- |
|  |  |  |  | For1Rev2 | For3Rev1 |  |
| HP1.3# | Roslin1.9 | Roslin1.10 | 2*01602 | Roslin(For1Rev2).1.1 |  |  |
| HP3.23# | Roslin3.35 |  |  | Roslin(For1Rev2).3.1 |  |  |
| HP3.27# | Roslin3.42 | Roslin3.43 |  | Roslin(For1Rev2).3.1 |  |  |
| HP3.14## | Roslin3.20 |  |  | RoslinSZ(For1Rev2).3.1 | Roslin(For3Rev1).2.4/Roslin(For3Rev1).3.2 |  |
| HP2.1# | Roslin2.1 | Roslin2.2 | 3*00402 |  | Roslin(For3Rev1).2.1 |  |
| HP2.6# | Roslin2.13 |  |  |  | Roslin(For3Rev1).2.2 |  |
| HP2.19# | Roslin2.47 | Roslin2.48 | Roslin2.49 |  | Roslin(For3Rev1).2.4 |  |
| HP2.26# | Roslin2.45 |  |  |  | Roslin(For3Rev1).2.2 |  |
| HP2.27# | Roslin2.46 |  |  |  | Roslin(For3Rev1).2.3 |  |
| HP3.18# | Roslin3.25 | Roslin3.26 |  |  | Roslin(For3Rev1).2.3 |  |
| HP3.22# | Roslin3.34 | 2*04701 | RoslinSZ.3.1 |  | Roslin(For3Rev1).3.1 |  |
| HP3.24# | Roslin3.36 | Roslin3.37 | 2*01601 |  | Roslin2.27* |  |
| HP3.28# | Roslin3.44 |  |  |  | Roslin(For3Rev1).3.3 |  |
| HP3.31# | Roslin2.1 | Roslin3.47 | 3*00402 |  | Roslin(For3Rev1).3.1 |  |

* In HP2.12 allele Roslin2.27 is amplified by both For1Rev2 and For3Rev1 primer pairs, suggesting that Roslin2.27 actually represents 2 distinct alleles and that the one present in HP3.24 contains a substitution in the For1 and/or Rev2 annealing site that prevents amplification

**2 – Non-discrimination of allelic variants**

**Table 2 –Details of novel MHCI alleles that can’t be discriminated following sequencing of the amplicons generated by the For1/Rev2 and For3/Rev1 primer pairs.**

|  | Primer pair | |
| --- | --- | --- |
|  | For1/Rev2 | For3/Rev1 |
| MHCI alleles not discriminated by sequencing of amplicons (ambiguous alleles) |  | 1*02901/Roslin2.43 |
|  |  | Roslin2.19/Roslin2.22 |
|  |  | Roslin2.38/Roslin2.25 |
|  |  | Roslin1.10/Roslin1.2 |
|  |  | 3*00402/3*05301/Roslin2.16 |
|  | 6*04101/Roslin2.30 |  |
|  | 1*06101/Roslin2.18 |  |
|  | 2*05401/Roslin3.8 |  |
|  | Roslin2.20/Roslin3.18 |  |
|  | Roslin2.40/Roslin3.1 |  |
|  | Roslin2.5/Unassigned2.18 |  |
|  | 2*01801/2*01802/Roslin3.46 |  |
