# Supplementary figures and images for: Rapid identification of bovine MHCI haplotypes in genetically divergent cattle populations using next-generation sequencing
Source: Immunogenetics. 2016 Aug 11;68(10):765–81. doi: 10.1007/s00251-016-0945-7 (PMC5056950; doi:10.1007/s00251-016-0945-7)

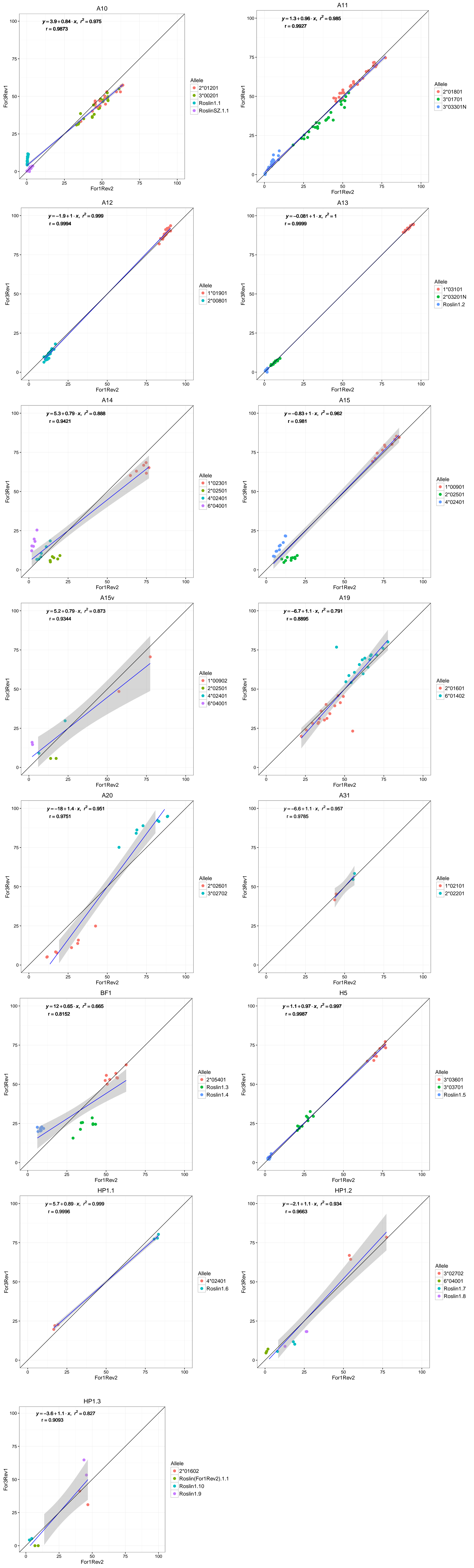

Supplement: Supplementary file 10 — Scatterplot analysis of the read frequency observed with the For1/Rev2 and For3/Rev1 PCR reactions for each individual haplotype identified in (A) the Holstein-Friesian cohort, (B) the Boran cohort and (C) the Cameroonian cohort. For each haplotype the read frequency for each allele from each individual for both For1/Rev2 and For3/Rev1 PCR reactions is shown. The line of best is represented and the equation describing this, the coefficient of correlation (r) and the correlation of determination (r2) are shown. (PDF 47 kb) [file 251_2016_945_MOESM10_ESM.pdf]

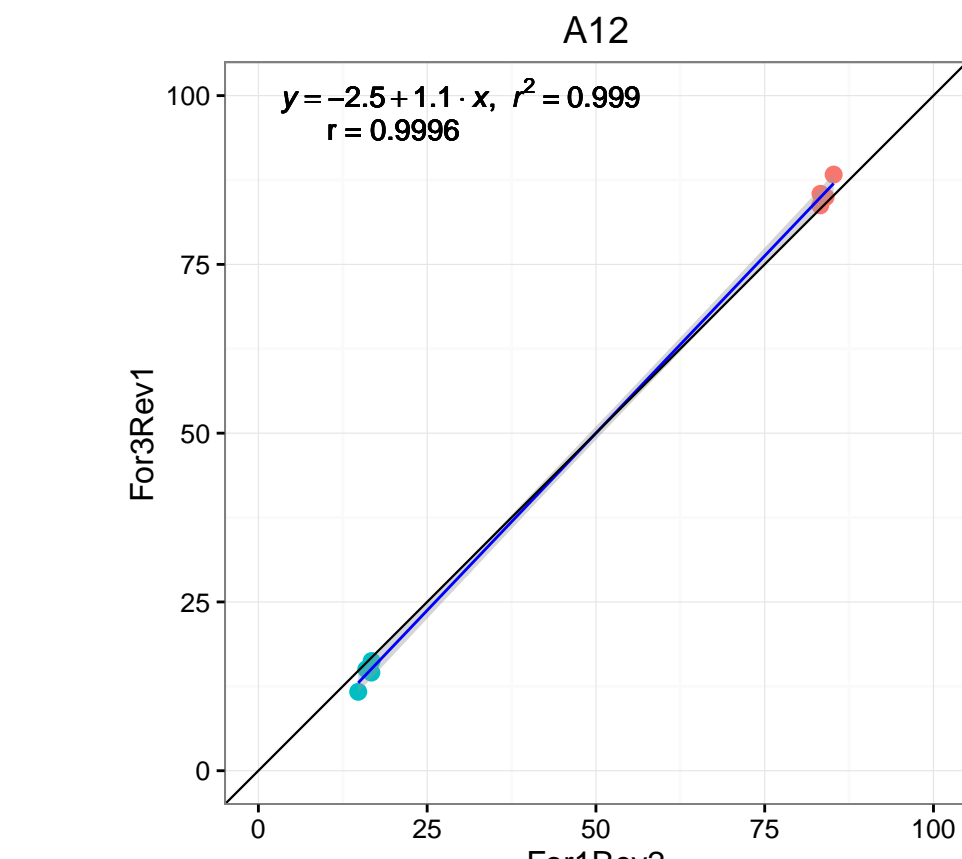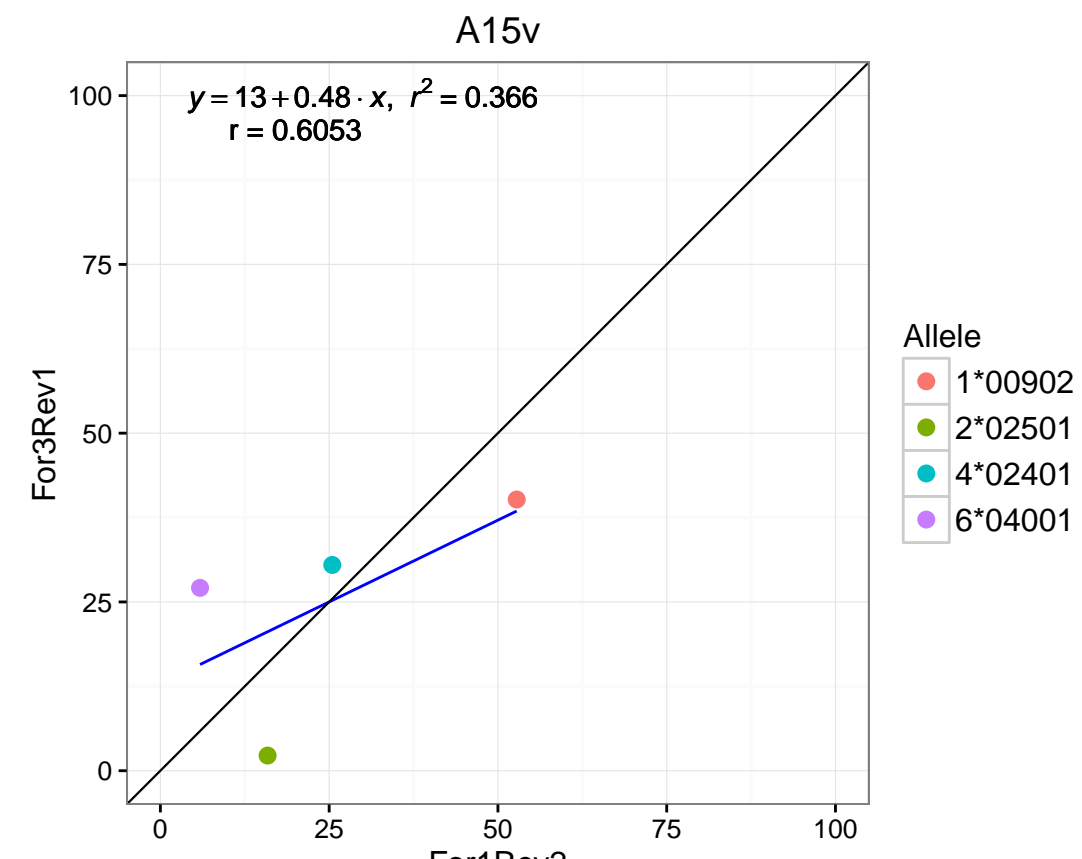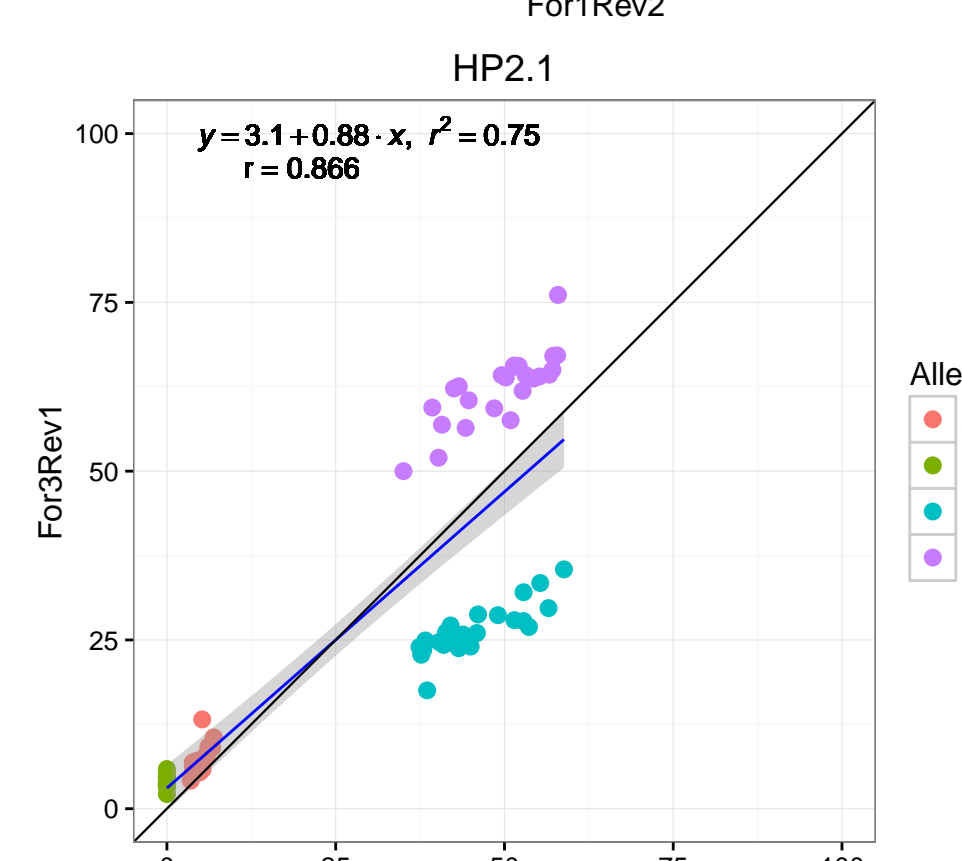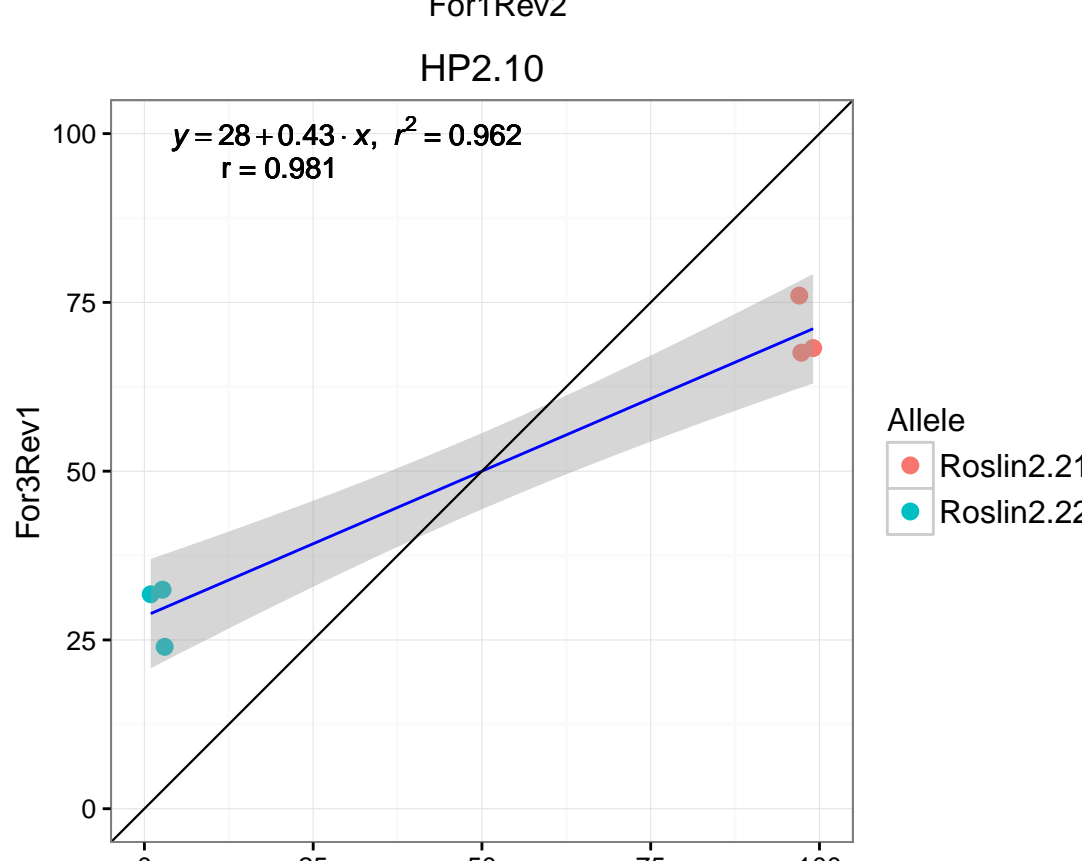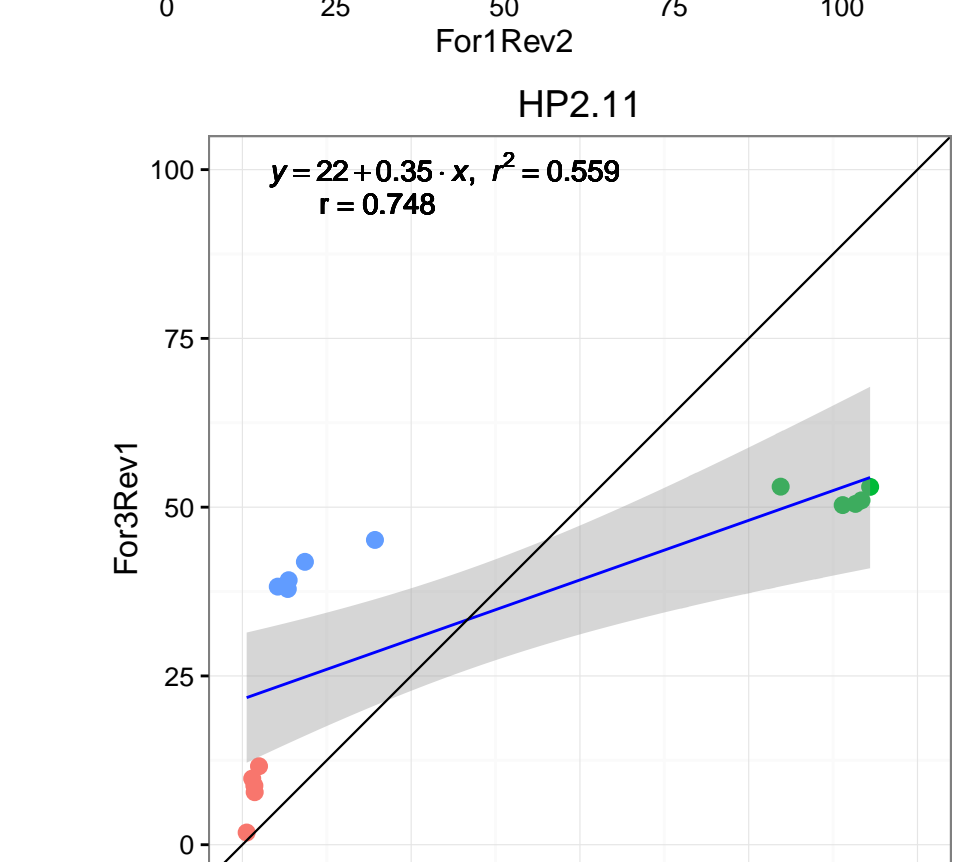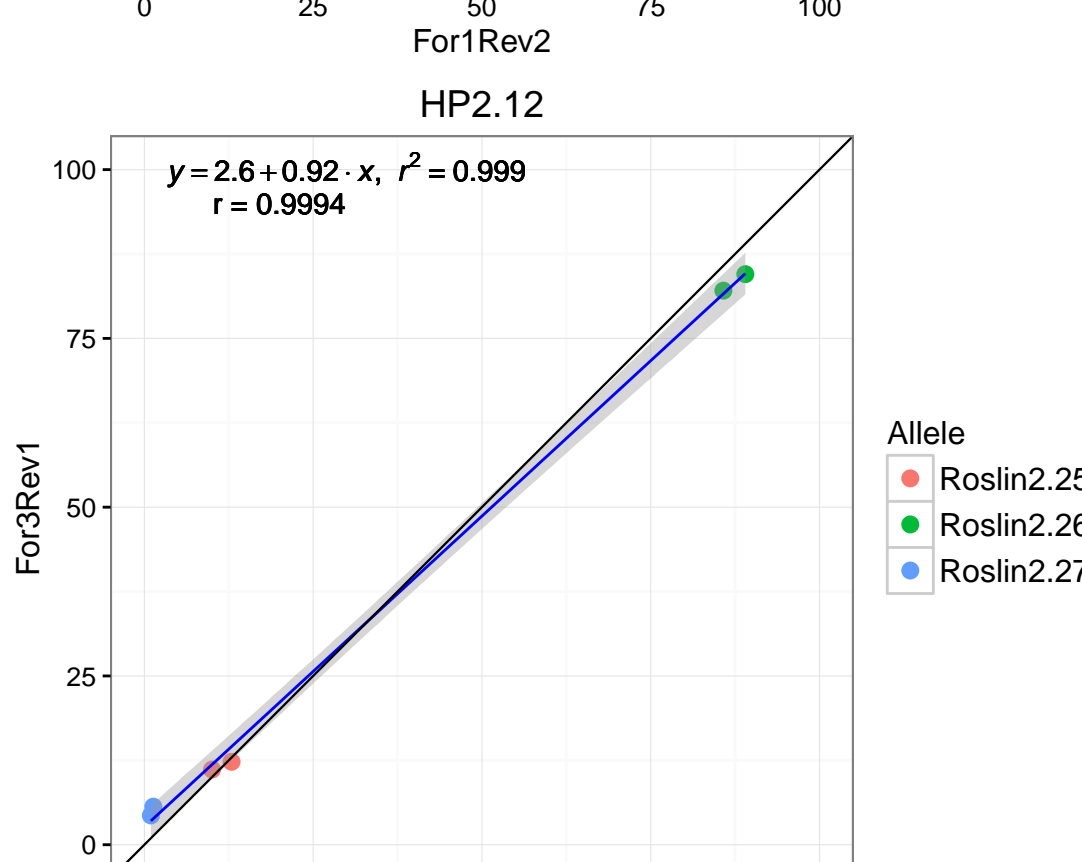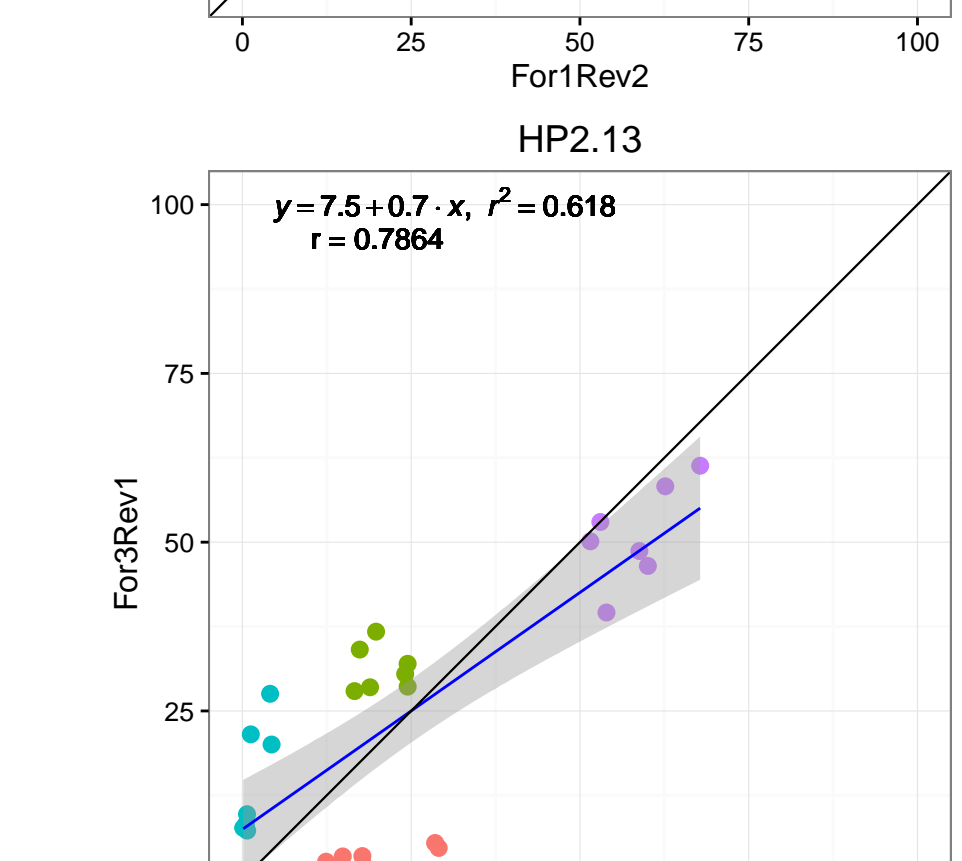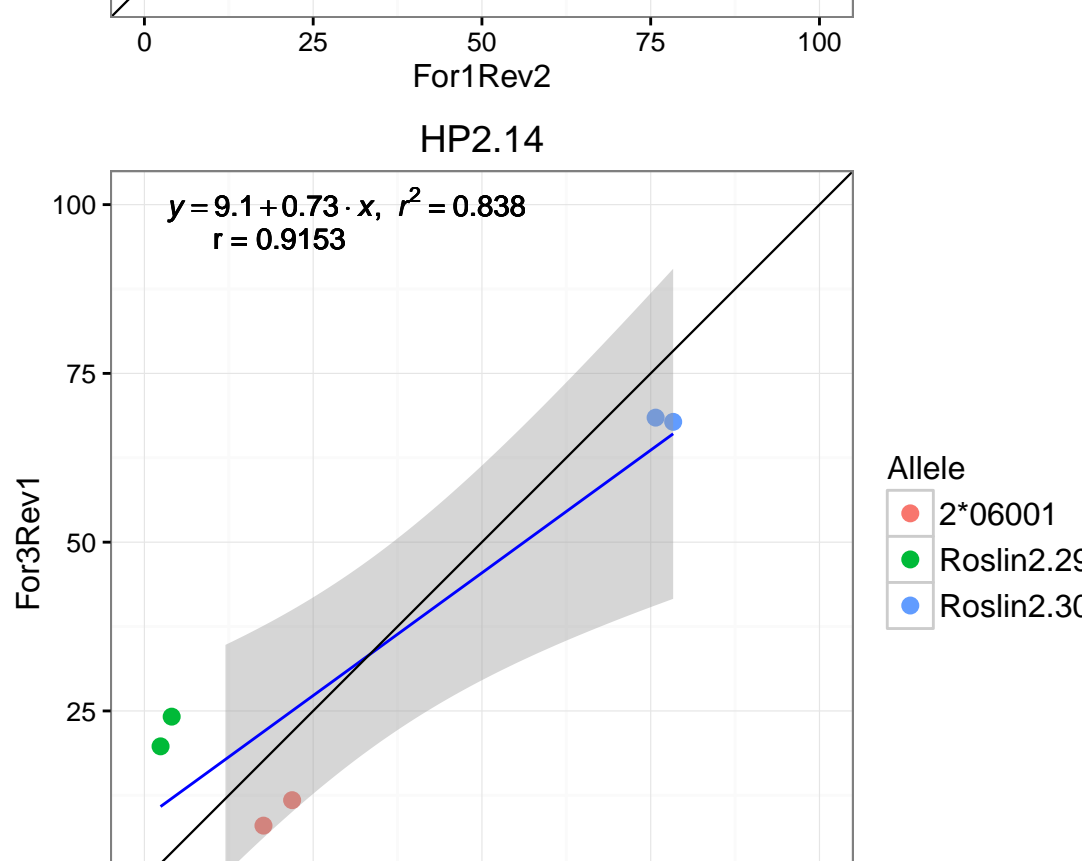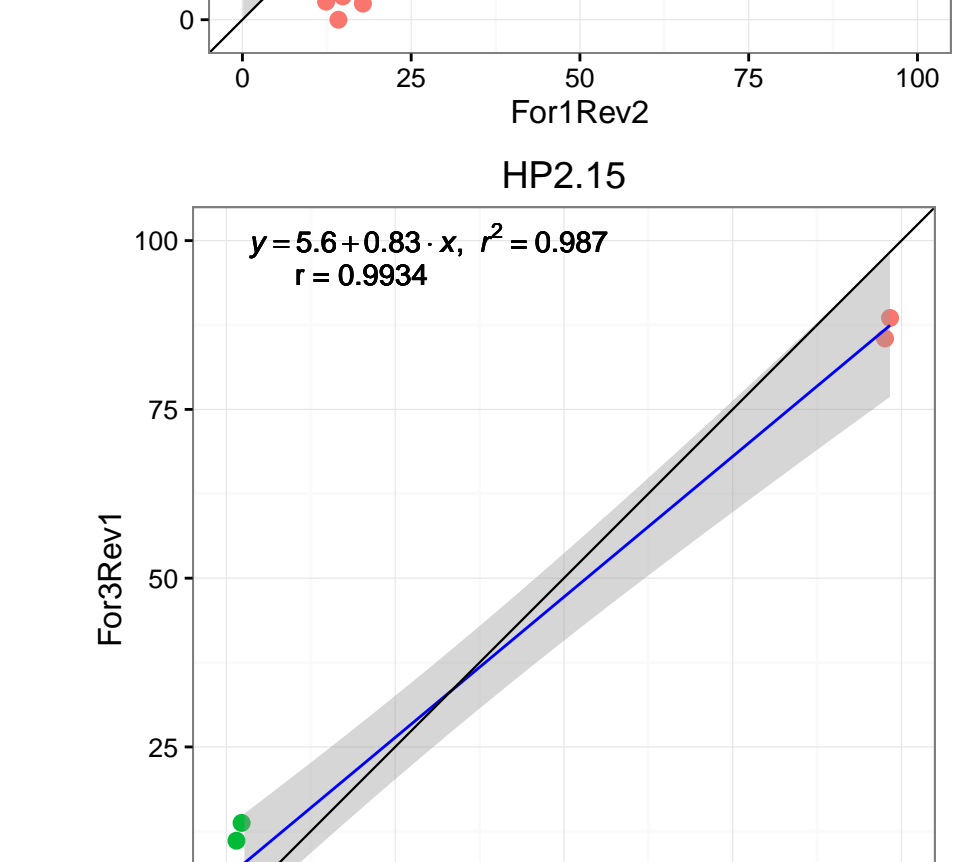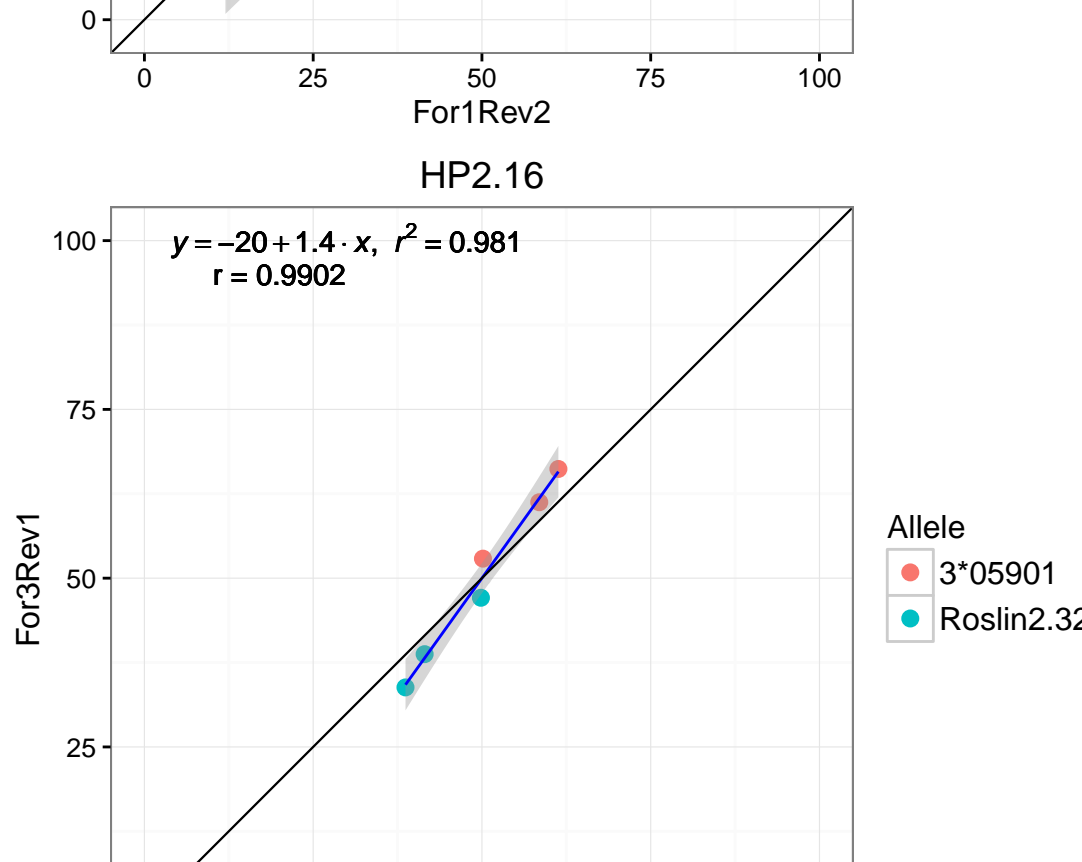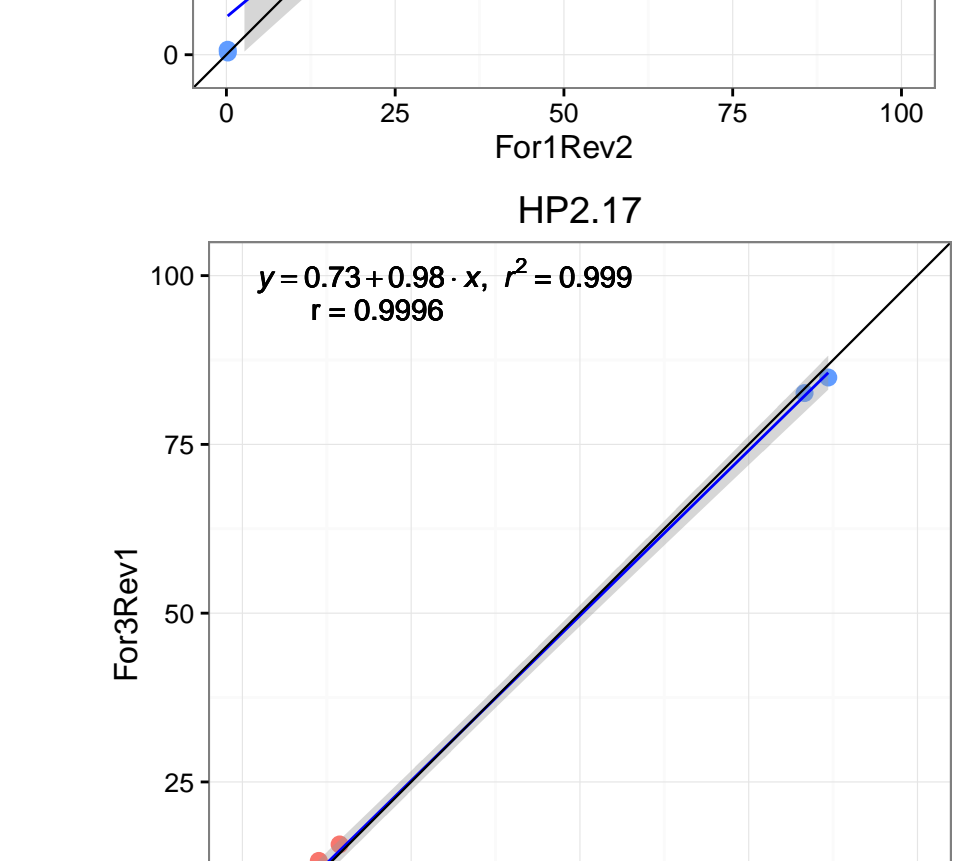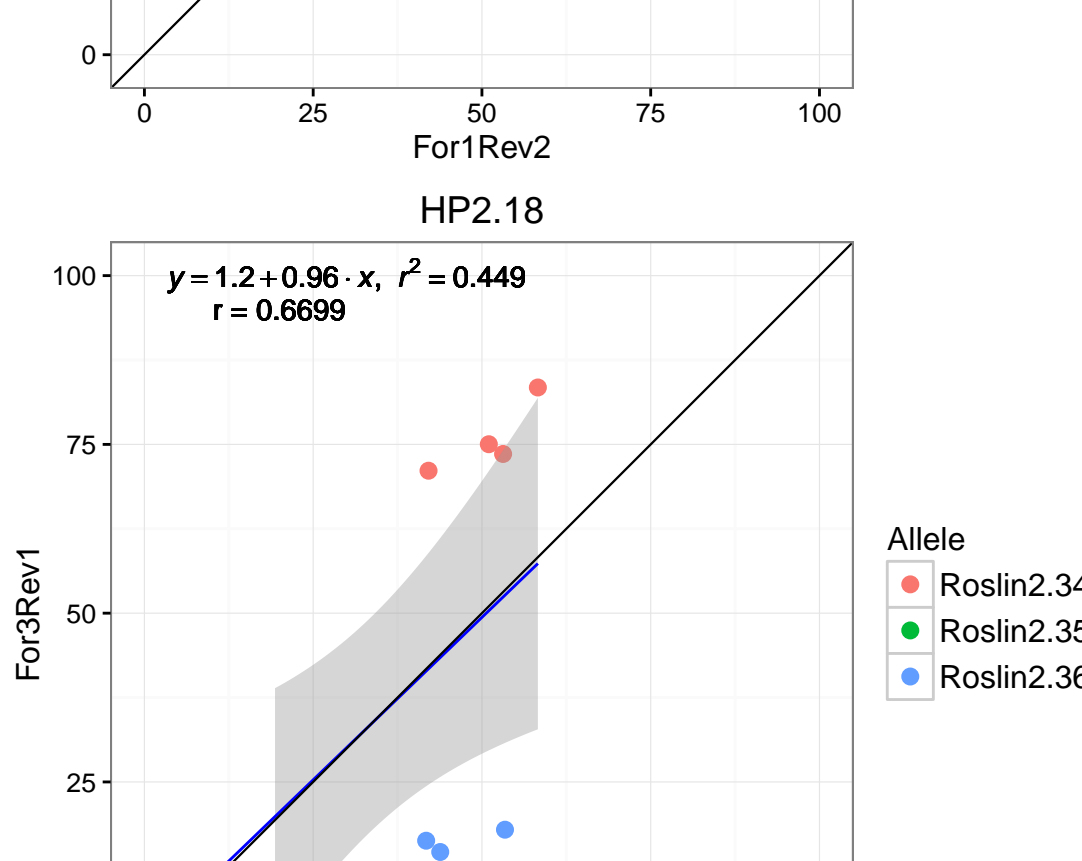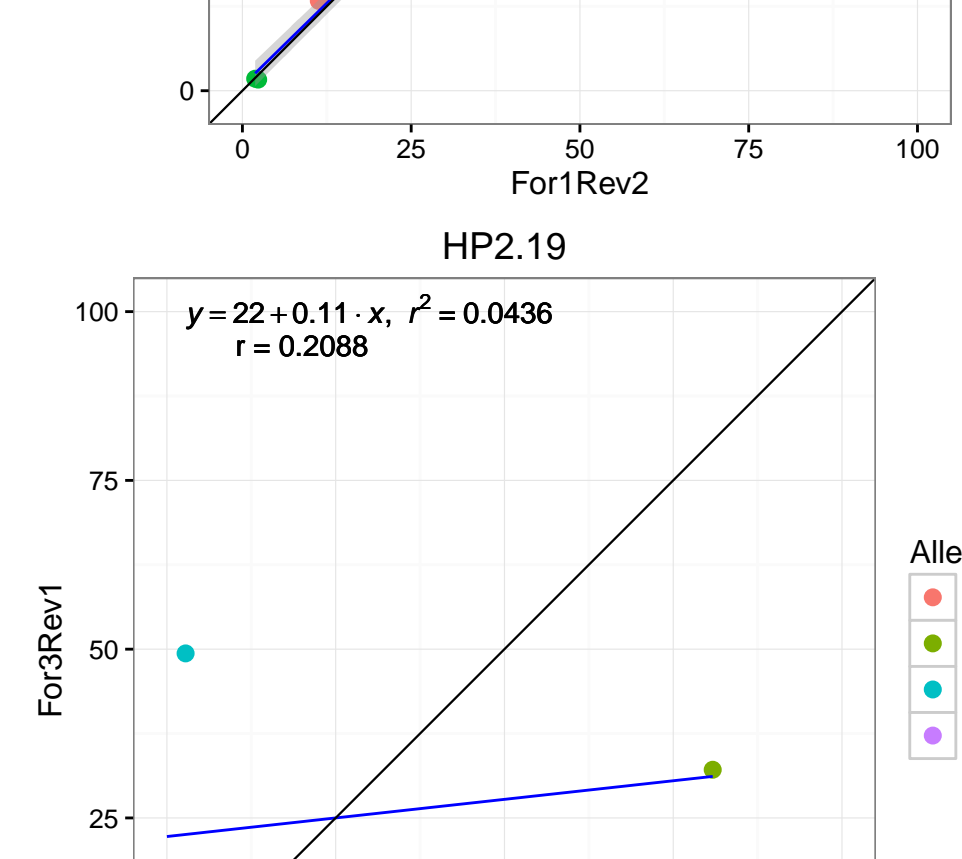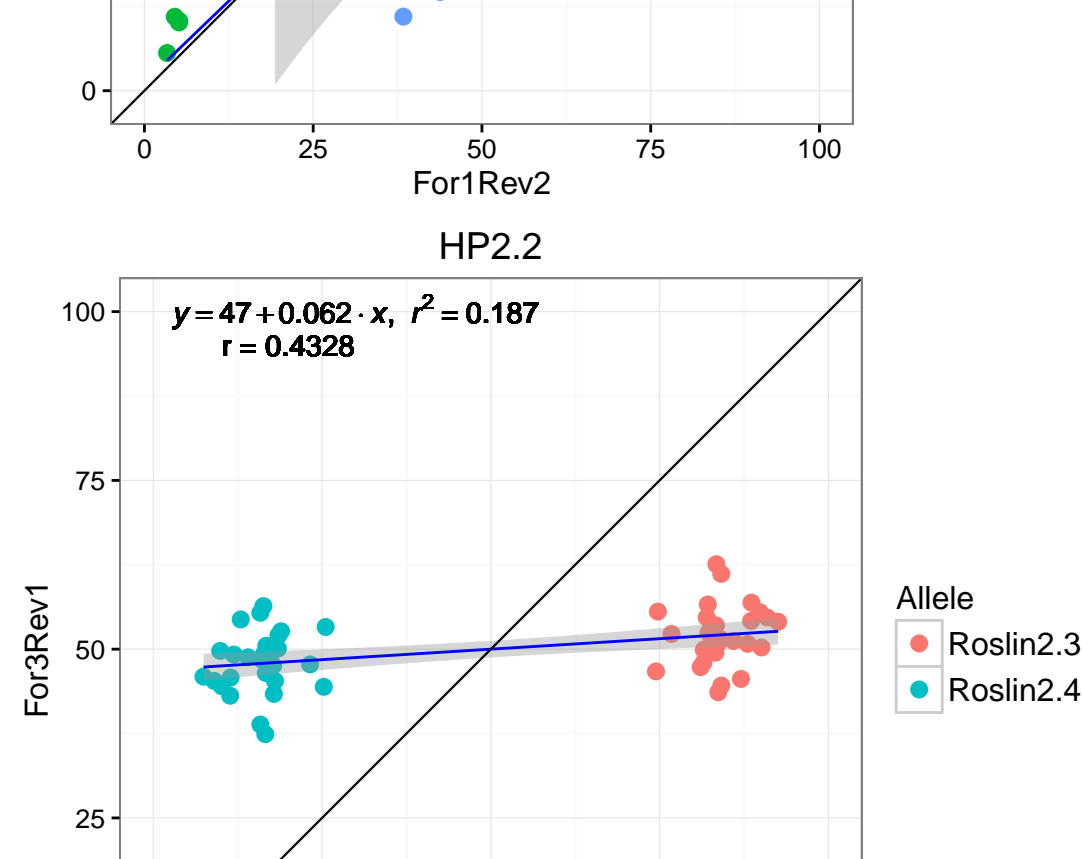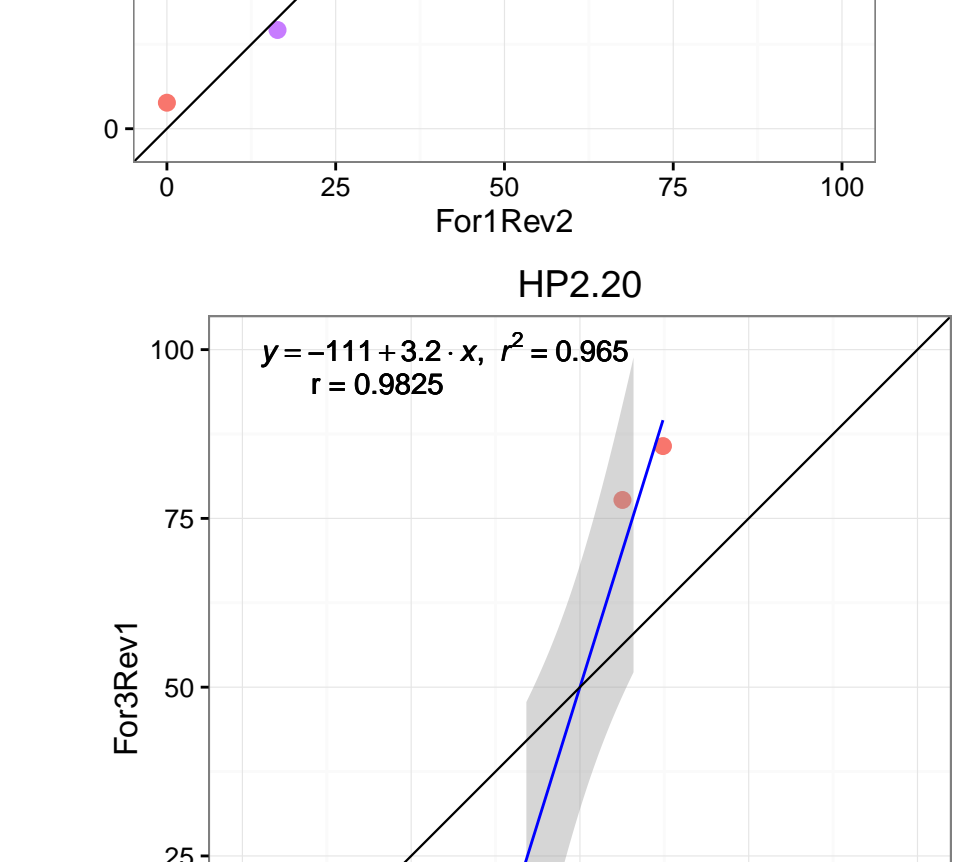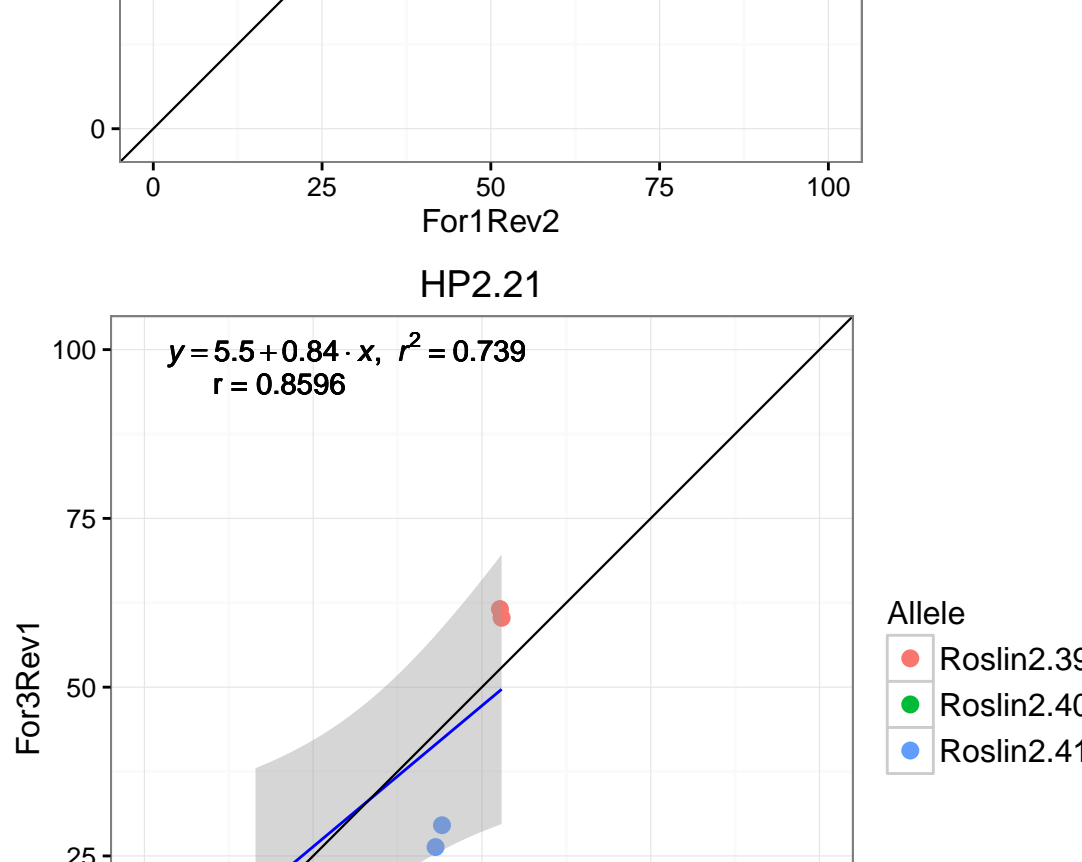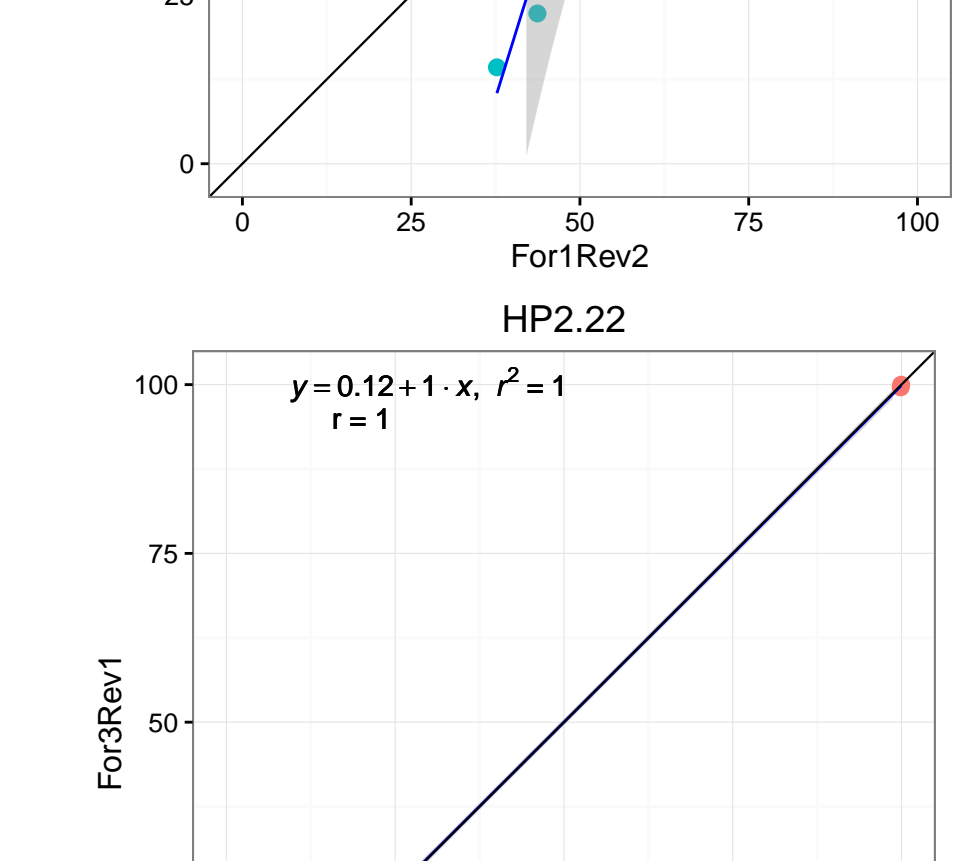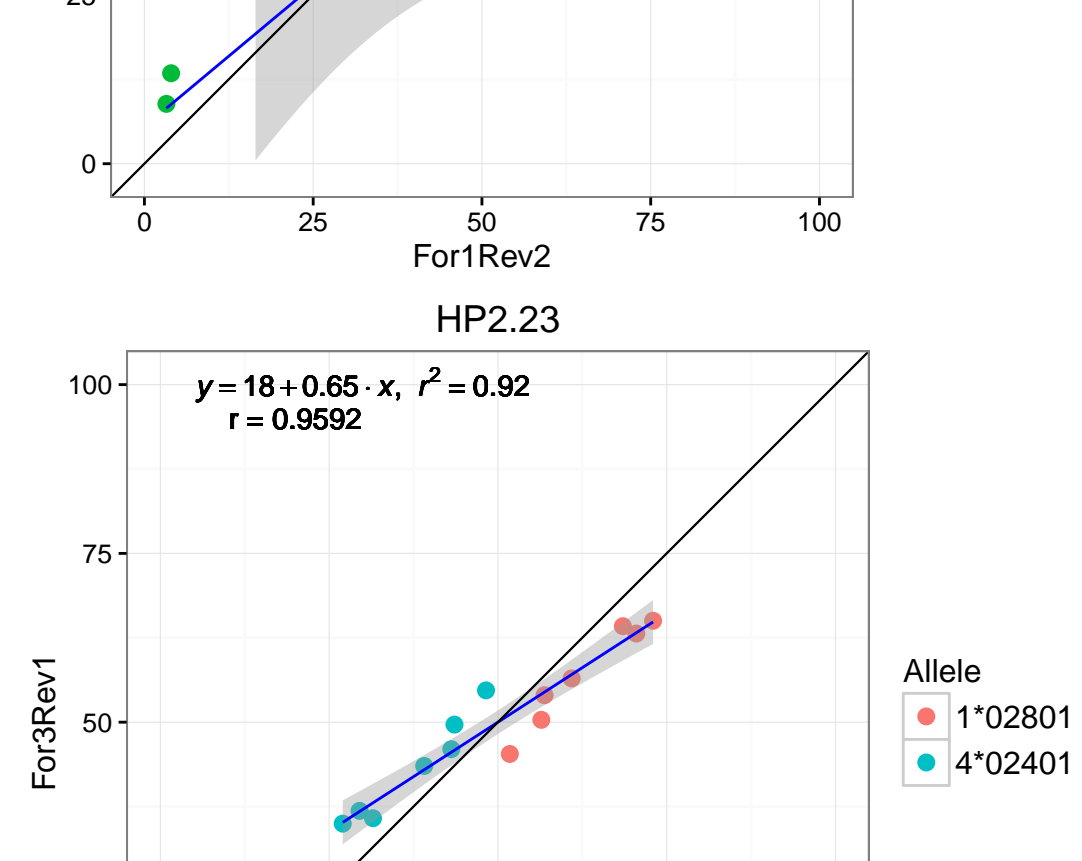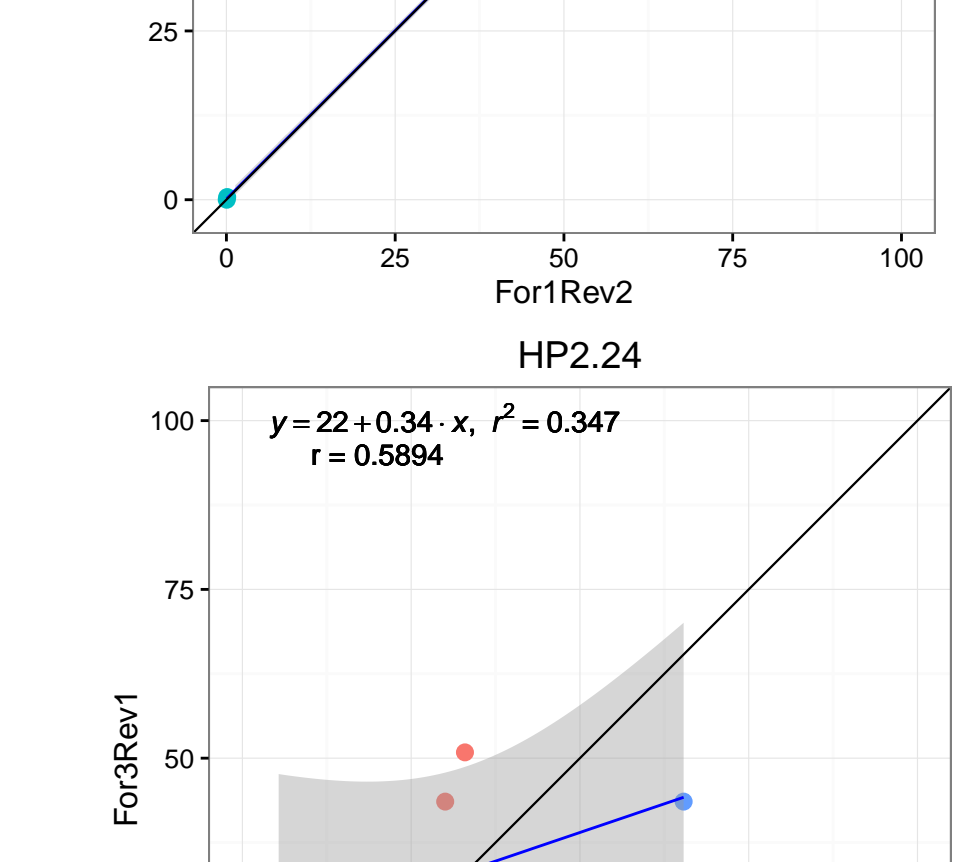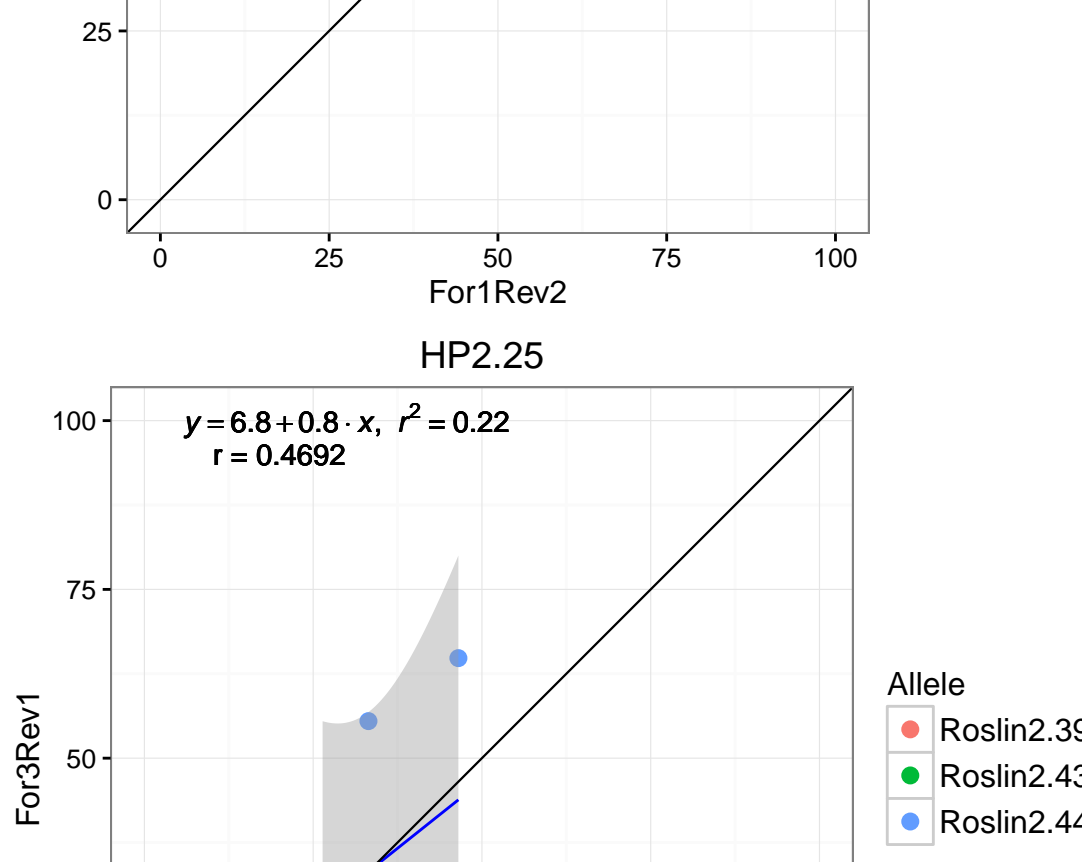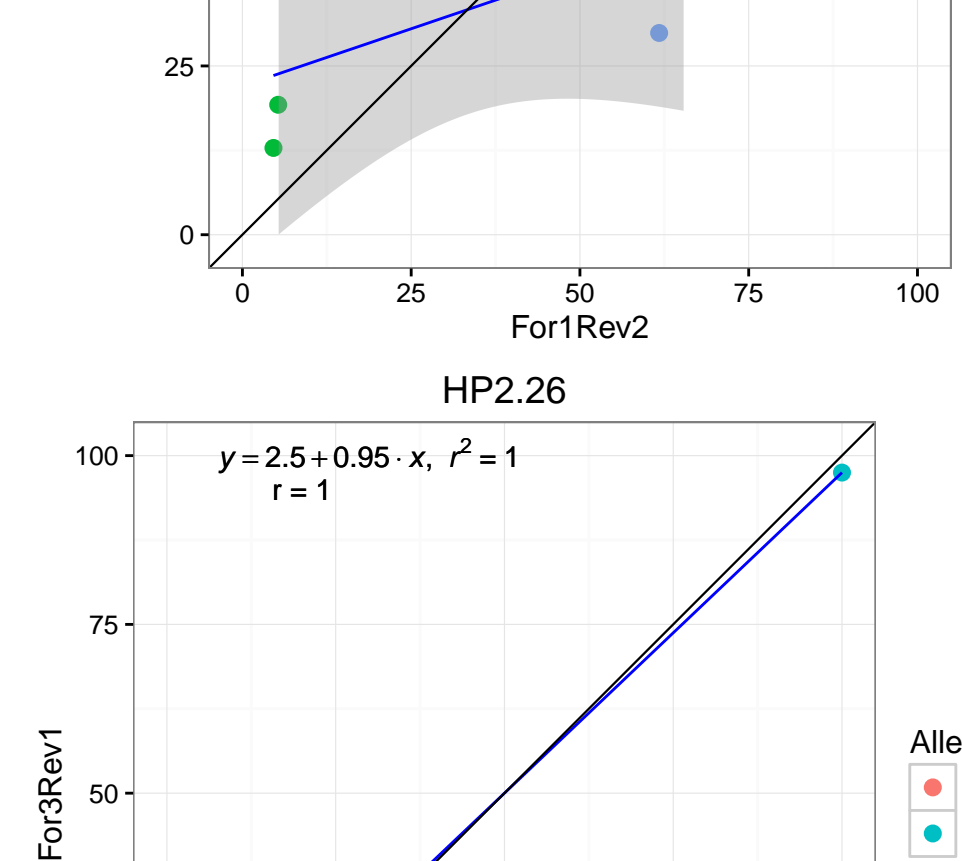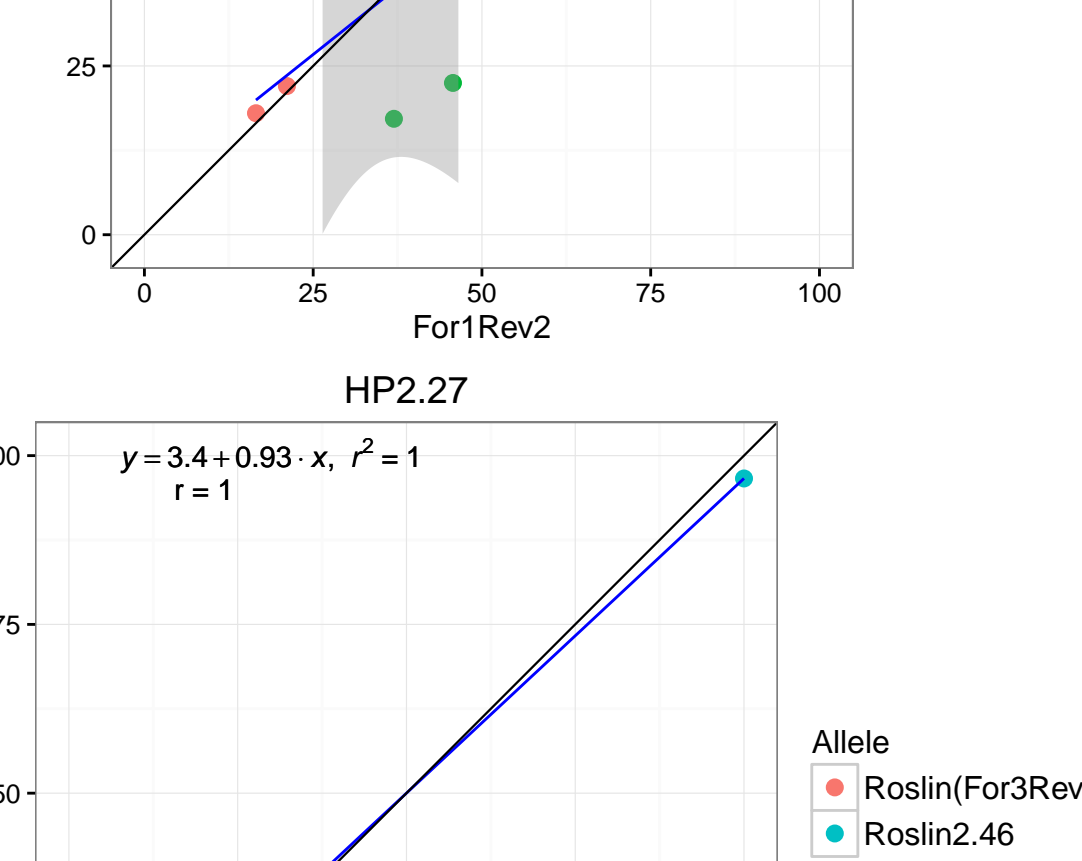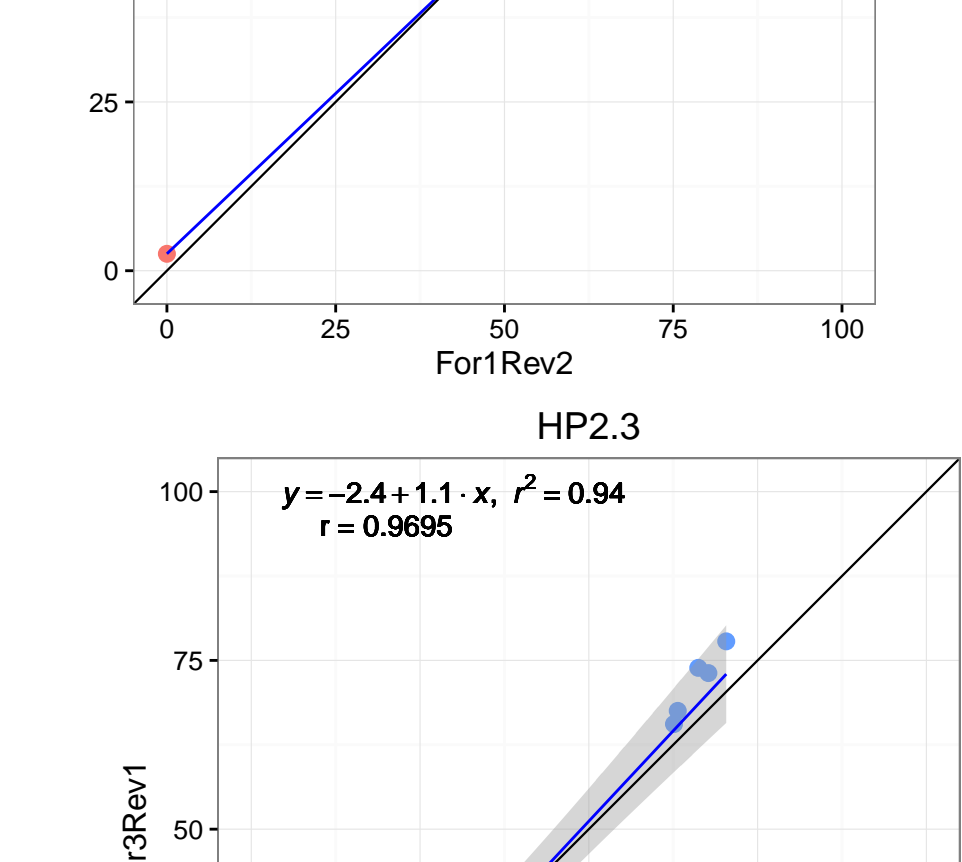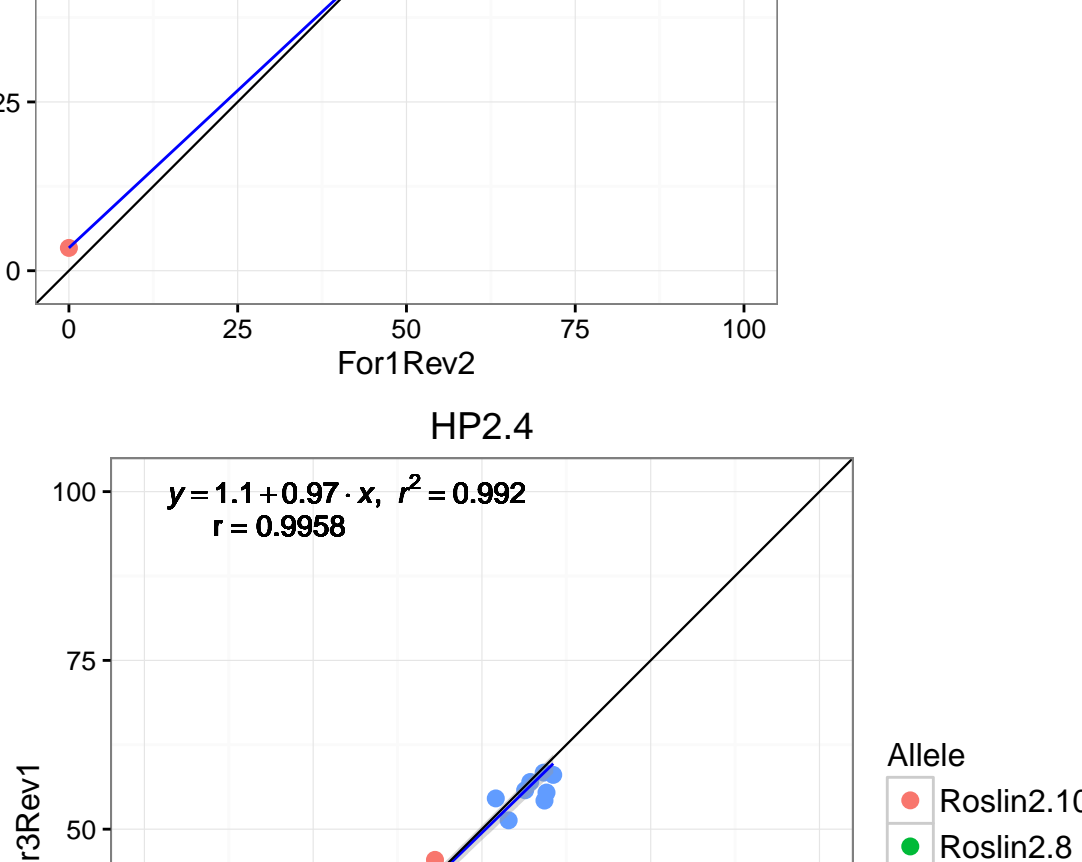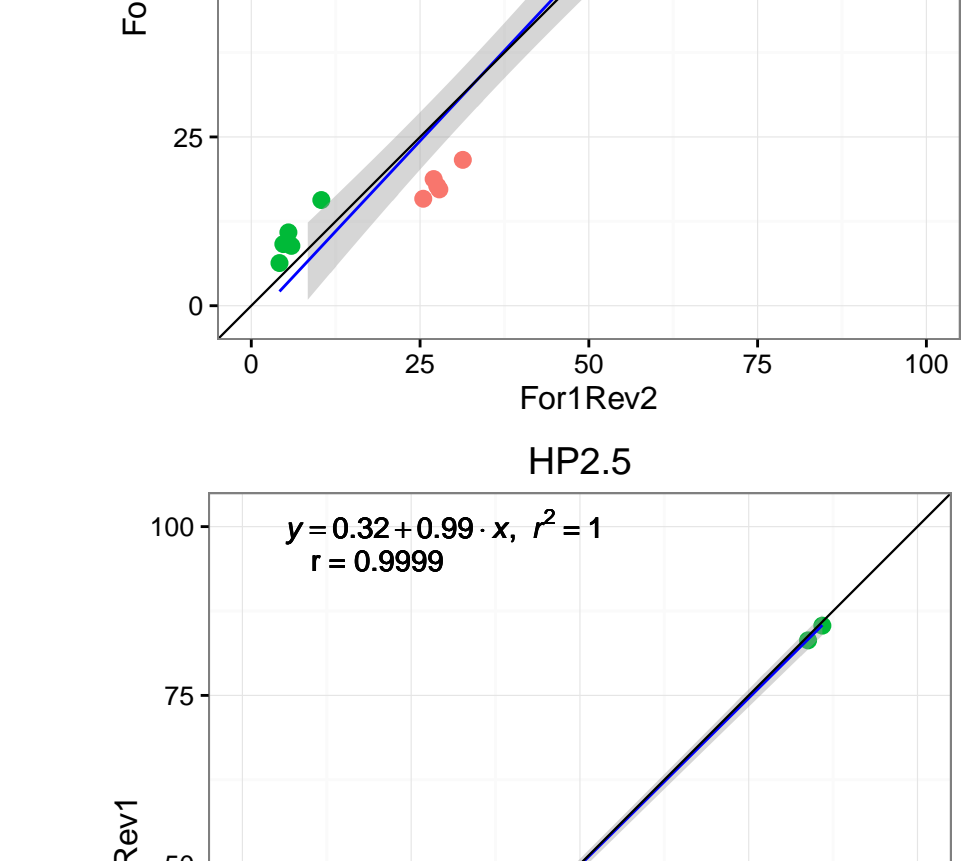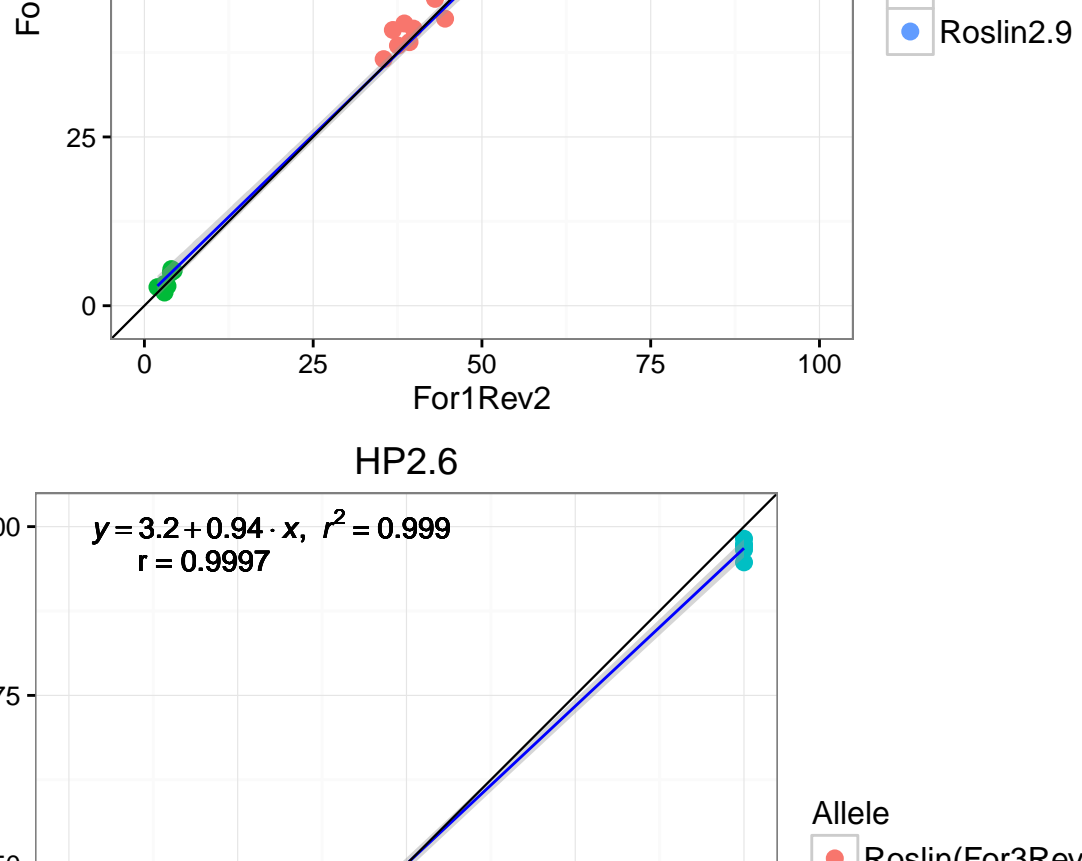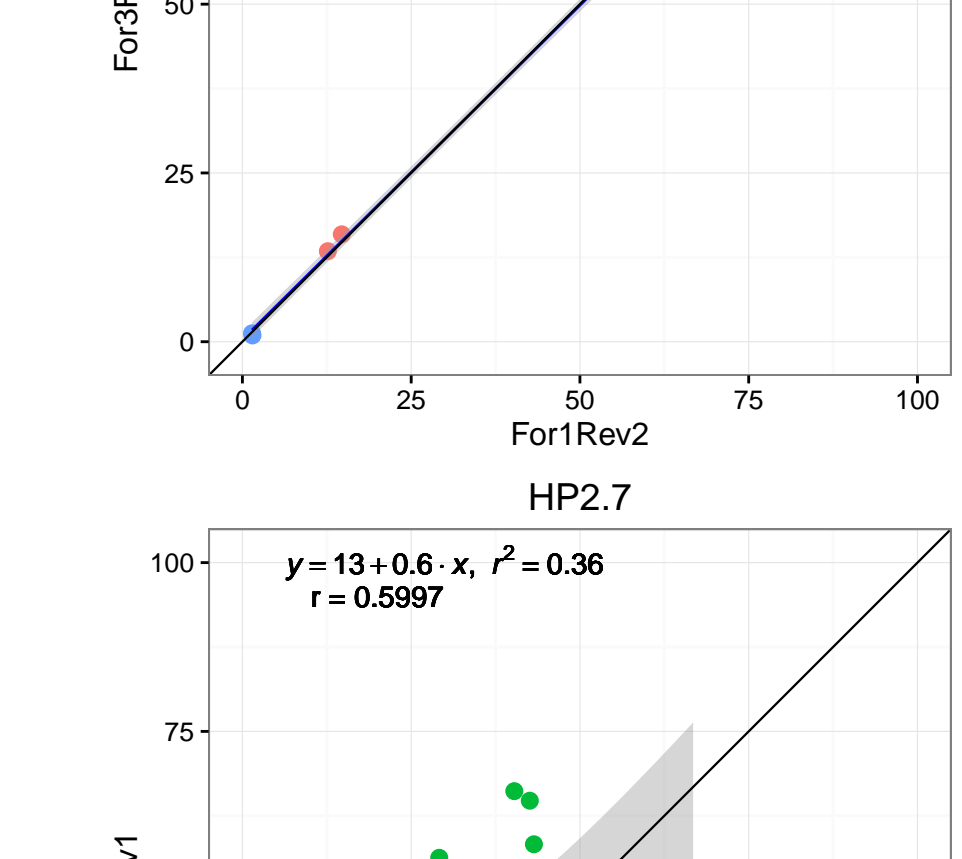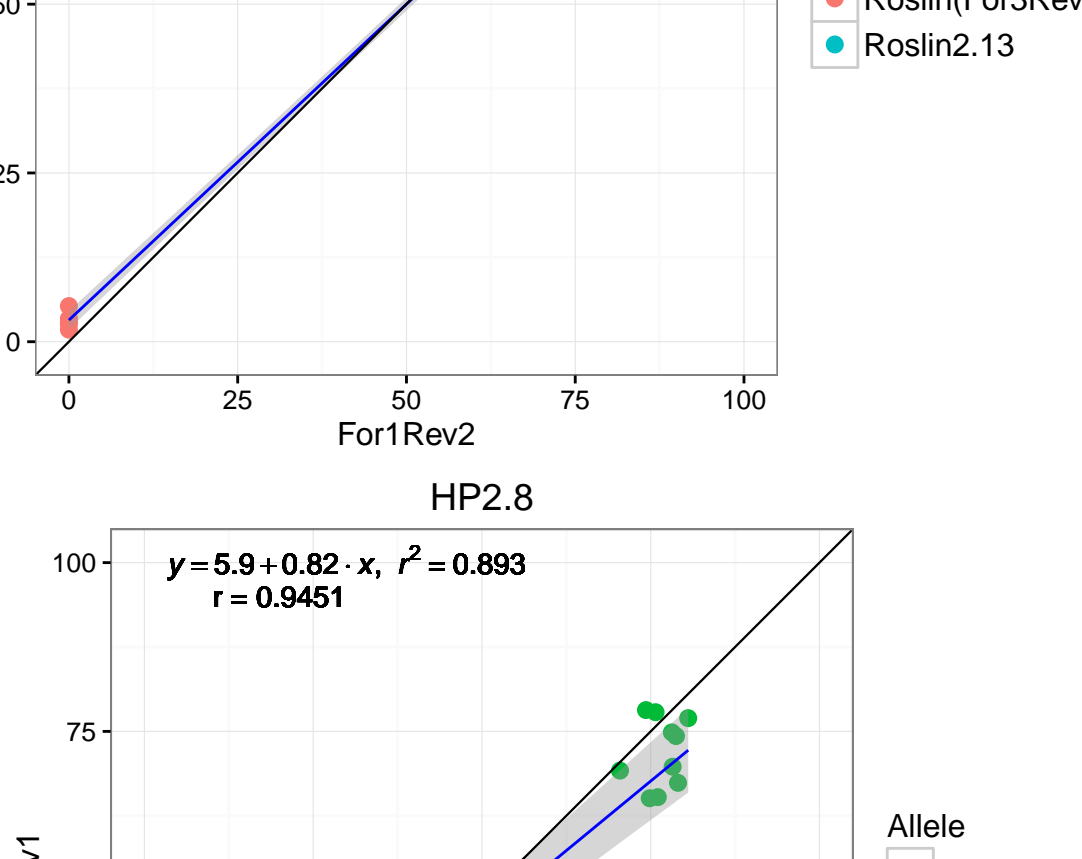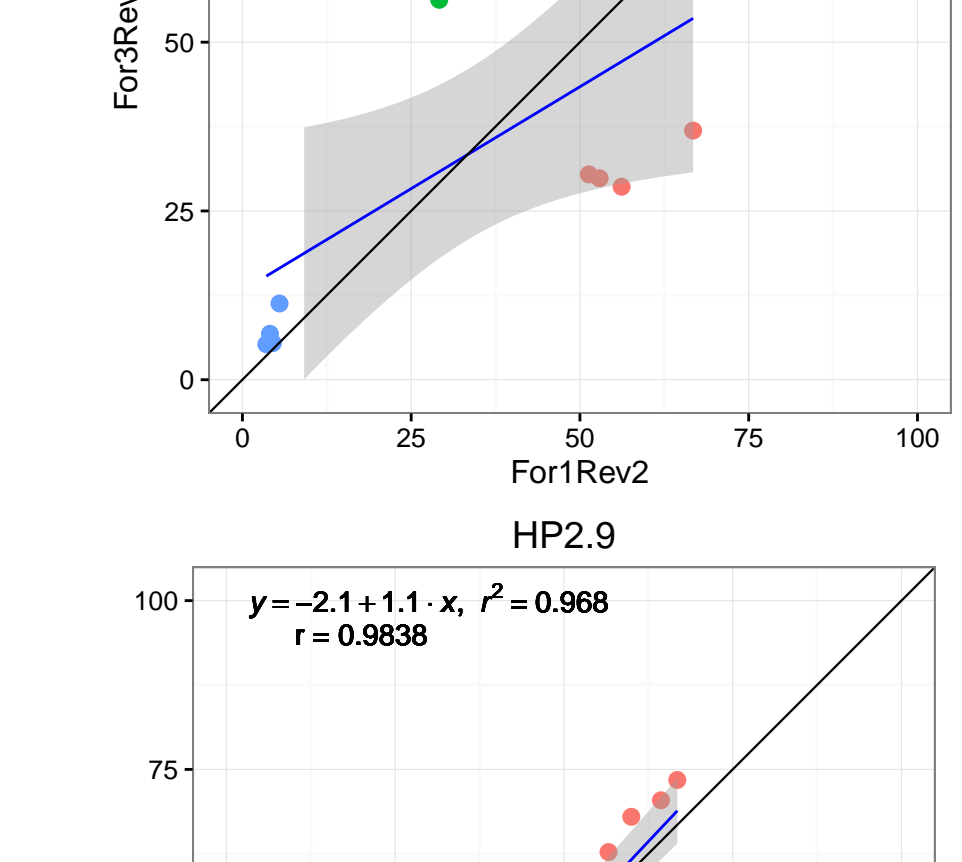

Supplement: Supplementary file 11 — (PDF 70 kb) [file 251_2016_945_MOESM11_ESM.pdf]

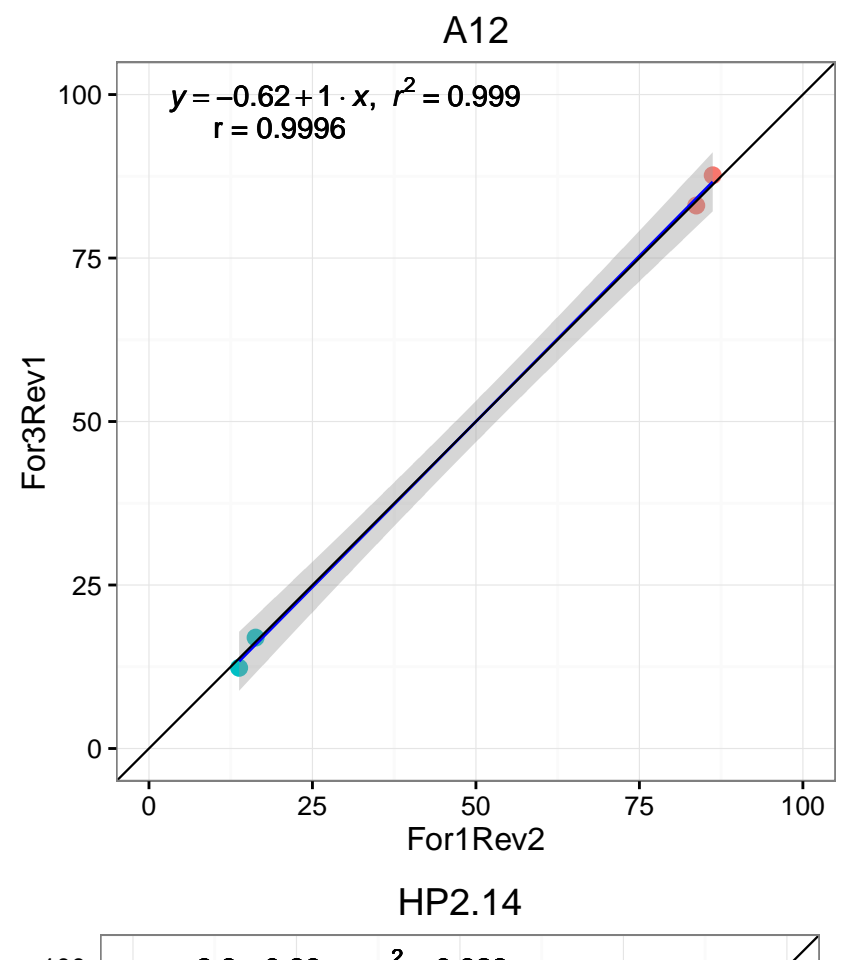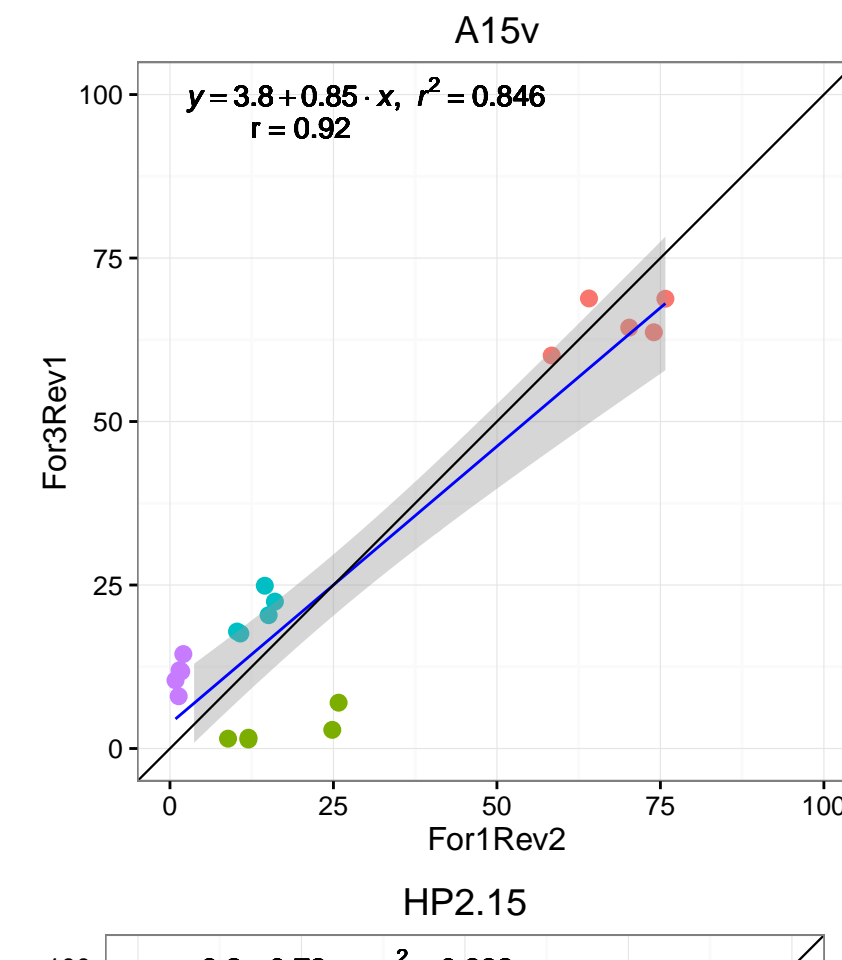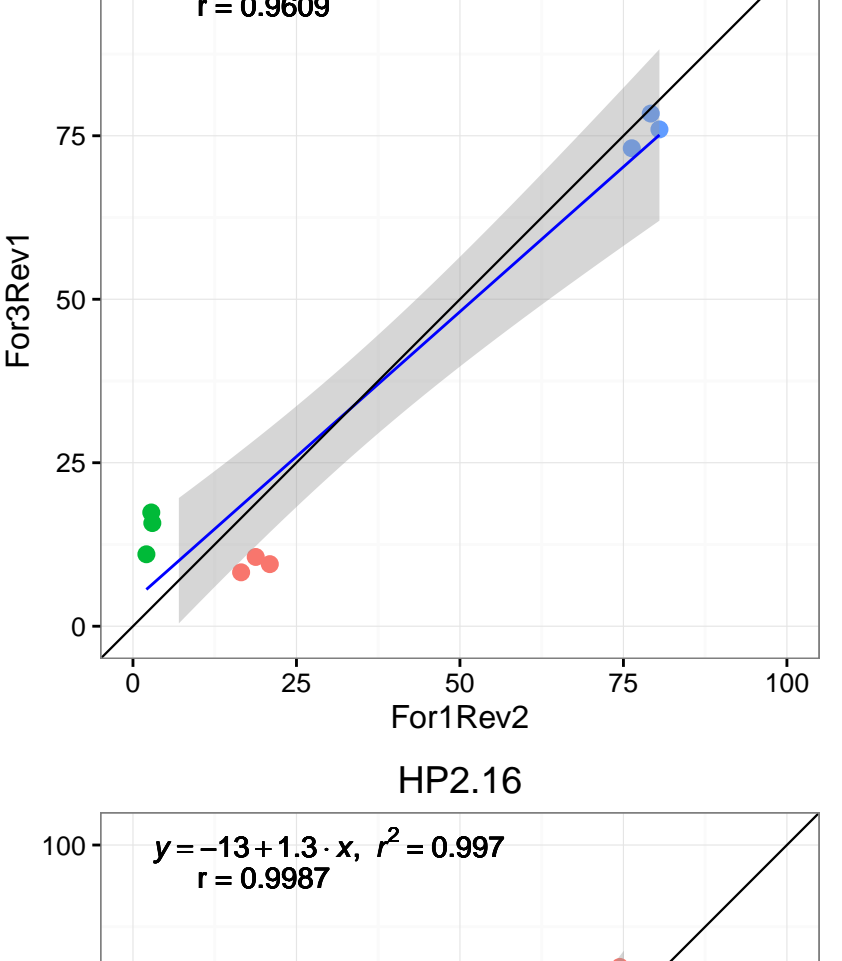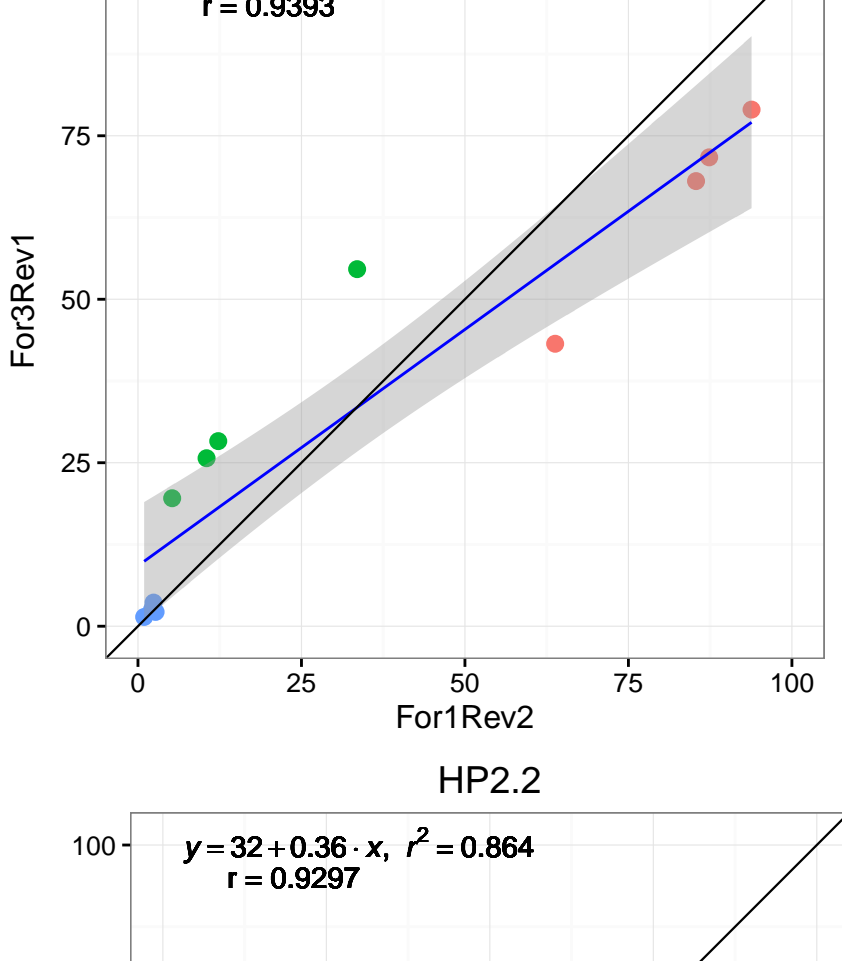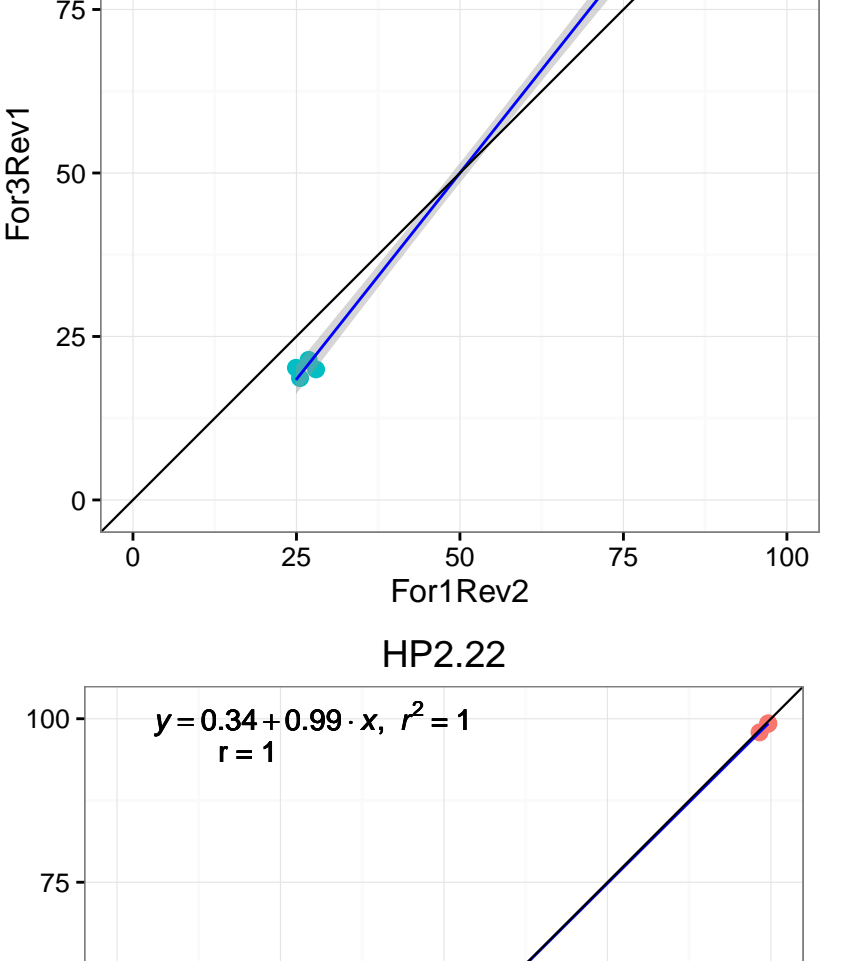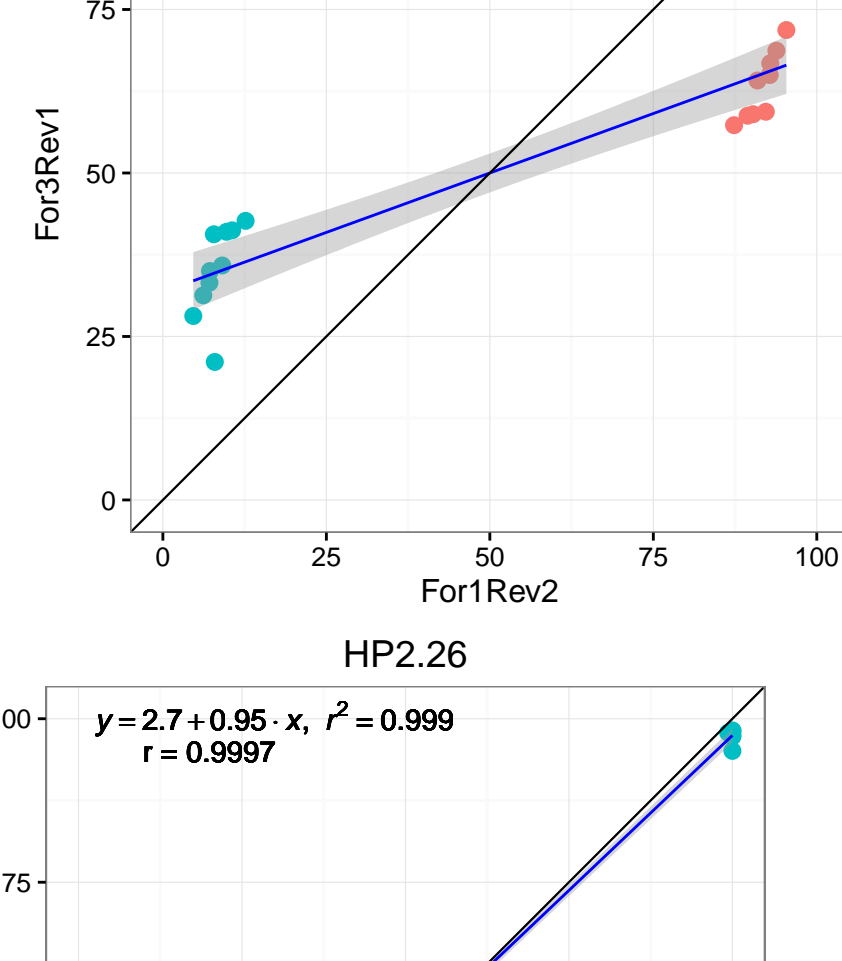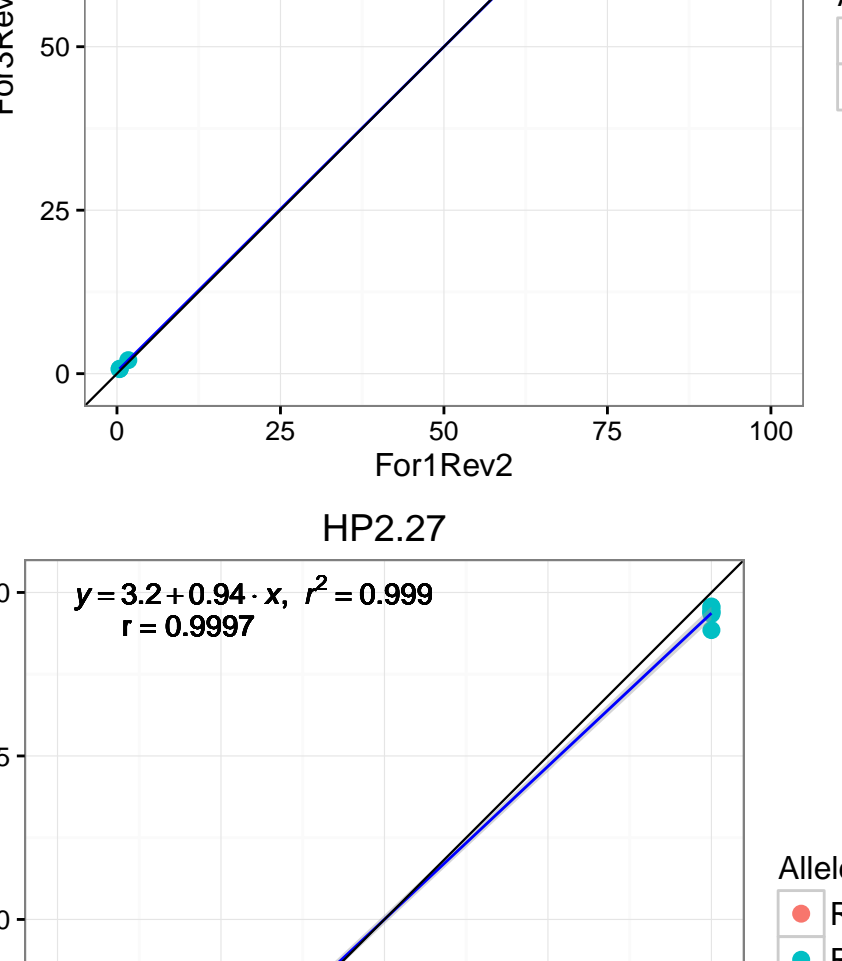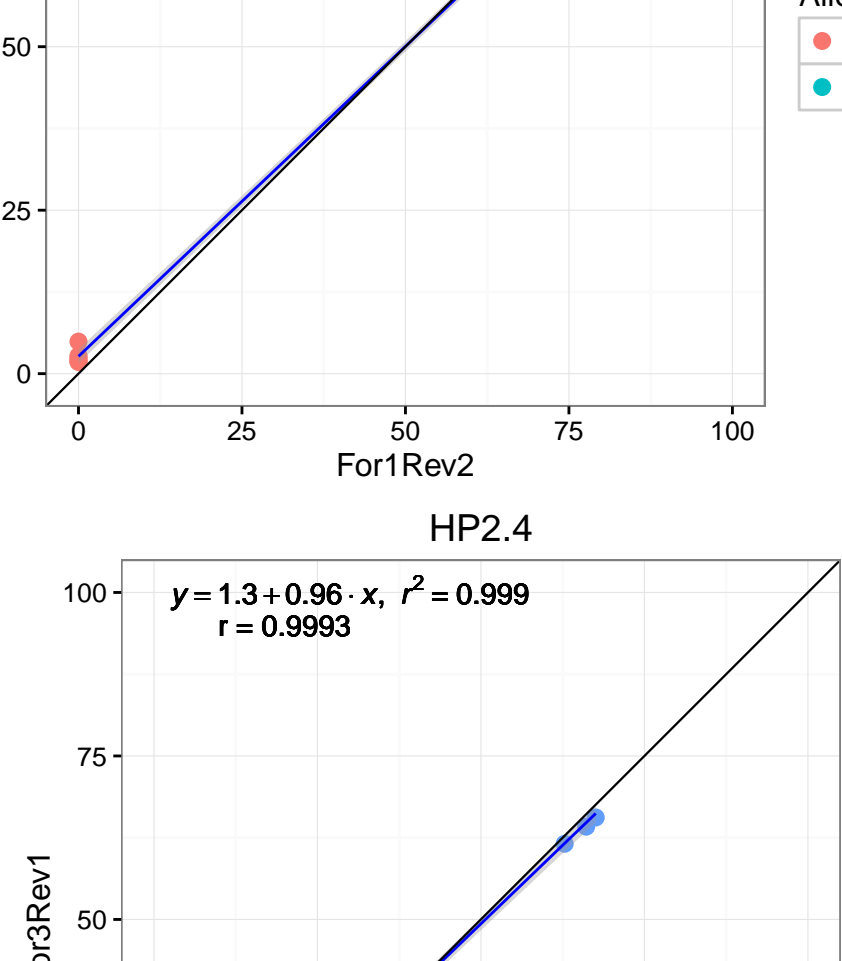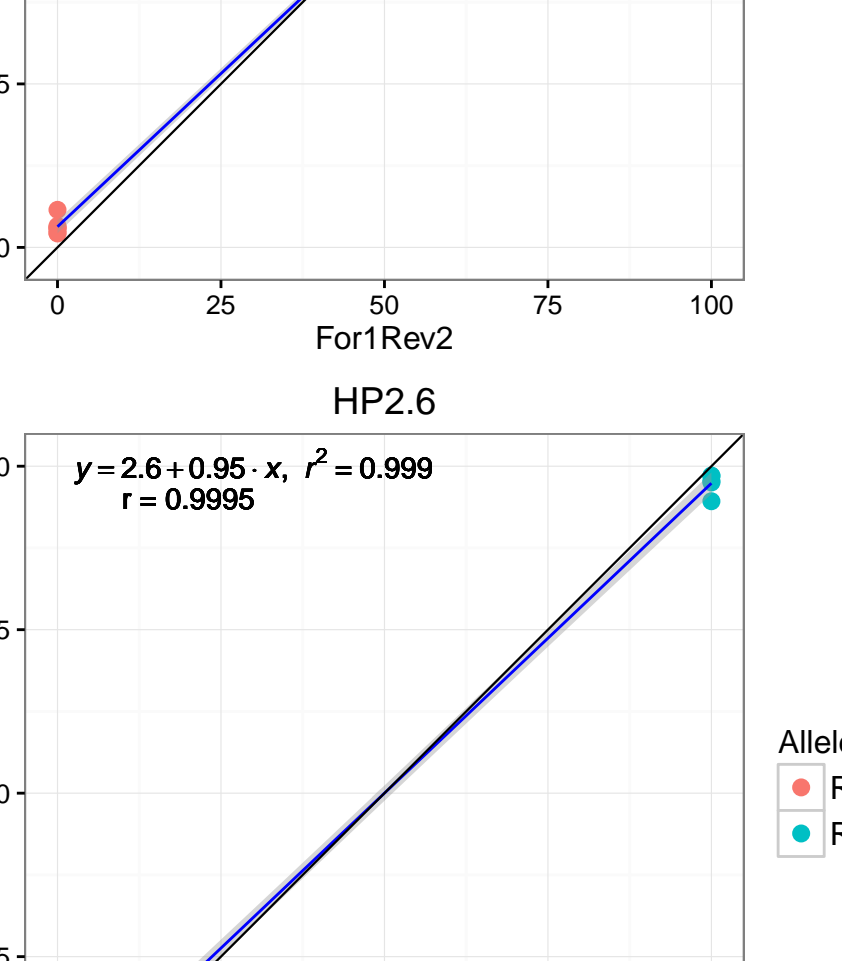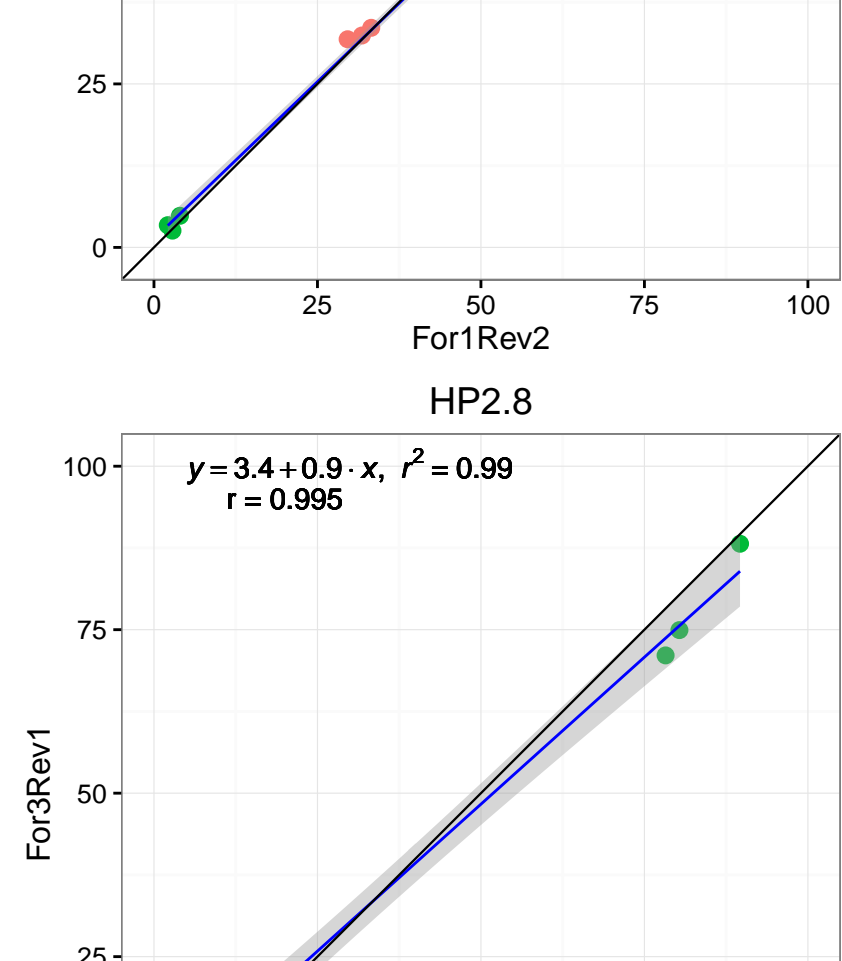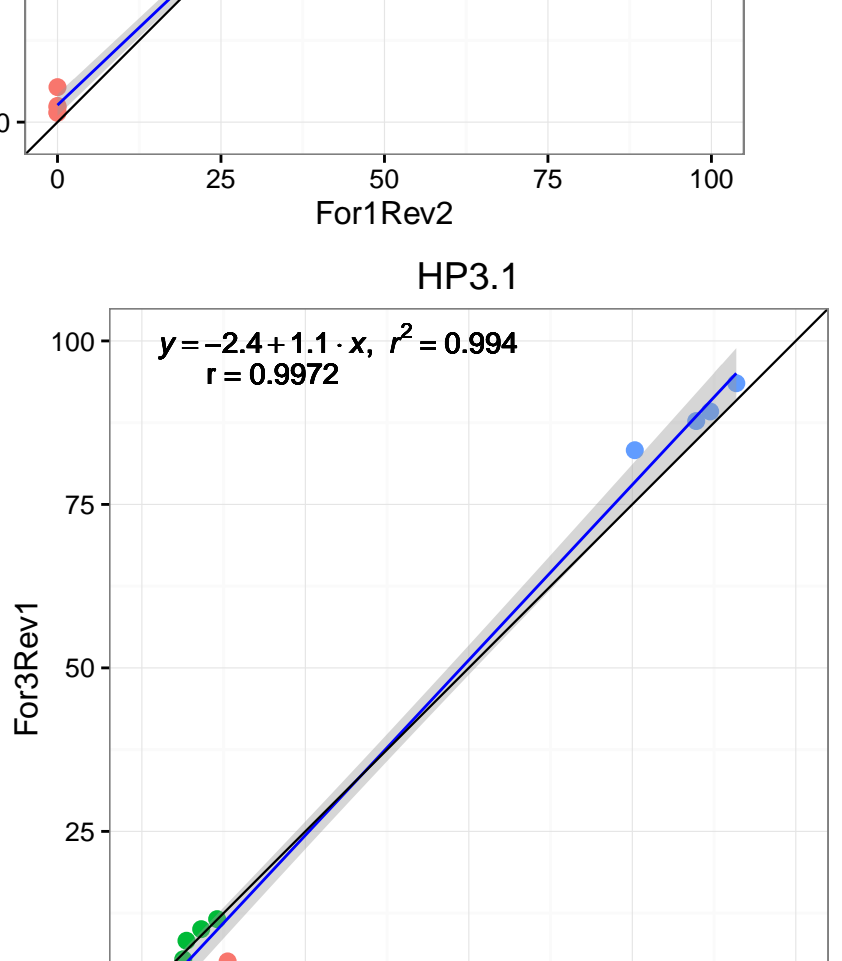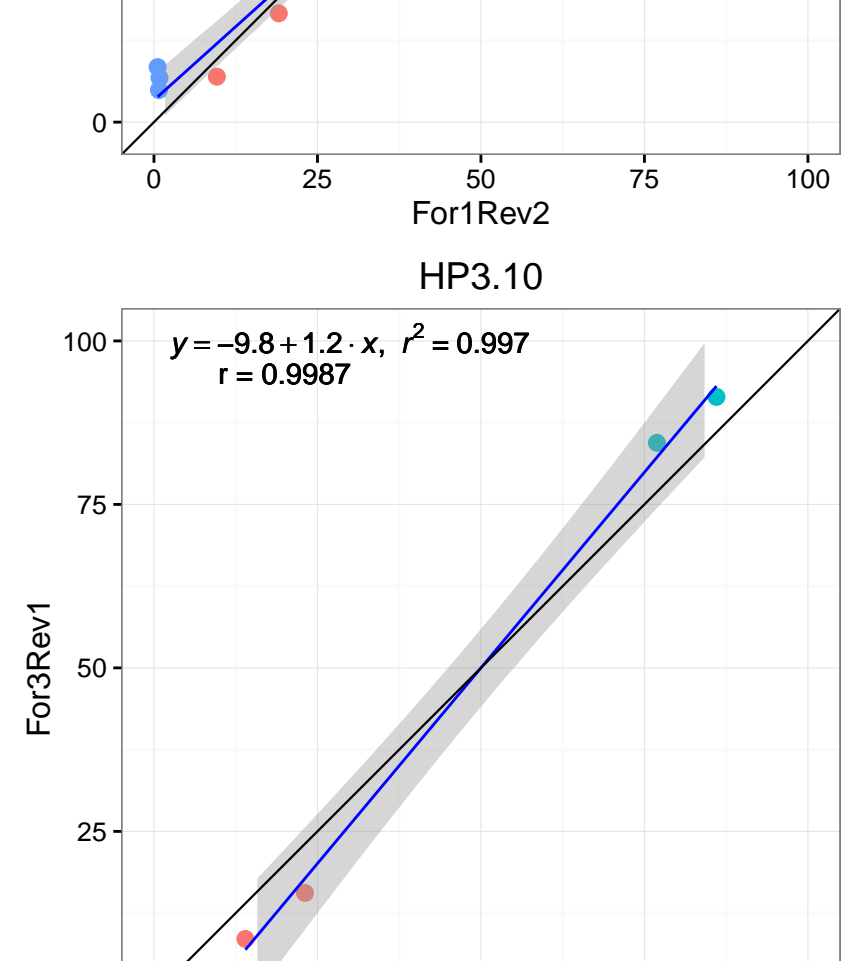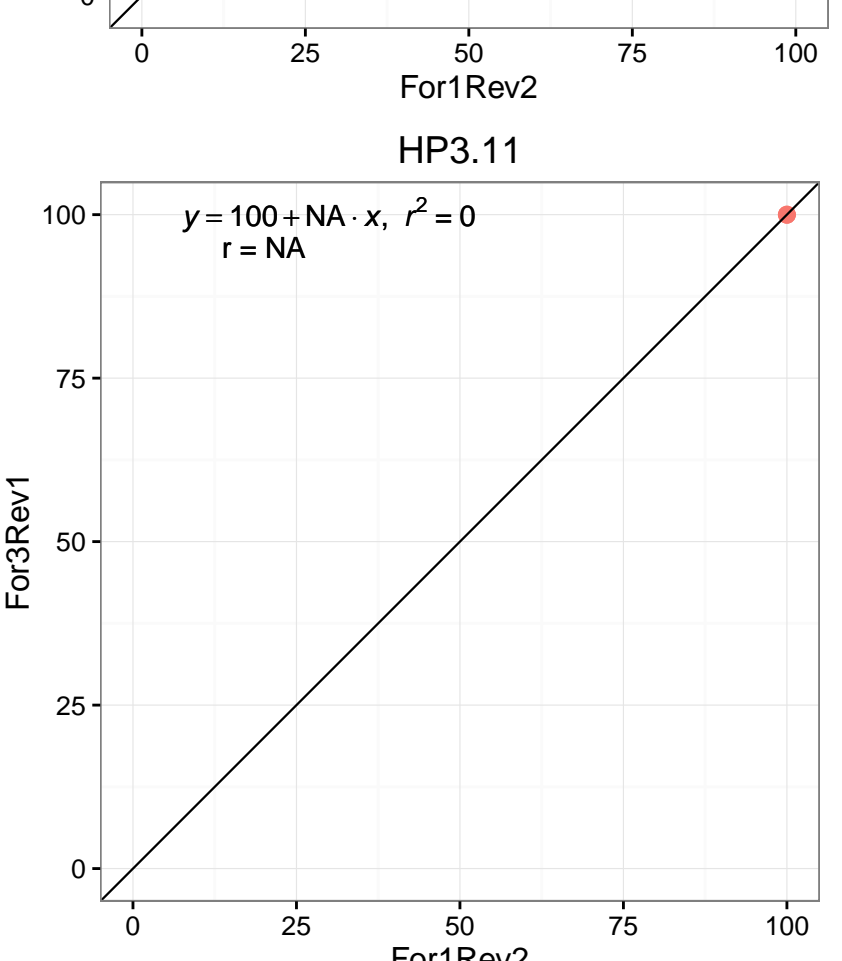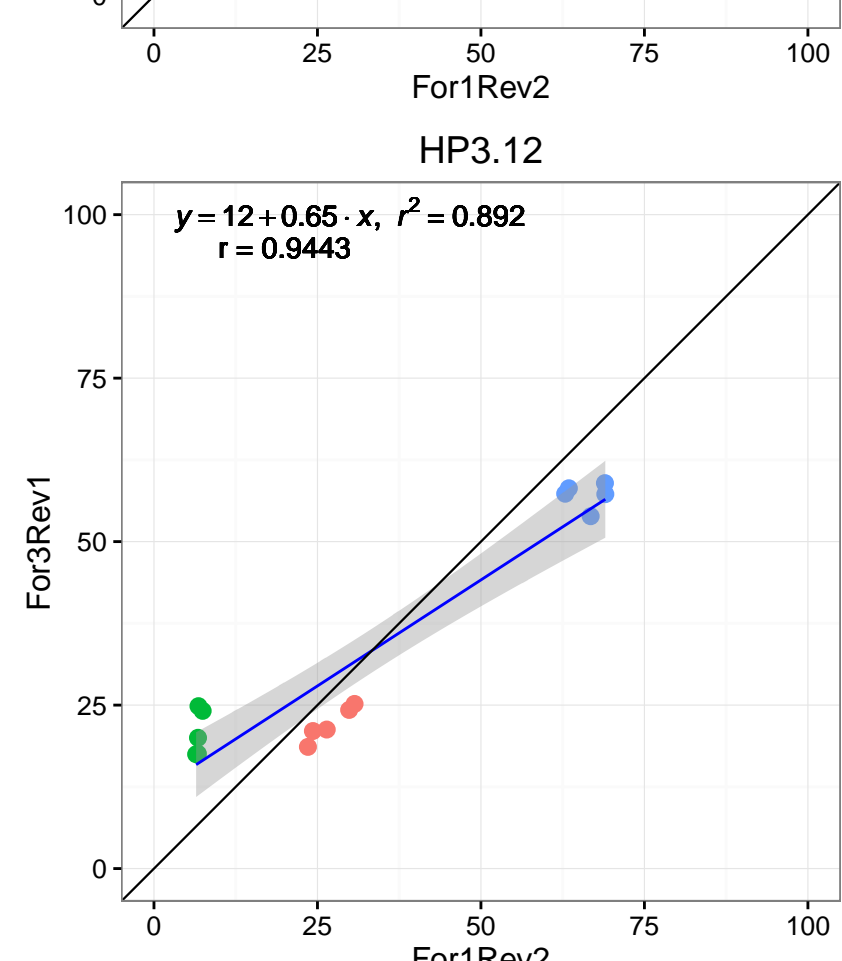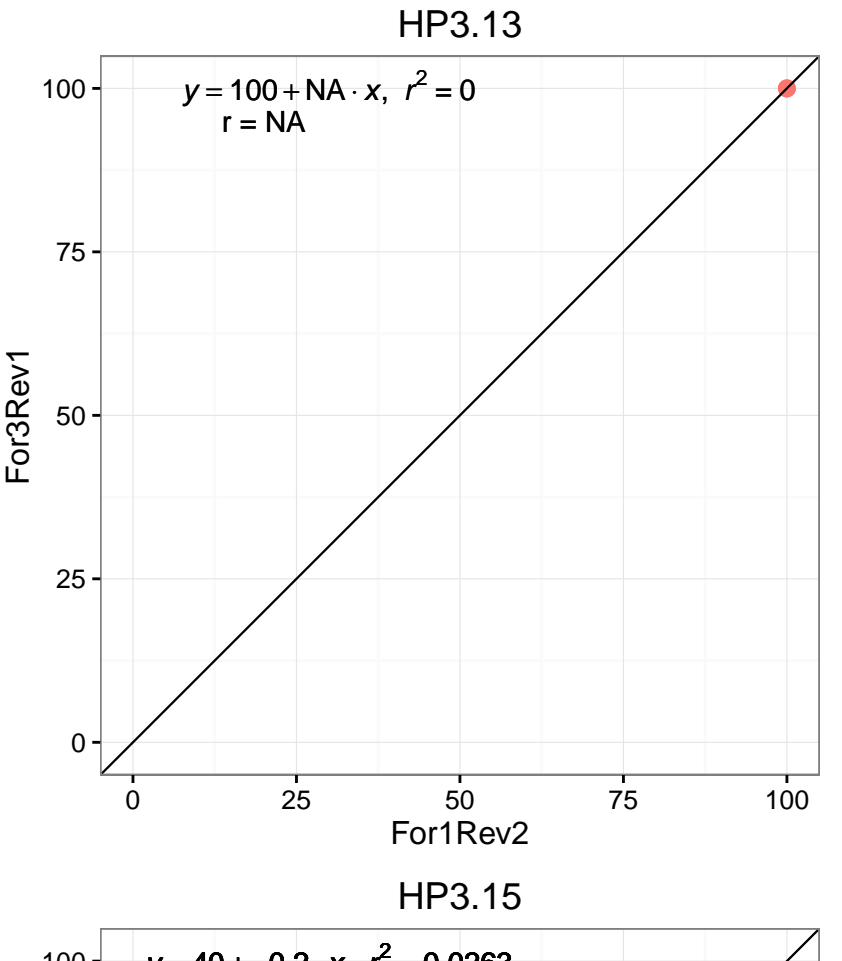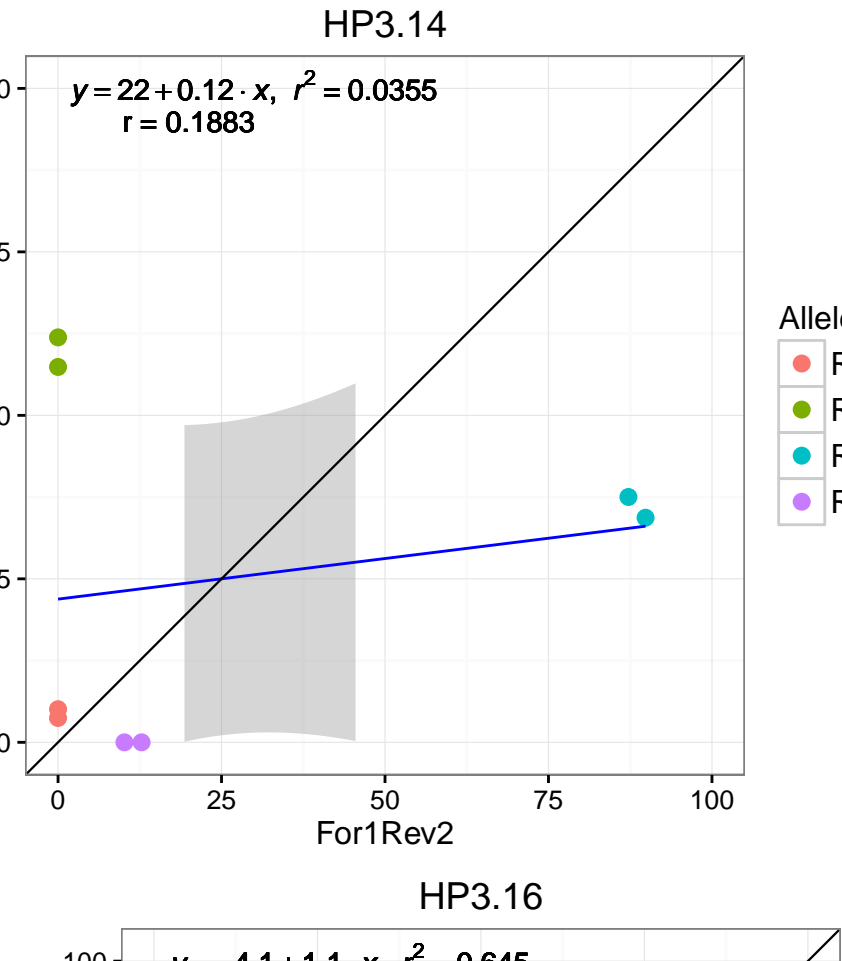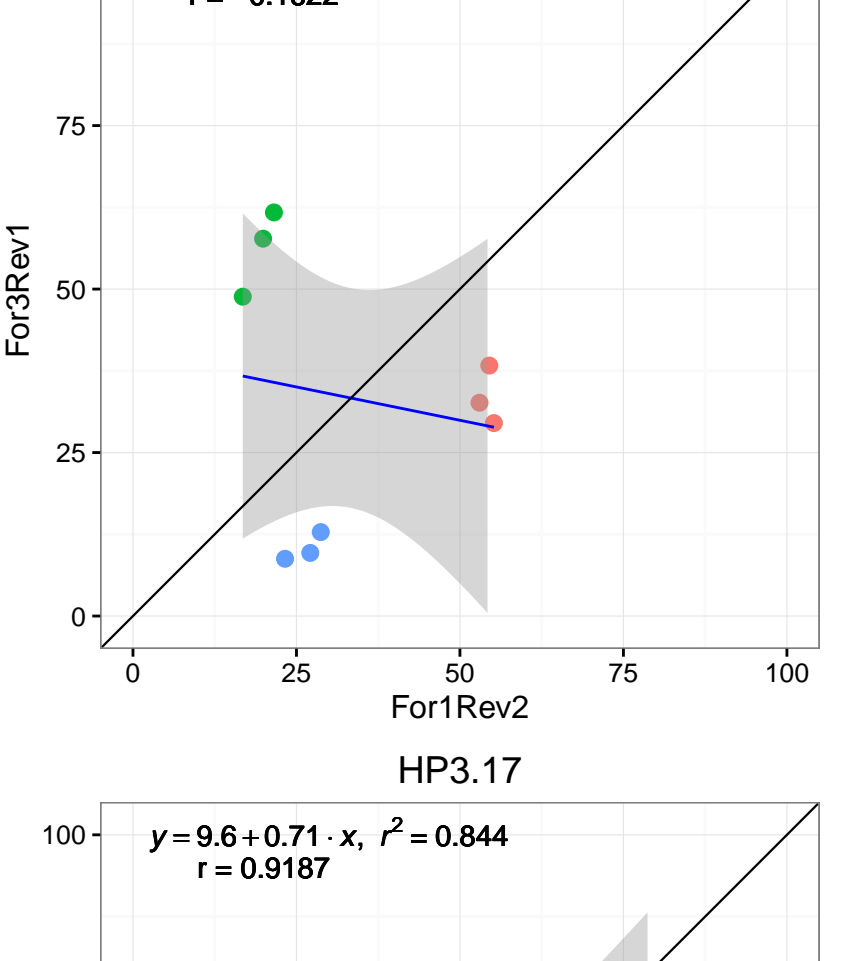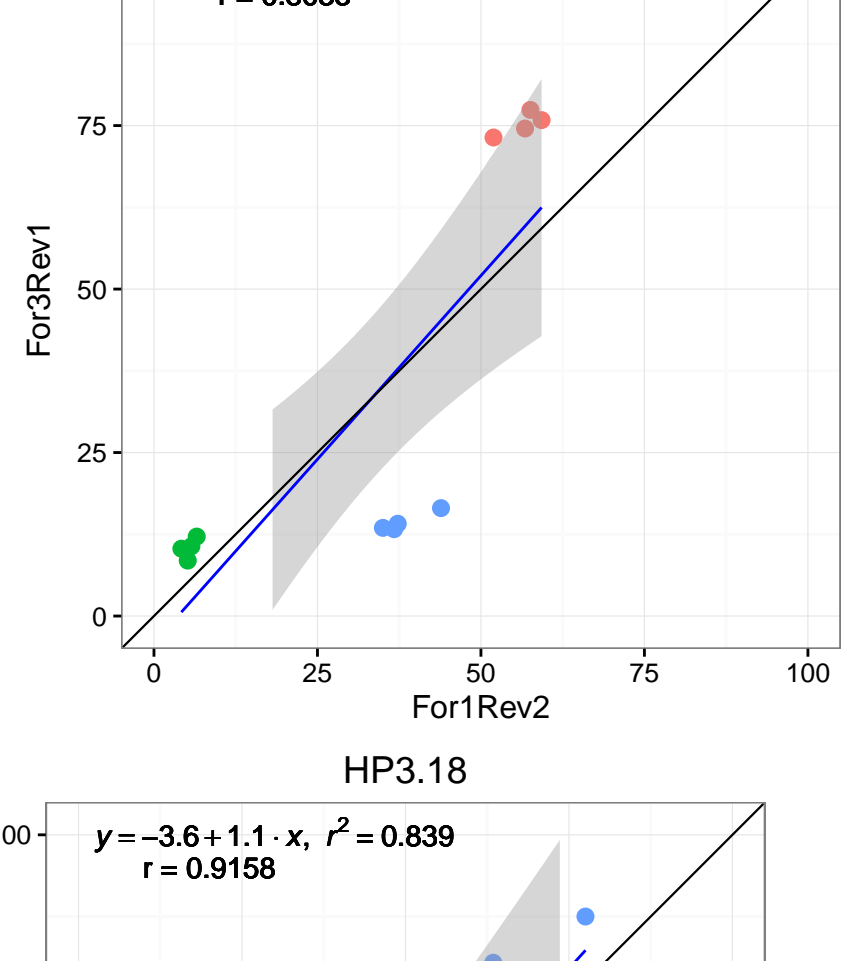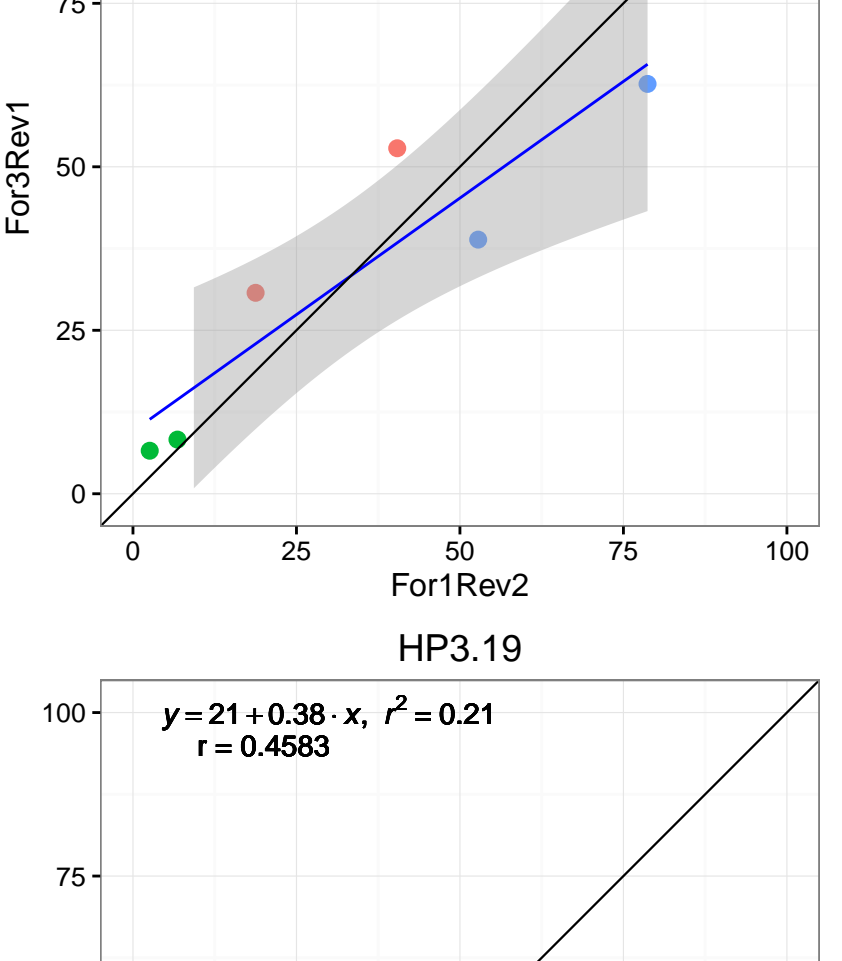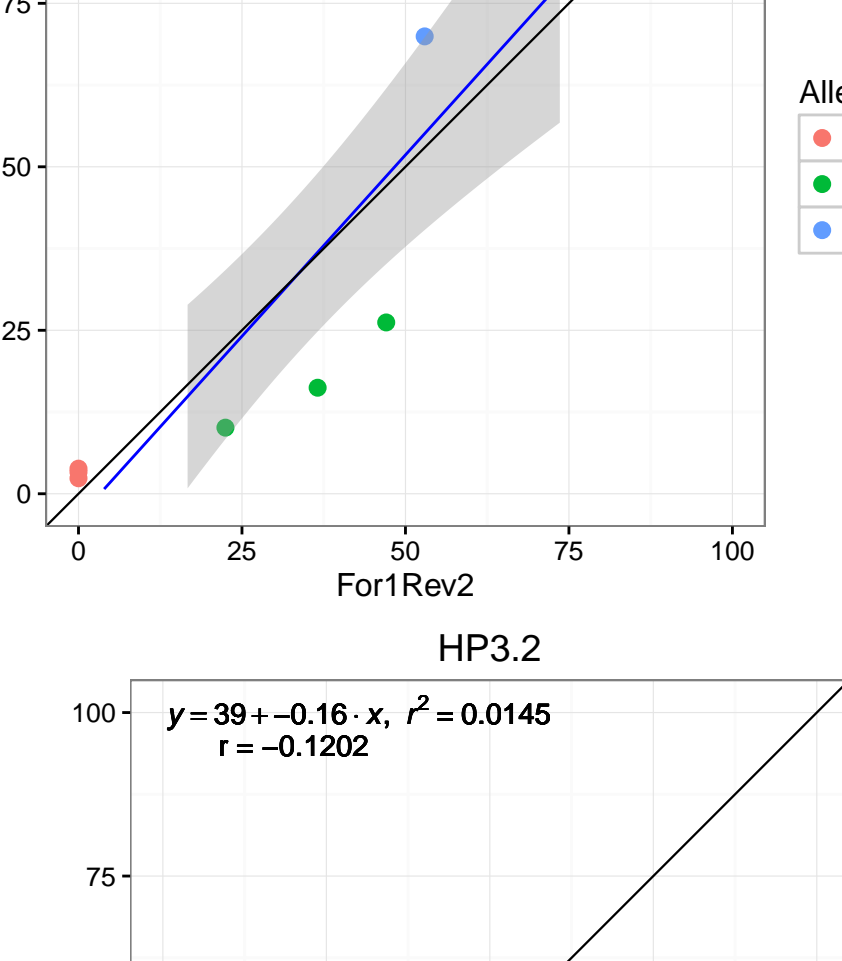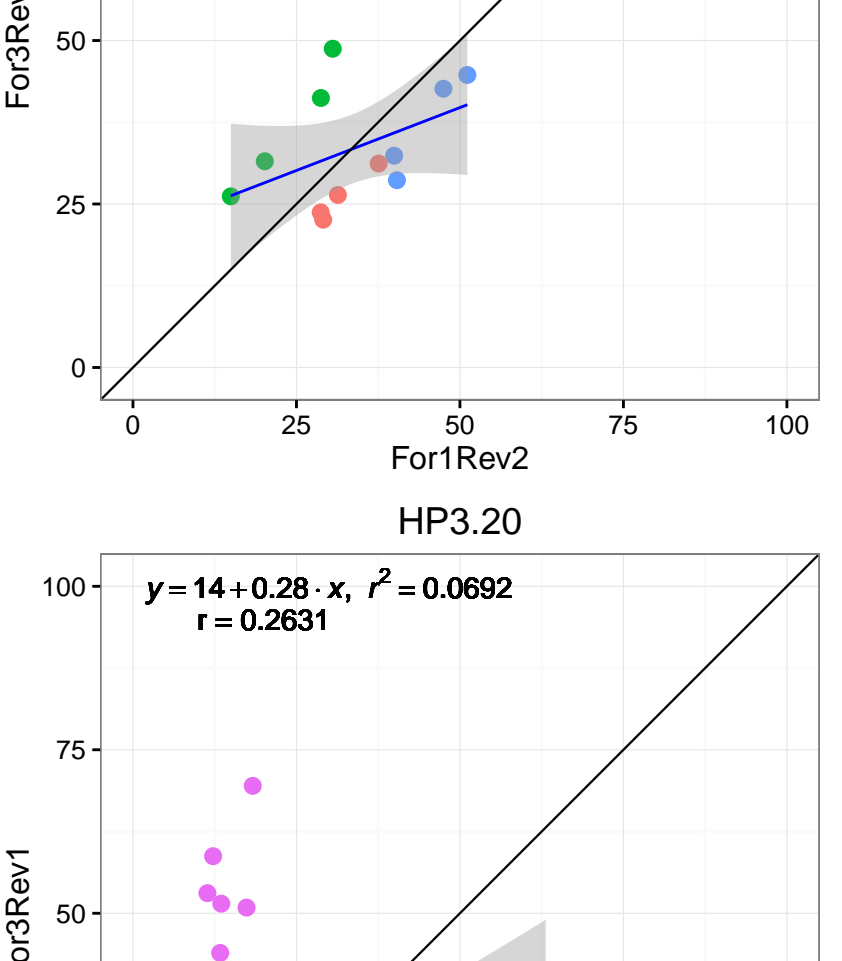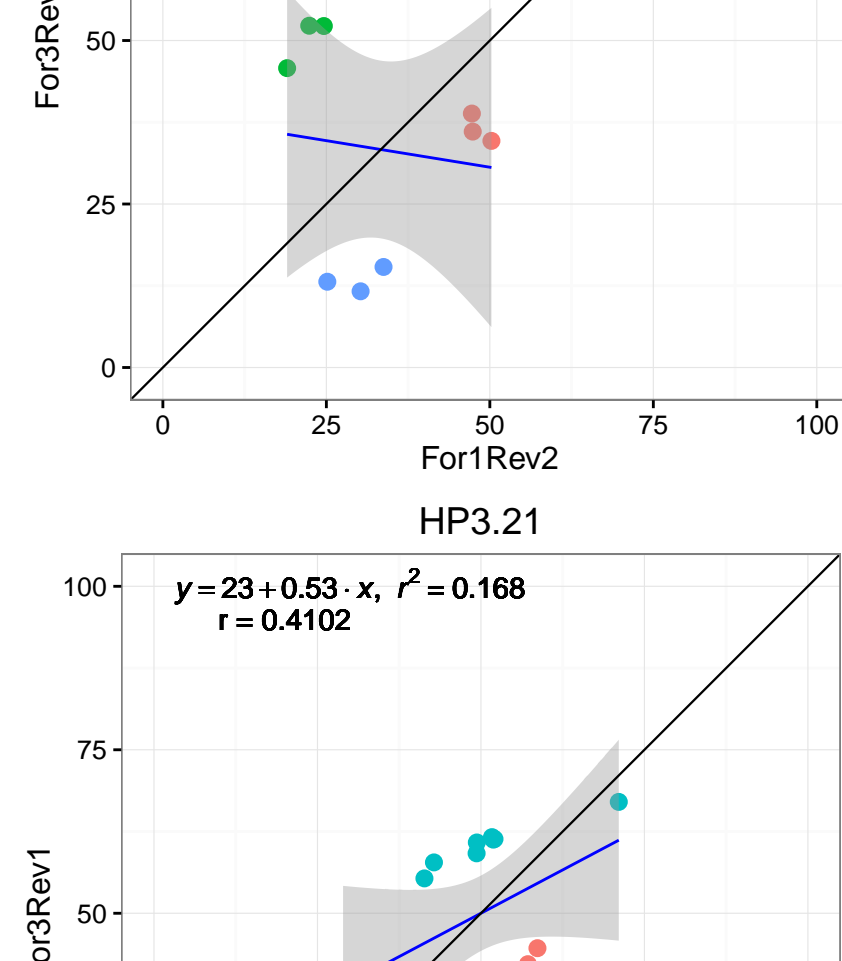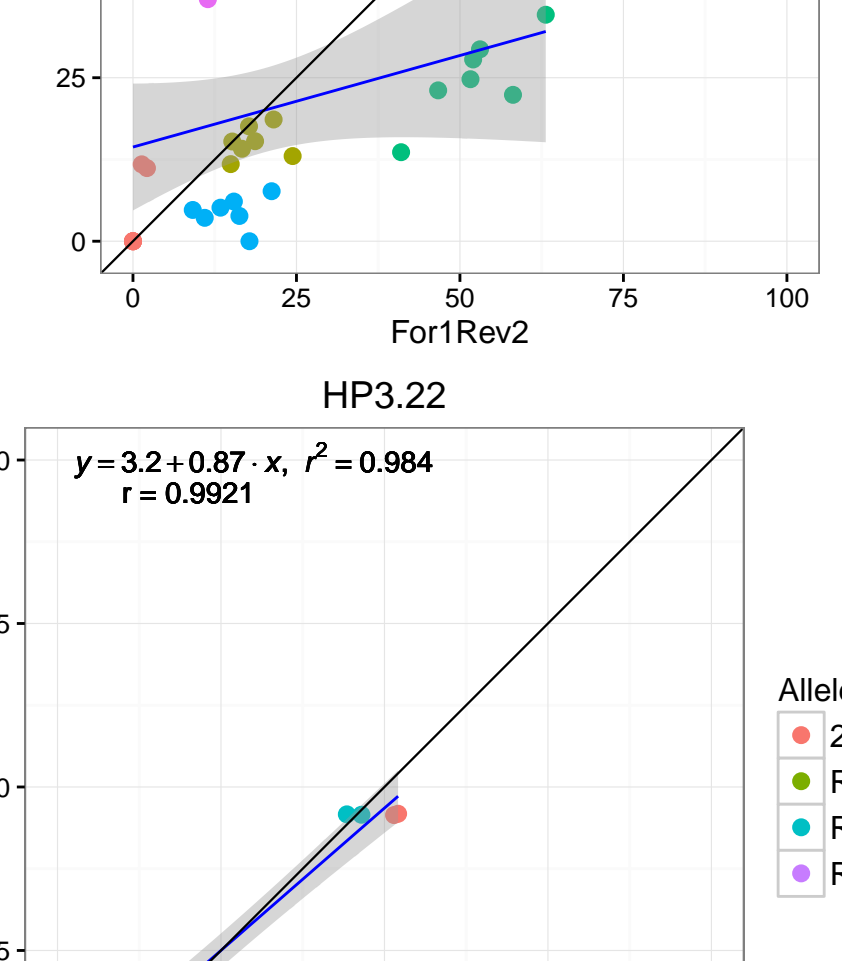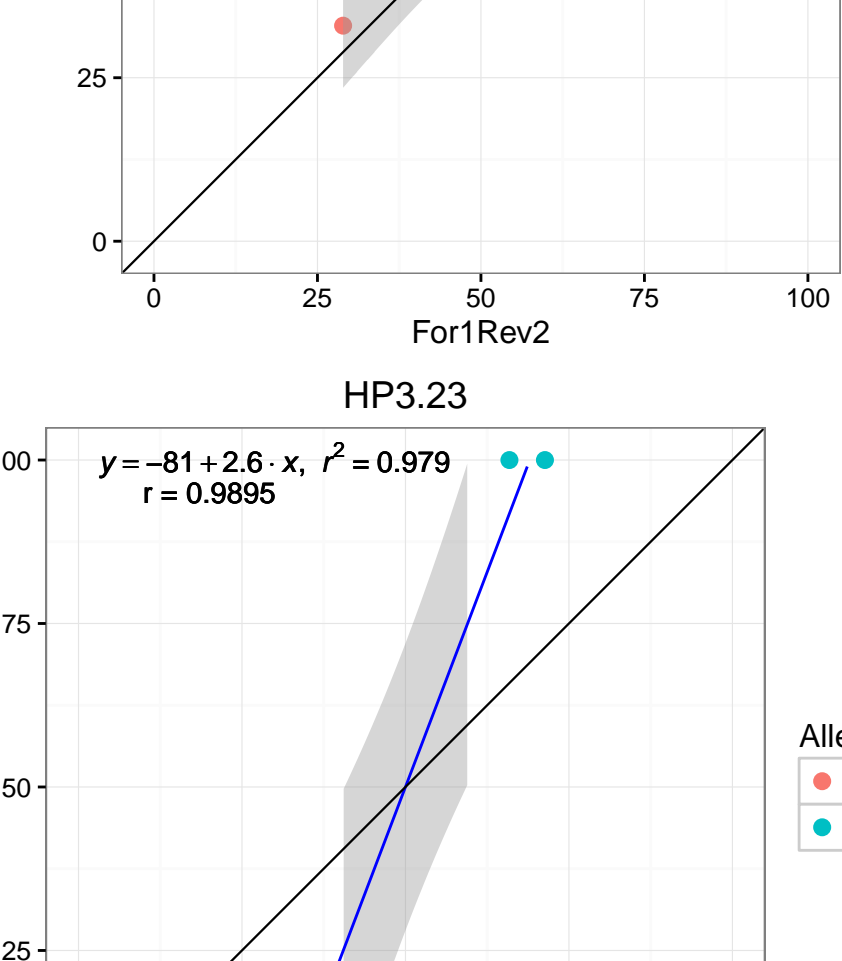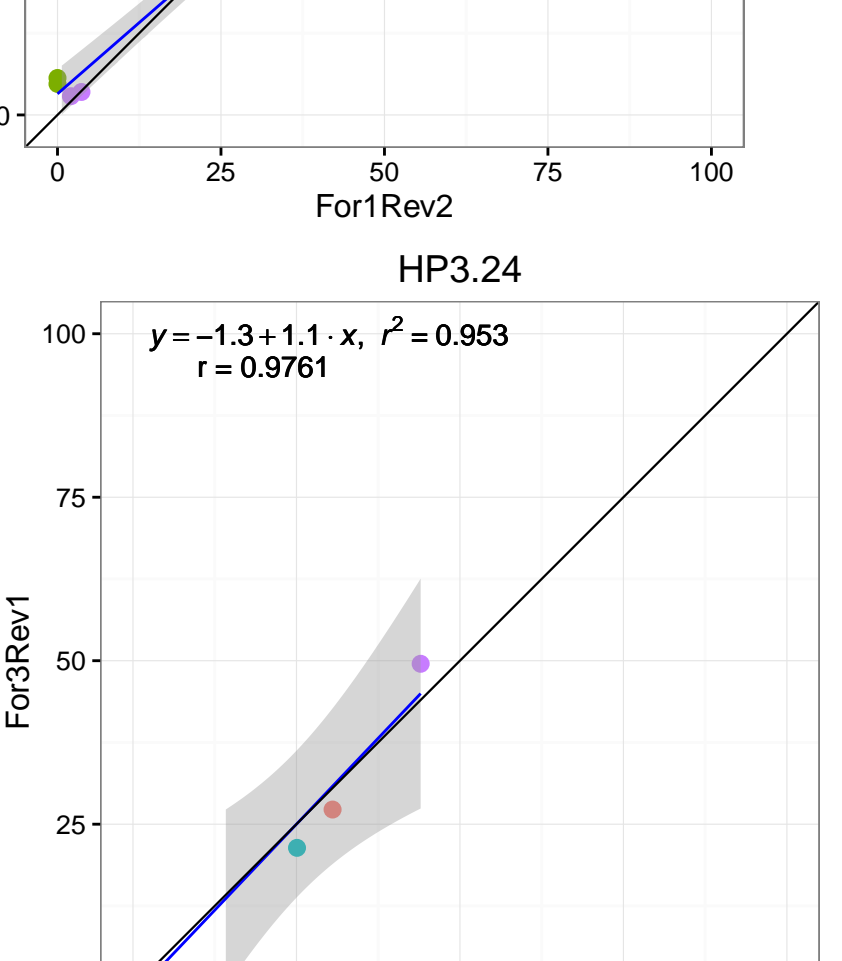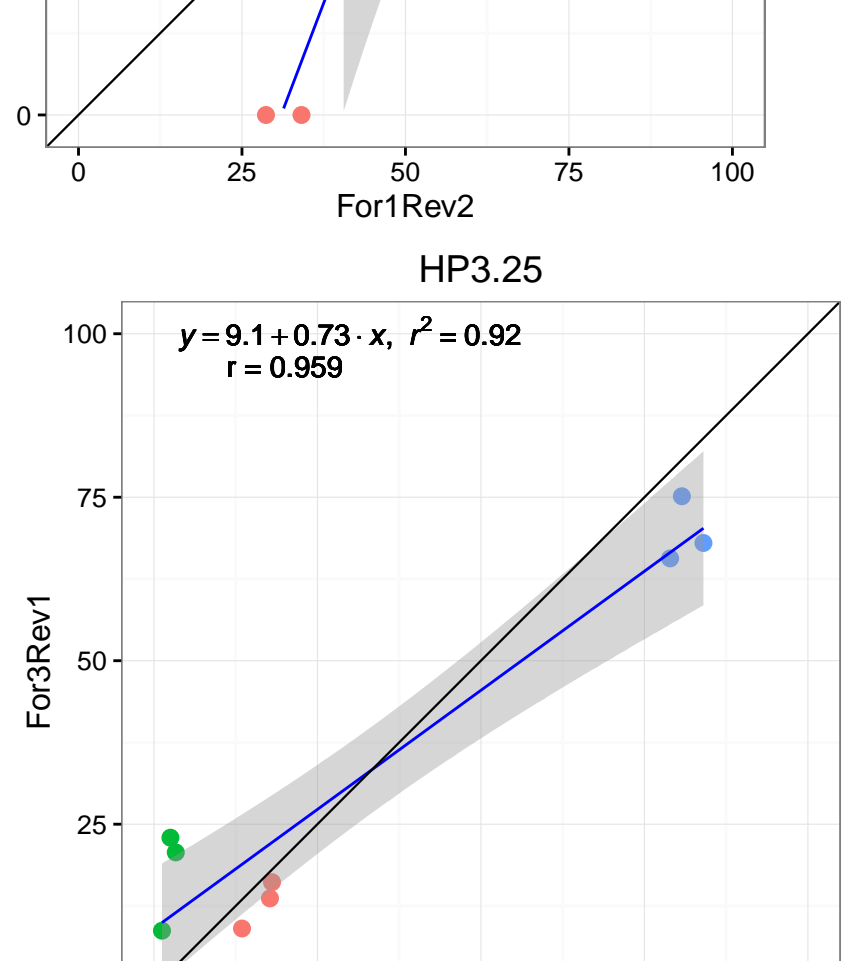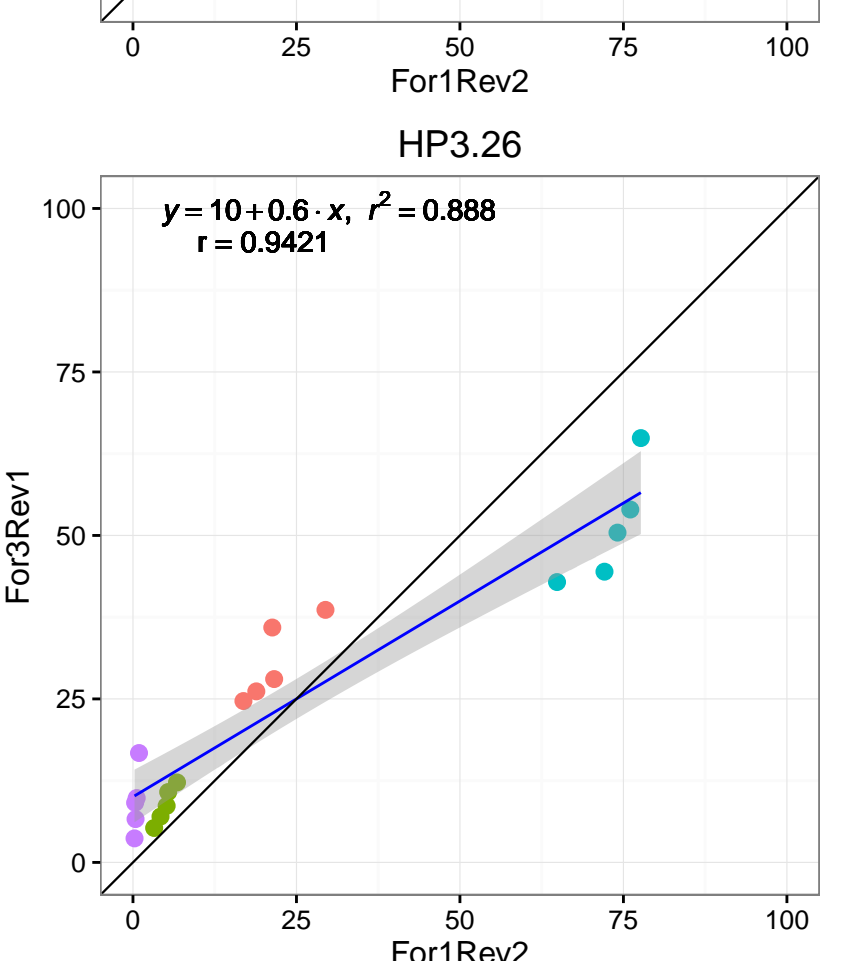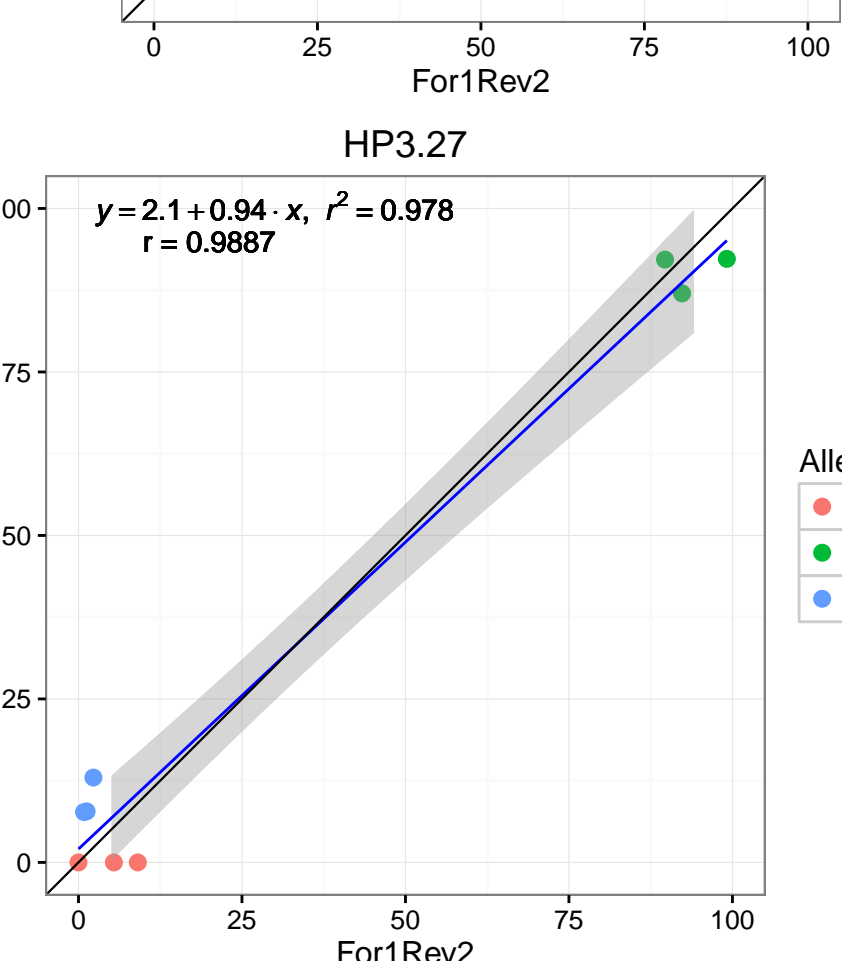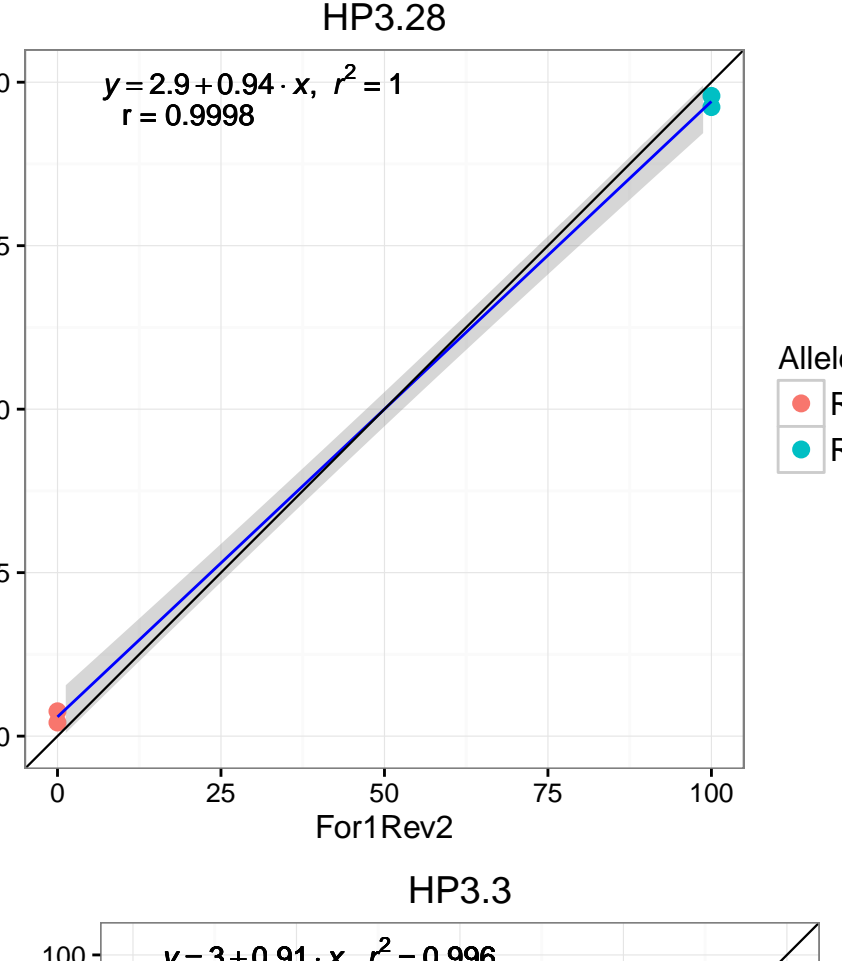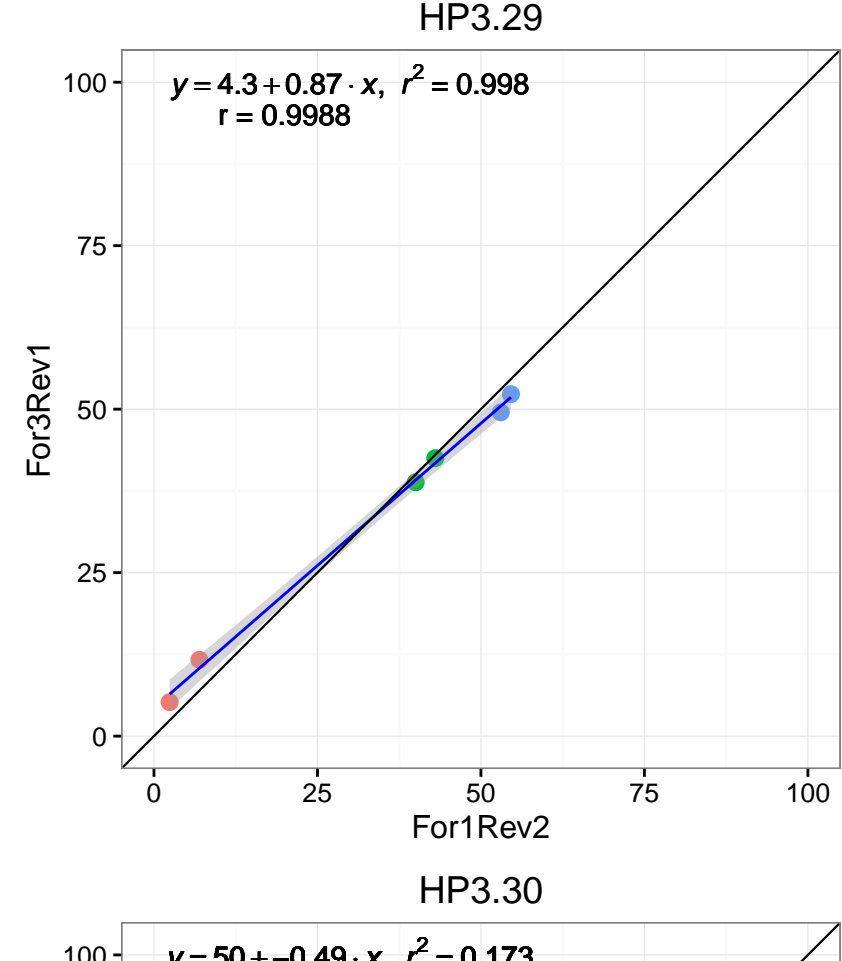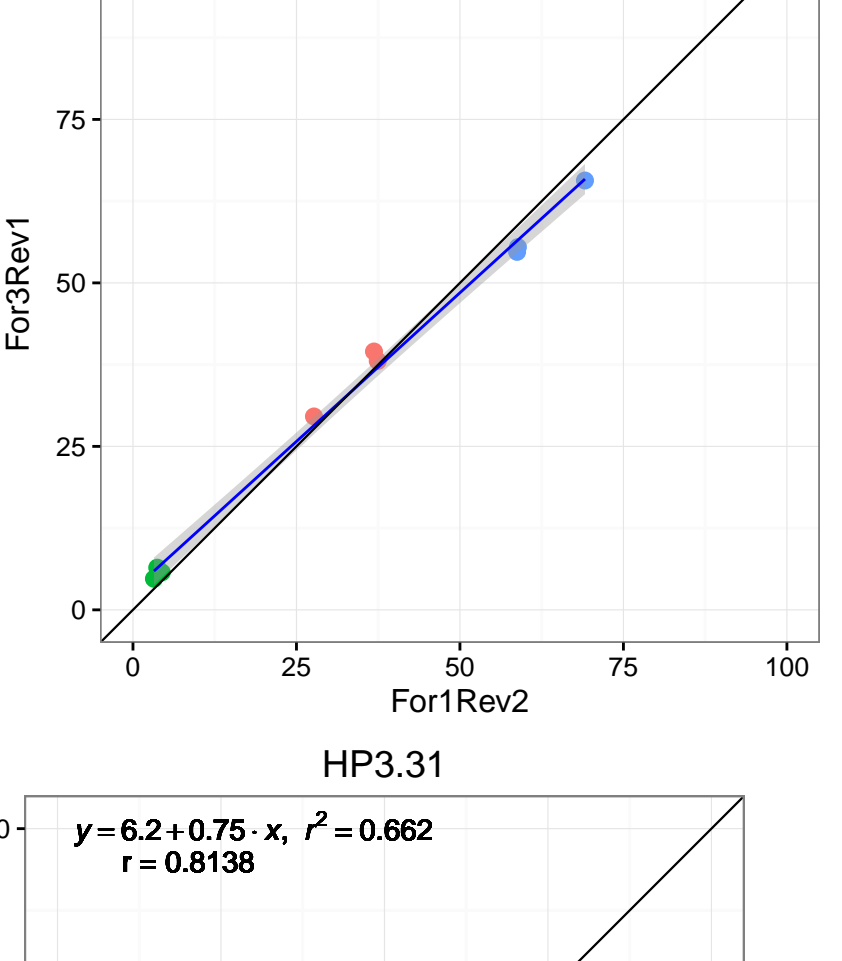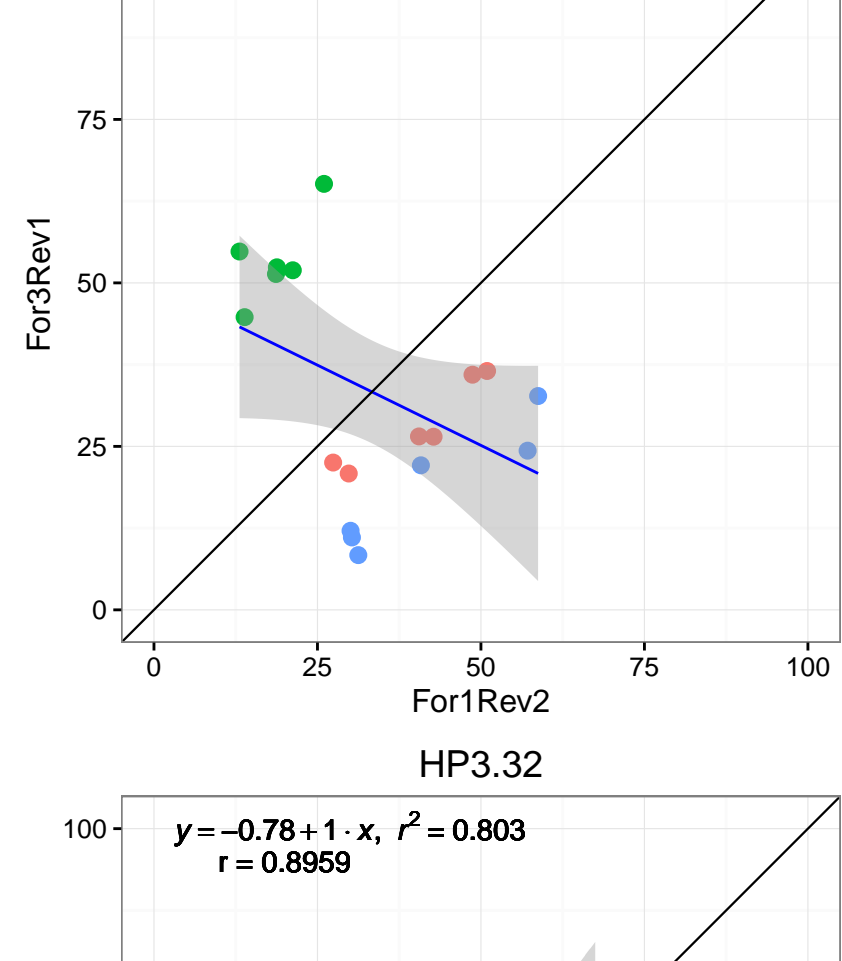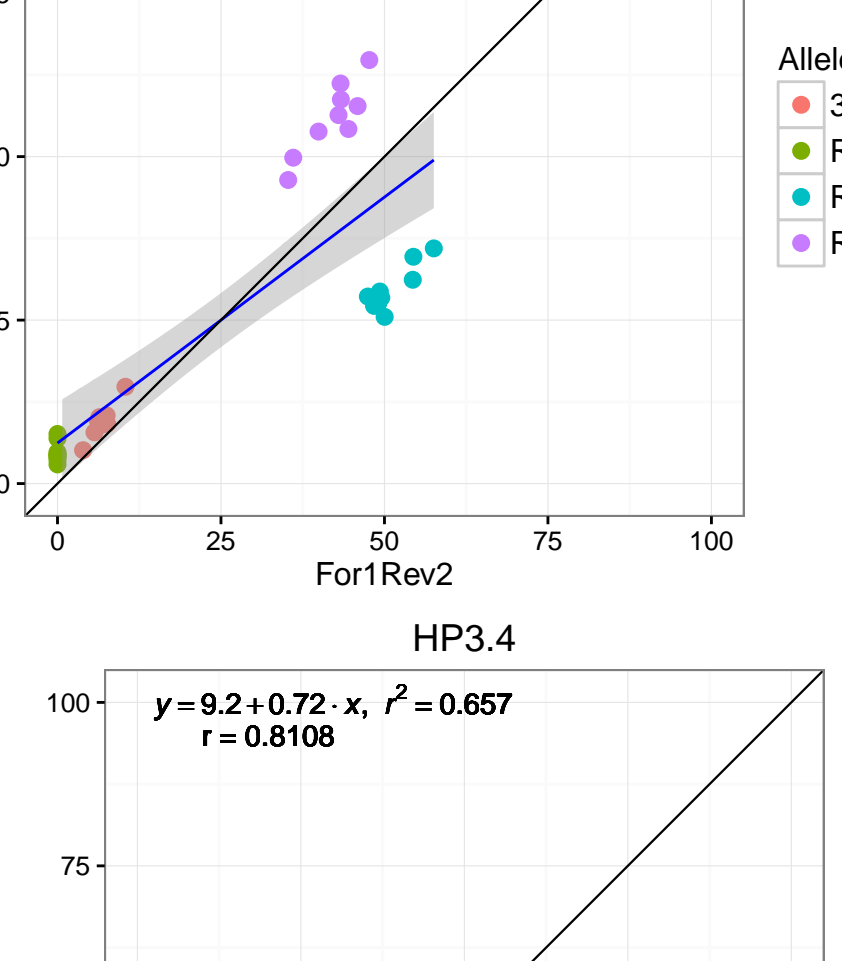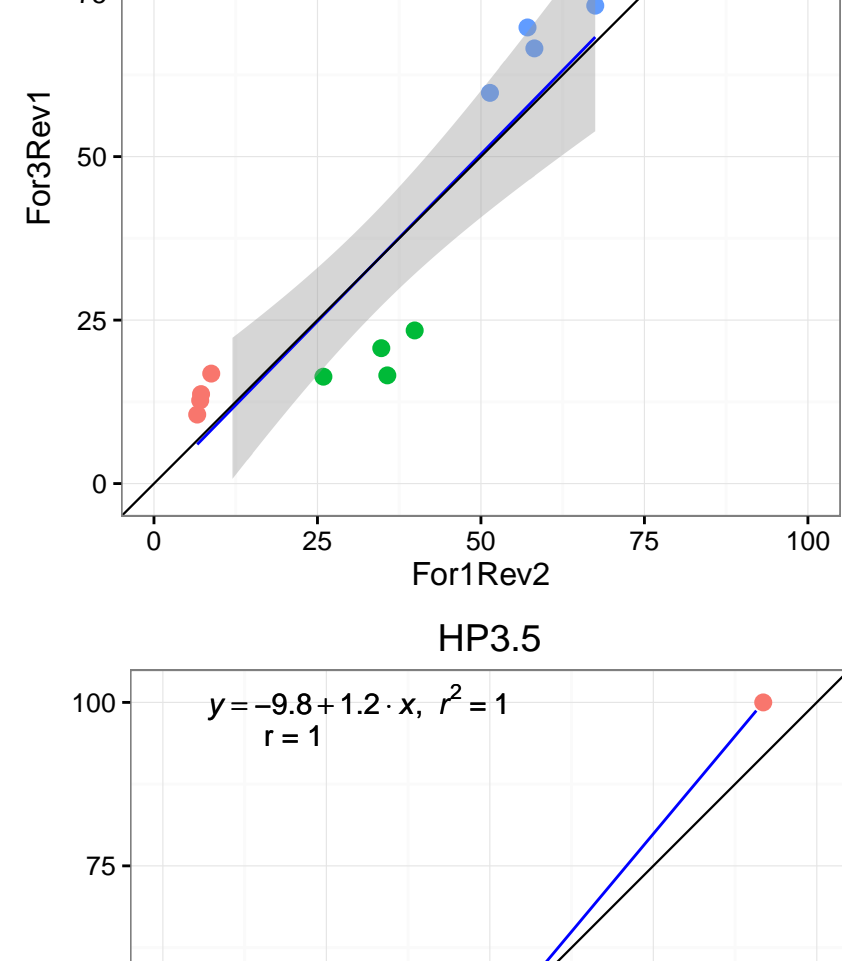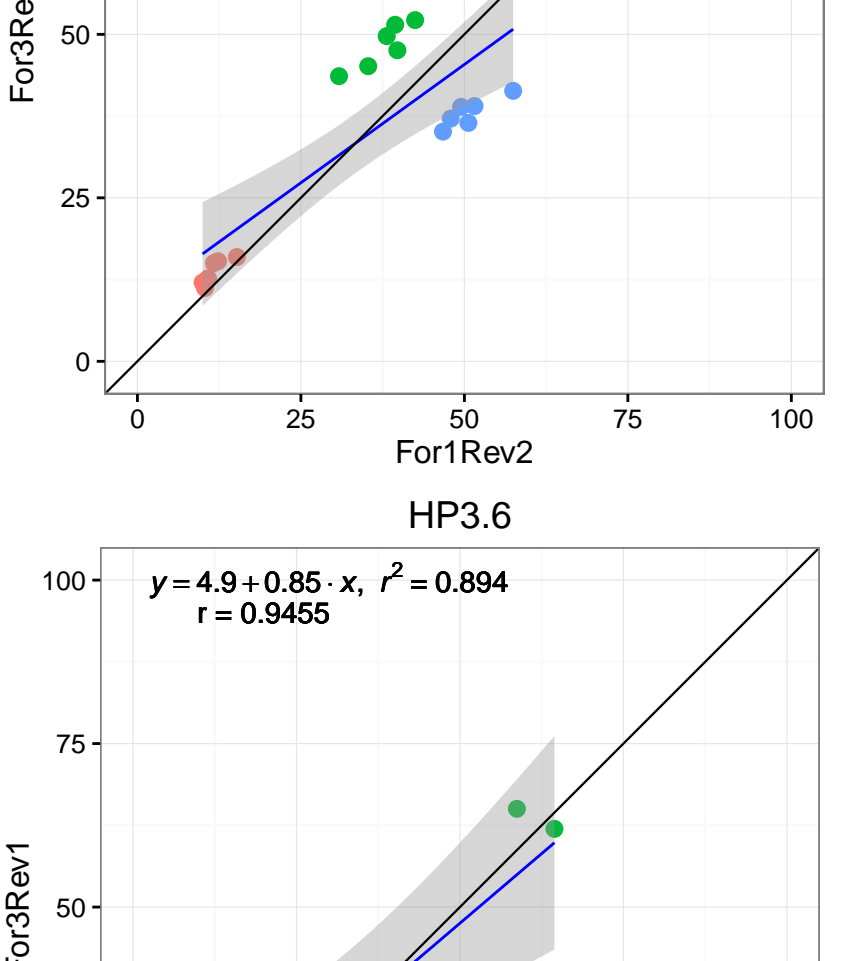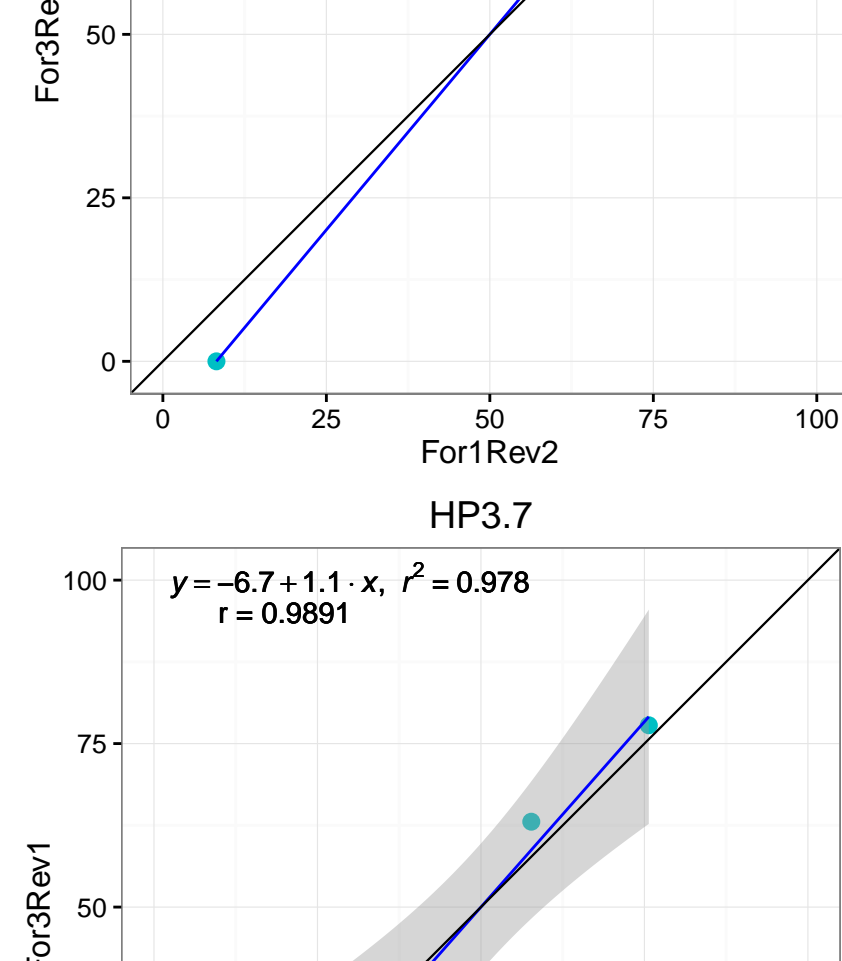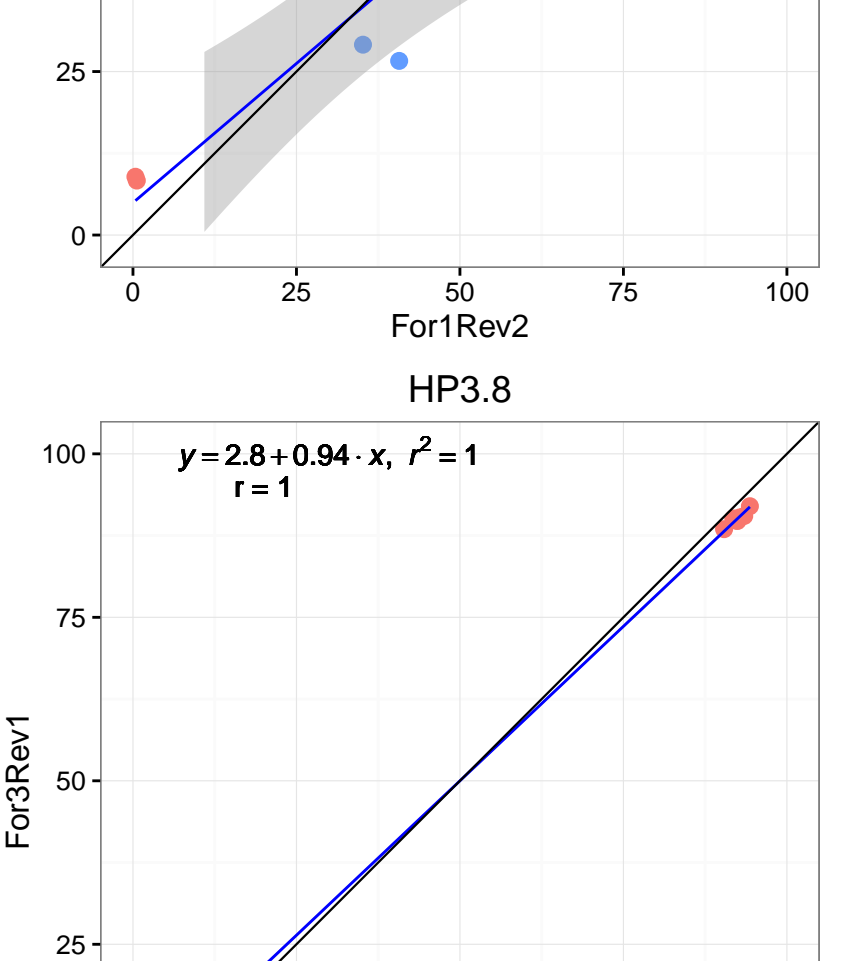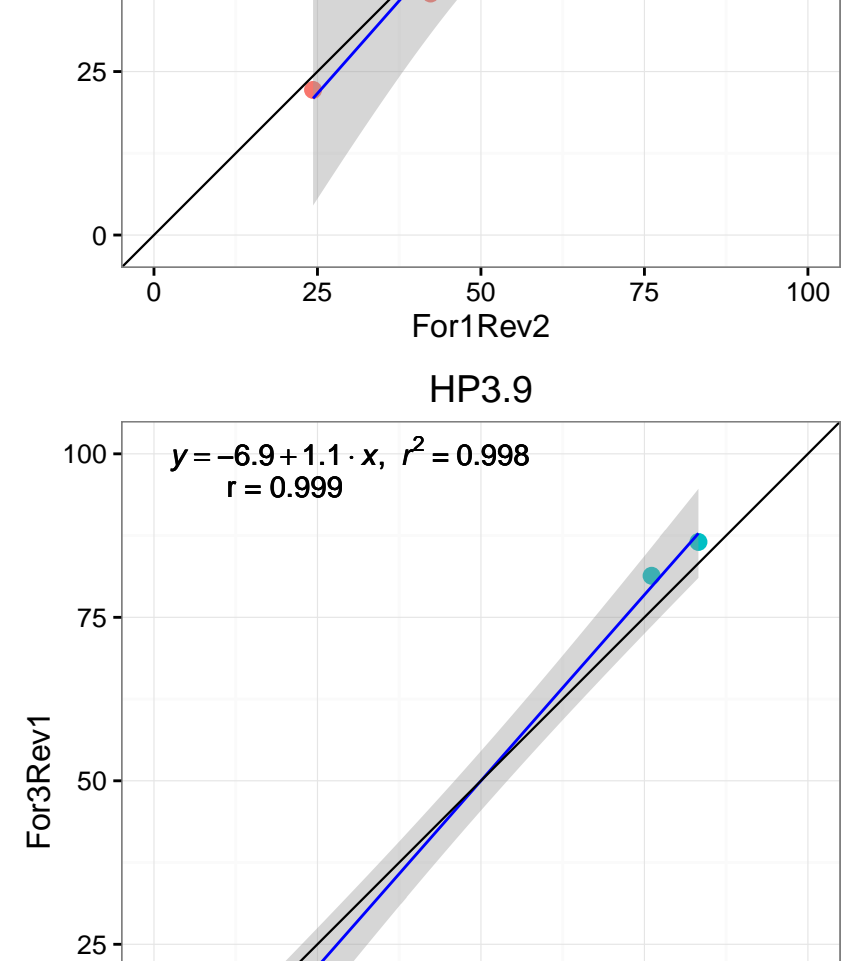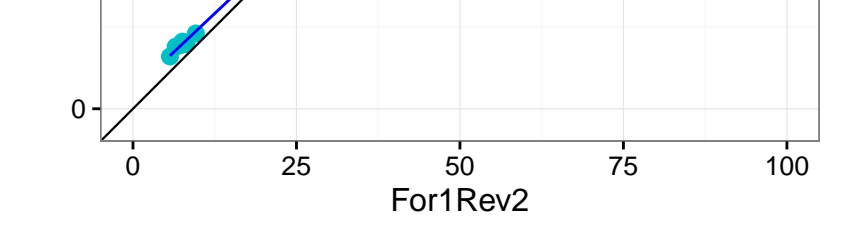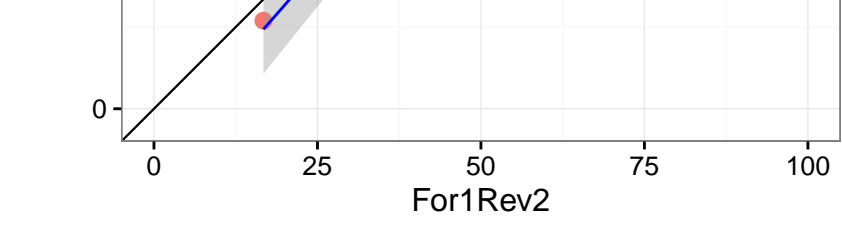

Supplement: Supplementary file 12 — (PDF 102 kb) [file 251_2016_945_MOESM12_ESM.pdf]

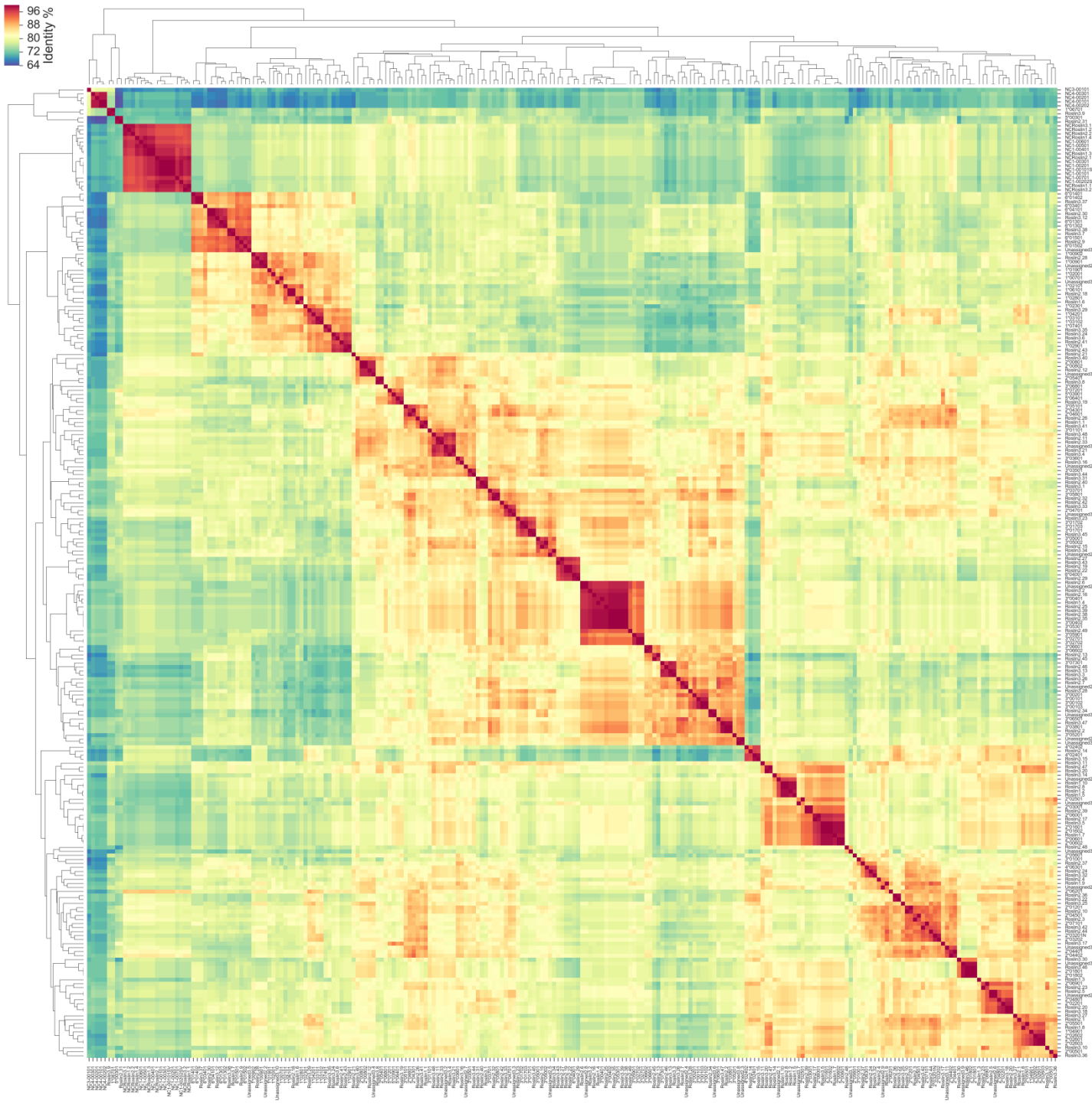

Supplement: Supplementary file 13 — Heat map showing the percentage pairwise identity between the amino acid sequences encoded by the amplified 410 bp nucleotide sequence. Pairwise amino acid identity was calculated with the EMBOSS Needle programme (http://www.ebi.ac.uk/Tools/psa/emboss_needle). The colour key used to create the heat map with the corresponding percentage identity is shown in the legend in the top left corner. (PDF 497 kb) [file 251_2016_945_MOESM13_ESM.pdf]
